# Does a gender of *Welwitschia mirabilis* plants influence their photosynthetic activity?

**DOI:** 10.1371/journal.pone.0291122

**Published:** 2023-09-08

**Authors:** Justyna Wiland-Szymańska, Ewa Kazimierczak-Grygiel, Paweł Drapikowski, Klaudia Borowiak, Maria Drapikowska

**Affiliations:** 1 Department of Systematic and Environmental Botany, The Adam Mickiewicz University, Poznań, Poland; 2 The Botanical Garden, The Adam Mickiewicz University, Poznań, Poland; 3 Institute of Robotics and Machine Intelligence, Poznan University of Technology, Poznan, Poland; 4 The Department of Ecology and Environmental Protection of the Poznań University of Life Sciences, Poznań, Poland; Universidade Federal de Alfenas, BRAZIL

## Abstract

*Welwitschia mirabilis* Hook.f. (Welwitschiaceae, Gnetales) is a gymnosperm plant unique in its habit with an isolated taxonomic position. This species is dioecious, but no studies of its photosynthetic activity were conducted with examination of differences among male and female plants. To fill this gap, the day and night photosynthetic activity of male and female specimens of *Welwitschia mirabilis* cultivated in the botanical garden was studied in controlled conditions. Photosynthetic activity was studied using net photosynthetic rate (*P*_N_), stomatal conductance (*g*_s_) and intercellular CO_2_ concentration (*C*_i_) parameters. Additionally, a normalized difference vegetation index (NDVI) was used to assess the condition among male and female plants in full sunlight. The studied *Welwitschia* plants revealed variability in photosynthetic activity both during the day and the night. The photosynthetic activity was low in the morning hours and higher in the afternoon. There is a difference in the photosynthetic activity during the night between sexes, being higher in female specimens. Stomatal density was evaluated separately for adaxial and abaxial leaf surfaces. Statistically significant differences in the stomatal density on abaxial and adaxial leaf surfaces were observed in both sexes, especially distinctive in female specimens. NDVI has revealed that there were weak differences between male and female plants.

## Introduction

*Welwitschia mirabilis* Hook.f. (Welwitschiaceae, Gnetales) is among the most interesting gymnosperms of an isolated taxonomic position and ancient origin [1]. It is characterized by an unique habit, as an almost subterranean tree with a short and stout trunk bearing only two leaves with a permanent growth through its lifetime. Its large taproot penetrates soil to ca. 3 m. This species is known to live up to 2000 years, reaching height of only 1,8 m above ground. Its distribution is confined to a narrow coastal strip in the Namib Desert from Kuiseb in South West Africa to Cabo Negro in Angola [2]. This species is endangered and strictly protected in the wild and is included in Appendix II of the Convention on International Trade in Endangered Species of Wild Fauna and Flora [3]. *Welwitschia* is cultivated in botanical gardens, but it is difficult to grow, due to its unique bauplan and special habitat requirements.

So far, not much stress has been placed on differences among male and female plants of *Welwitschia* in terms of their physiology and anatomy of the vegetative organs. The female and male inflorescences were studied by Sykes [4] and Pearson [5] and the species was regarded as dioecious. More detailed studies on sexual organs revealed that male plants form structurally bisexual flowers [2, 5, 6]. However, the inflorescences and therefore plants are functionally unisexual (male or female). Dimensions and number of inflorescences in the wild were studied by several authors [7–9]. A female may produce from 10 000 up to 20 000 seeds [10, 11]. In nature female and male plants start to flower in this same period (September-October) and pollination takes place between November and January, but seeds ripen till March. After pollination period, the male plants are past anthesis and in a vegetative state, while female plants need to support developing embryos for at least another two month. This creates differences in resource allocation among sexes. The seeds collected in natural localities exhibit a high rate of infestation with *Aspergillus niger* F.V. Tjeghem, which inhibits their viability [2]. The seed germination rate *ex situ* is tolerably high [12], but plantlets exhibit low tolerance to transplanting. Cultivated *Welwitschia* plants can flower as early as two and a half years after germination [13], but from our observations male plants flower at age of at least 5 years, and female plants of 14 years, respectively.

The leaf anatomy of *Welwitschia* has been quite thoroughly studied by several authors [4, 5, 14, 15], but never with a distinction between the sexes. The unique morphology of *Welwitschia* leaves was used as a model in a paper concerning phenotyping of xerophyte plants [16]. A poor correlation between sex and growth rate in the Namib Desert was reported [8]. Long-term growth patterns of *Welwitschia* and a comprehensive bibliography concerning this species were published by Henschel and Seely [8] and van Jaarsveld and Pond [2]. Fossil Gnetales distributed in Gondwana date at least from the Mesozoic era in more humid habitats. The change of climate occurred after separation of Africa and South America and the development of the cold Benguela Current ca. 15 million years ago, which resulted in the aridification of the climate in the *Welwitschia* range [2, 17]. *Welwitschia* is the only member of Gnetales which was able to adapt to these new arid conditions [17], perhaps due to its ability to perform CAM, even though weakly. The CAM photosynthetic pathway evolved for the first time 250–300 million years ago and is observed in other related ancient gymnosperms such as cycads [18].

Research on the metabolism pattern and other ecophysiological studies of *W*. *mirabilis* have been conducted mainly in natural localities [14, 15, 19] and rarely under controlled conditions [15]. As there was a doubt whether *Welwitschia* is a CAM or solely a C3 plant, several authors have investigated the type of photosynthetic system of this species [14, 19–21]. These studies have confirmed that *Welwitschia* is a C3 plant with a facultative CAM cycle. Another approach to study differences in photosynthetic activity are vegetation indexes, which are calculated on a basis of canopy and leaf reflectance. The main studies in plant condition detection are based on the spectral wavelengths ranging from 400 to 2,500 nm. One of the most commonly used is the Normalized Difference Vegetation Index (NDVI), which has become a standard for vegetation condition assessment [22]. This type of measurement is usually used in the cultivated fields in connection with several biotic and abiotic conditions [23]. Rarely, it is applied in a research on single plants [24]. So far, no studies concerning photosynthetic activity have been conducted with regard to the sex of the plants studied.

Taking above into consideration the following hypotheses are proposed: the photosynthetic activity of male and female plants of *Welwitschia mirabilis* is connected with a plant gender, the differences in leaf micromorphology among male and female plants occur. An additional goal was to examine a usefulness of an analysis of a chlorophyll reflectance method in a study of leaf chlorophyll activity in a unique Gymnosperm species.

## Material and methods

### Materials

The specimens examined are given in a Table 1. They had been established from seed at the Adam Mickiewicz Botanical Garden in Poznań. The plants are kept the whole year in a greenhouse in specially designed rhizoboxes. They are planted in a mix of sand, SERAMIS®, fine grained gravel, peat and compost. It was developed through garden experiments by our employees as a most suitable for *Welwitschia* in cultivation. Plants are fertilized once a month with a fertilizer Florovit designated for succulents (N:P:K– 4:8:6 with microelements: B, Cu, Fe, Mn, Mo, Zn). The plants are watered once or twice a week depending on the season. The air humidity is around 30%, but every morning the plants are sprinkled with water to mimic the morning dew on the desert. Plants are cultivated with artificial light (sodium lamp 400 W, photoperiod 12 h/day). Intensity of radiation in the distance of 50 cm under the lamp was 21,3 klx (measured by lxmeter Sonopan L-20A). Plants were situated within a circle of radius 50 cm. Intensity of radiation at the circle border was 6–7 klx. Photosynthetic photon flux density (PPFD) was 285,5 μmol (photon) m^-2^s^-1^. The leaves of *Welwitschia* are growing constantly during its life, therefore there is no age difference in the leaf tissue between different plants, if measured at this same place from the trunk. In case of a plant which can survive up to 2000 years [2] age difference of a few years in adult flowering plants are negligible.

**Table 1:** List of specimens of *Welwitschia mirabilis* examined.

Specimen number	Plant gender	Plant age in years	AMU BG Collection number
F1	female	20	6998_4561
F2	female	15	6003_4606
F3	female	14	6004_4620
F4	female	15	6003_4624
F5	female	14	6004_4617
F6	female	20	6998_4563
M1	male	20	6998_4560
M2	male	14	6004_4610
M3	male	14	6004_4570
M4	male	15	6003_4623
M5	male	14	6004_4622

### Methods

#### Physiological study

The handheld photosynthetic system CI 340aa (CID Bioscience Inc., Camas, USA) was placed on the upper side of the leaf blade, in ca. one year old part of a leaf, around 20 cm from its base, at ca. one third of its width from the leaf edge. The system was used to measure net photosynthetic rate (*P*_N_), stomatal conductance (*g*_s_) and intercellular CO_2_ concentration (*C*_i_). Spearman’s rank correlation coefficients between photosynthetic characteristics of *P*_N_/*g*_s_ ratio was measured. To achieve comparable results of measurements, constant conditions in the leaf chamber were maintained: CO_2_ inflow concentration (400 μmol (CO_2_) mol^-1^), photosynthetic photon flux density (PPFD) 1000 μmol (photon) m^-2^s^-1^, chamber temperature 23°C and relative humidity 50±3%. Investigations were conducted during day hours from 8.00 am to 4.00 pm and during the night hours from 9.00 pm to 1.00 am. Gas exchange parameters were recorded every minute for the whole duration of each experiment (480 measurements during day hours and 240 measurements during the night hours). The tests were carried out under the above-mentioned controlled conditions on each of four individuals (F1, F2, M1, M2) during the day and two individuals at night (F2, M1) and were repeated for three nights and days. The number of individuals was restricted to four plants due to the technical reasons and the large amount of data collected for each individual. The data supporting our findings are included in S1–S6 Tables.

#### Vegetation index

MicaSense RedEdge-M multispectral camera was used to capture light reflected of the plant canopy in five spectral bands: blue, green, red, red-edge, near infrared. For the normalized difference vegetation index (NDVI) calculation following eq was used (1):

$$NDVI=\frac{NIR_{\left(840\right)}-Red_{\left(668\right)}}{NIR_{\left(840\right)}+Red_{\left(668\right)}}$$ (1)

**Fig 1:**
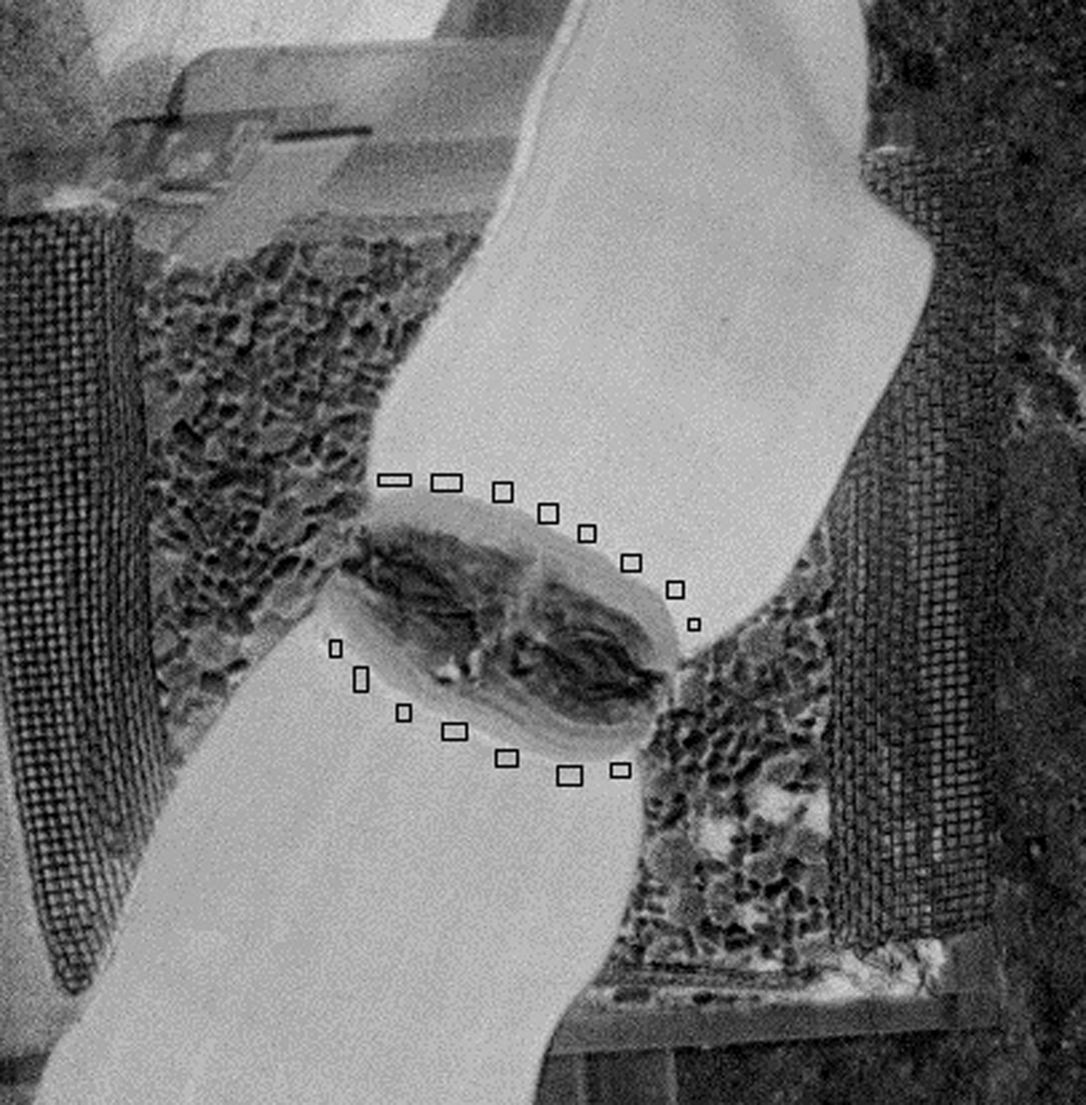
*Welwitschia mirabilis* leaf with marked area of capture light reflectance.

In order to calculate NDVI for each *Welwitschia mirabilis* plant (Table 1), the rectangular area of each leaf near stem was manually marked ten times and mean value for each rectangle and then for ten rectangles was calculated (Fig 1). For absolute reflectance estimation calibrated reflectance panels was used [25]. The data supporting our findings are included in S8 Table.

#### Anatomy

Samples of the epidermis were taken from one year old parts of leaves of two male and two female plants (M1, M2, F1, F2). The stomata were measured on the adaxial and abaxial part of the lamina with at least 3 samples taken, giving 12 measurements per each plant. Sections were mounted in a chloral hydrate solution and examined under the light microscope. The data supporting our findings are included in S7 Table.

#### Statistical analysis

Descriptive statistics were calculated (arithmetic average, standard errors, minimum and maximum). In order to determine statistical significance of average values of traits of the samples in question, the factor variance ANOVA *F*-statistics was used. The assumptions for the analysis of variance were tested. The data supporting our findings are included in S1–S3 and S7 Tables. In order to demonstrate the significance of differences between the tested parameters over time, Friedman ANOVA test was performed. The data supporting our findings are included in S1, S2, S4 and S5 Tables.

## Results

### Photosynthesis parameters

**Fig 2:**
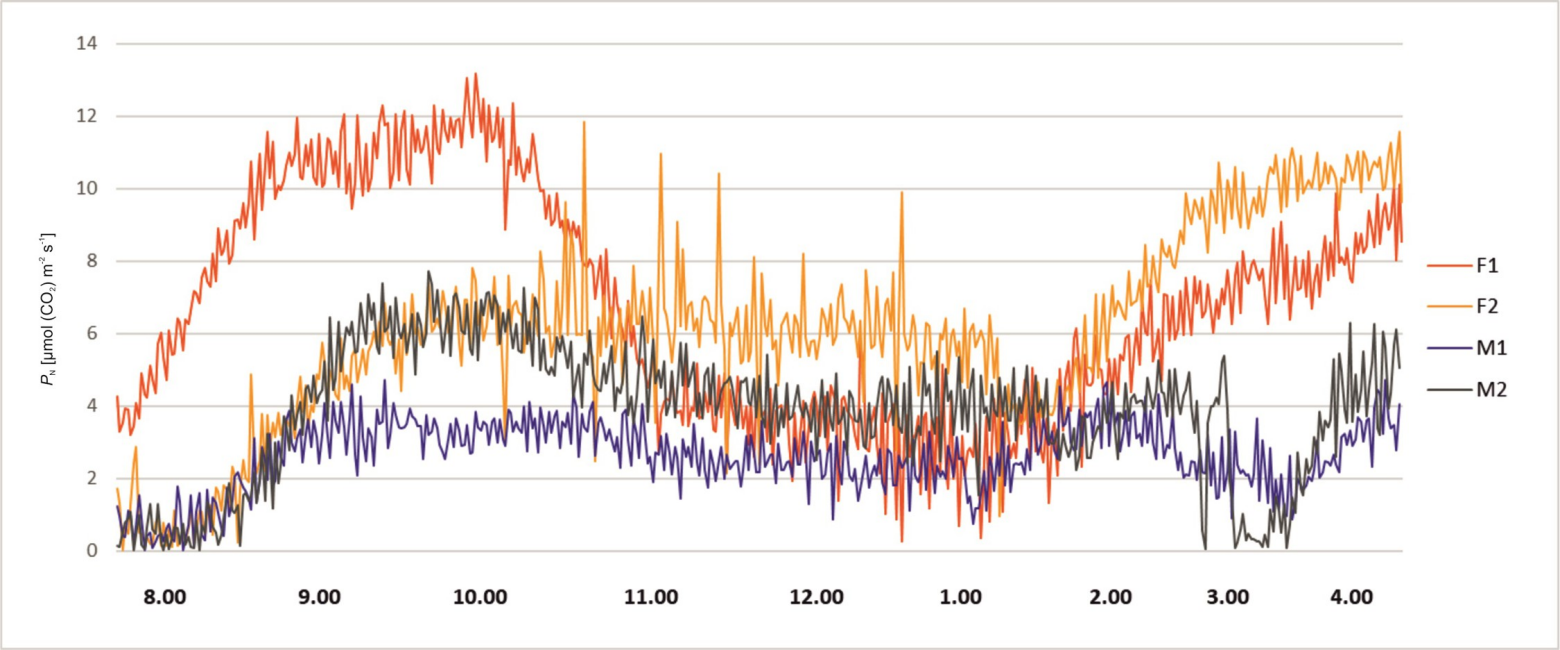
Changes of net photosynthesis rate (*P*_N_) measured for female (F1, F2) and male (M1, M2) *Welwitschia mirabilis* specimens, from 8 a.m. to 4 p.m.

Net photosynthesis rate of the studied plants revealed variability during the day hours. In all tested plants at 8.00 a.m. the activity was low, in the range from *P*_N_ ≈ 0–4 μmol (CO_2_) m^-2^ s^-1^. After one hour all plants exhibited higher activity of gas exchange. The female specimen F1 reached *P*_N_ between 9.00 a.m. and 10.00 a.m. up to 12 μmol (CO_2_) m^-2^ s^-1^, whereas F2 ca. 6 μmol (CO_2_) m^-2^ s^-1^. A similar tendency as for F1 was recorded for male M2, while a smaller increase was observed for M1. Female F2 and both male specimens revealed a more or less constant level up to 1.30 p.m., while a decrease of *P*_N_ was detected for F1, reaching as low as *P*_N_ ≈ 2 μmol (CO_2_) m^-2^ s^-1^ about 1.00 p.m. After 2 p.m. female plants started to show higher activity. Male specimens revealed a decrease to 1–2 μmol (CO_2_) m^-2^ s^-1^ at 3 p.m. About 4 p.m. there was an increase of activity of all tested plants (Fig 2).

**Fig 3:**
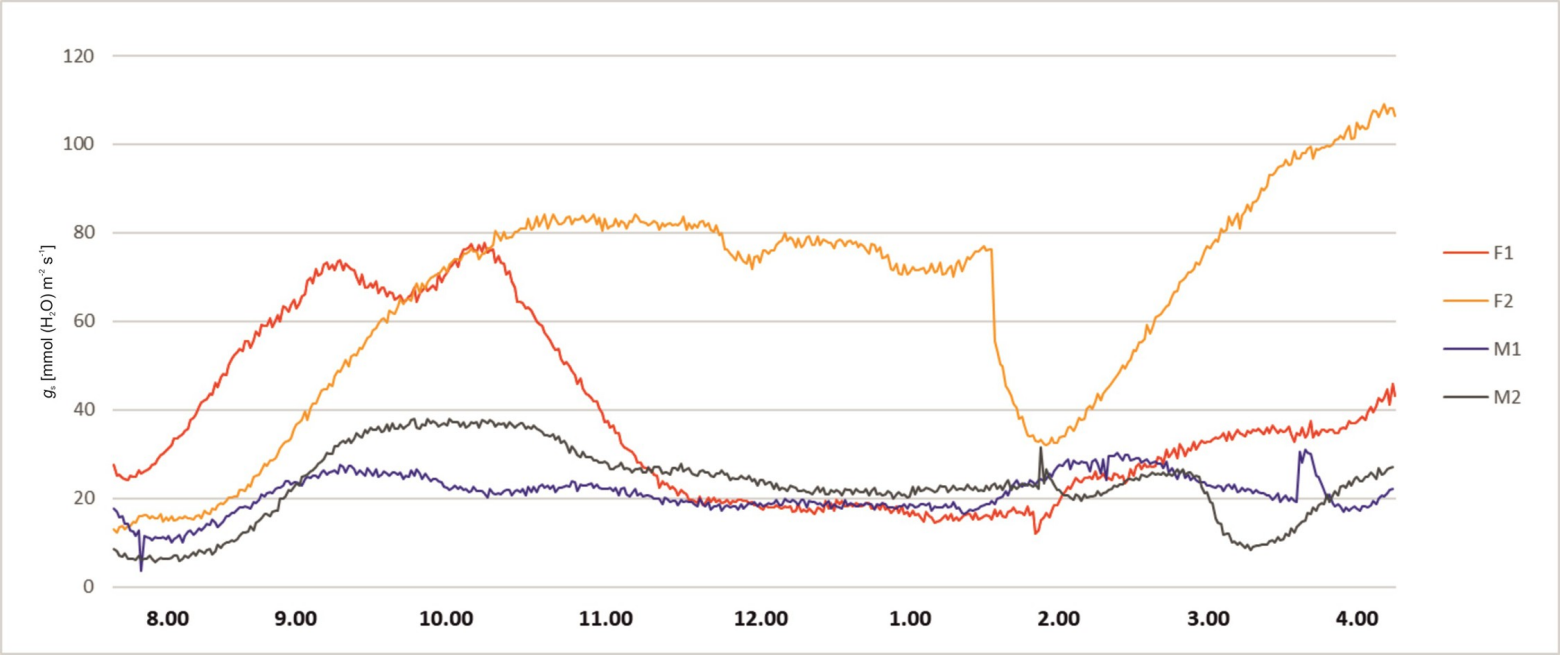
Changes of stomatal conductance (*g*_*s*_) measured for female (F1, F2) and male (M1, M2) *Welwitschia mirabilis* specimens, from 8 a.m. to 4 p.m.

**Fig 4:**
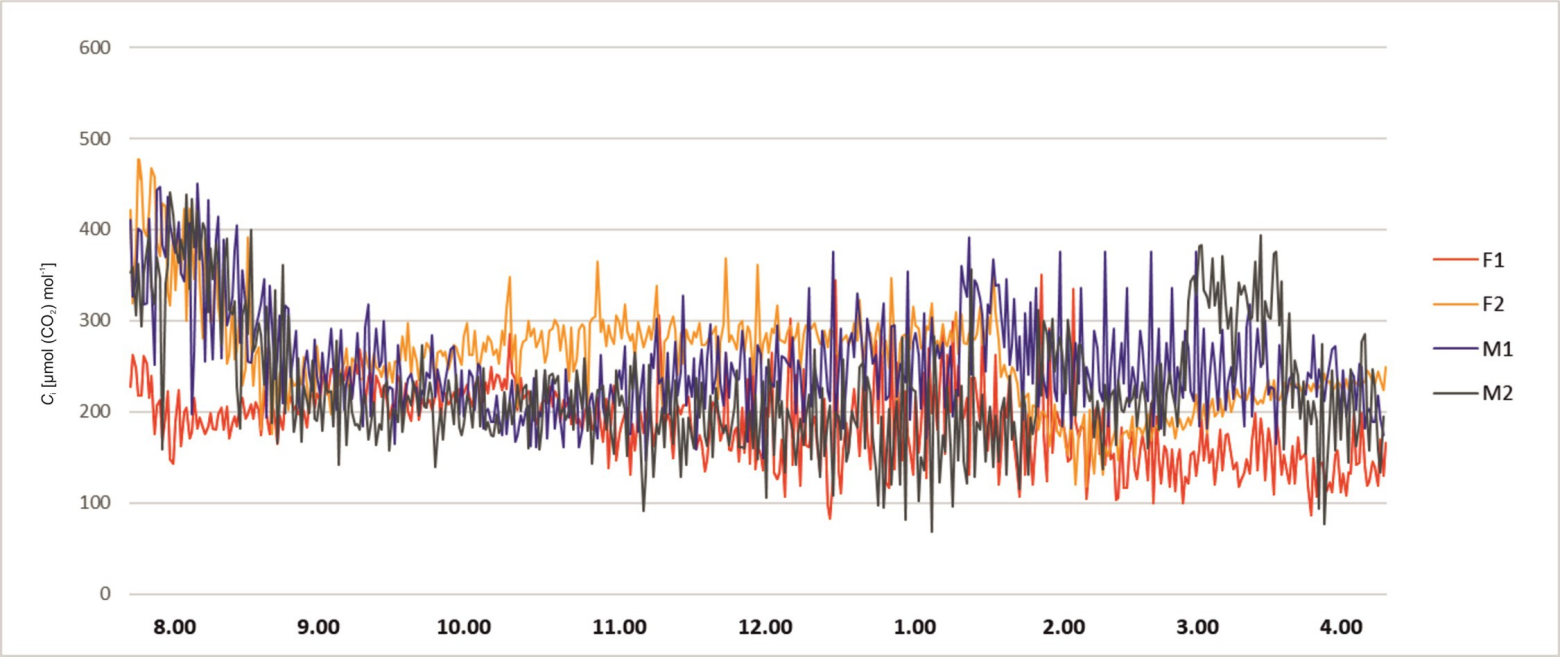
Changes of intercellular CO_2_ concentration *C*_i_ measured for female (F1, F2) and male (M1, M2) *Welwitschia mirabilis* specimens, from 8 a.m. to 4 p.m.

**Fig 5:**
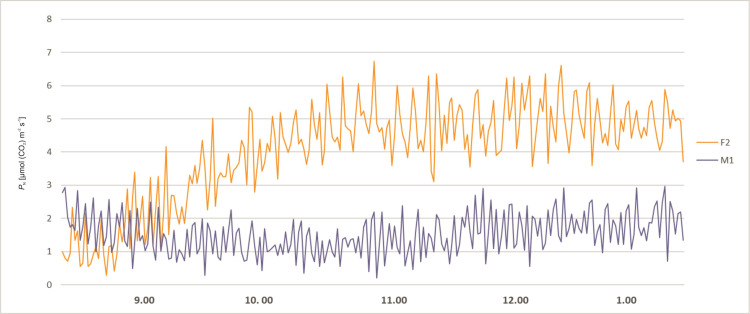
Changes of net photosynthesis rate (*P*_N_) measured for female (F2) and male (M1) *Welwitschia mirabilis* specimens from 9 p.m. to 1 a.m.

The stomatal conductance also varied during day hours. At 8 a.m. the *g*_s_ values of all plants were relatively low. About 9 a.m. the *g*_s_ values of all plants started to rise, more significantly in female specimens F1 and F2, reaching values between 65 and 80 mmol (H_2_O) m^-2^ s^-1^. The male specimens (M1 and M2) showed lower values of 20–40 mmol (H_2_O) m^-2^ s^-1^ and remained stable up to 3.00 p.m. The female plant F1 started to exhibit lower *g*_s_ values at the level 20 mmol (H_2_O) m^-2^ s^-1^ about 10.30 a.m. After noon the stomatal conductance of the F2 female decreased to 30 mmol (H_2_O) m^-2^ s^-1^ at 2 p.m. Afterwards, it started to grow until it reached the value of 100 mmol (H_2_O) m^-2^ s^-1^ at 4 p.m. In male specimens an increase of *g*_s_ was observed about 4 p.m. (Fig 3). Intercellular CO_2_ concentration (*C*_i_) of the studied plants revealed variability during the day hours. In all tested plants at 8.00 a.m. the activity was high, ranging from 200 to 450 μmol (CO_2_) mol^-1^. Afterwards, a decrease was recorded for all investigated specimens. Female F2 showed increased *C*_i_ in the midday hours and a sudden decrease between 1.00 and 2.00 p.m., and afterwards again an increase was noted. The time course of *C*_i_ for F2 is quite similar as for *P*_N_ and *g*_s_, but the decrease after 2.00 p.m. is relatively small. Plant M2 revealed a significant increase of *C*_i_ about 3.00 p.m., which does not reflect the *P*_N_ and *g*_s_ time course. F1 and M1 revealed the smallest variability during the day, which also reflects the tendencies of the other two photosynthesis parameters (Fig 4). Activity of a female specimen (F2) measured during the night hours, between 9 p.m. and 1 a.m., showed that the net photosynthesis rate *P*_N_ was low during the first hour and varied between 0.5 and 3.0 μmol (CO_2_) m^-2^ s^-1^. Later this activity (*P*_N_) increased significantly and stabilized within the range 4.0–6.8 μmol (CO_2_) m^-2^ s^-1^. In the case of a male specimen (M1) in the same period the activity stabilized and varied between 1 and 3 μmol (CO_2_) m^-2^ s^-1^ (Fig 5).

**Fig 6:**
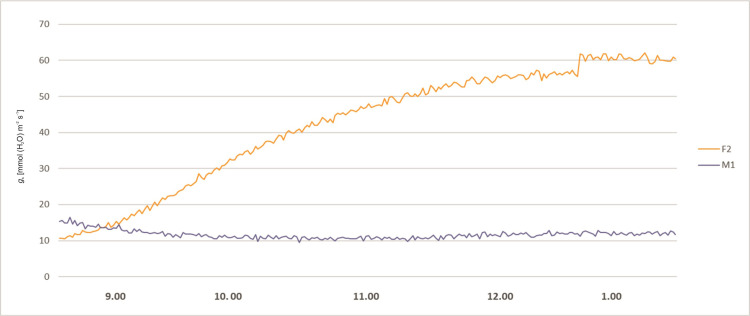
Stomatal conductance (*g*_*s*_) measured for female (F2) and male (M1) *Welwitschia mirabilis* specimens, from 9 p.m. to 1 a.m.

**Fig 7:**
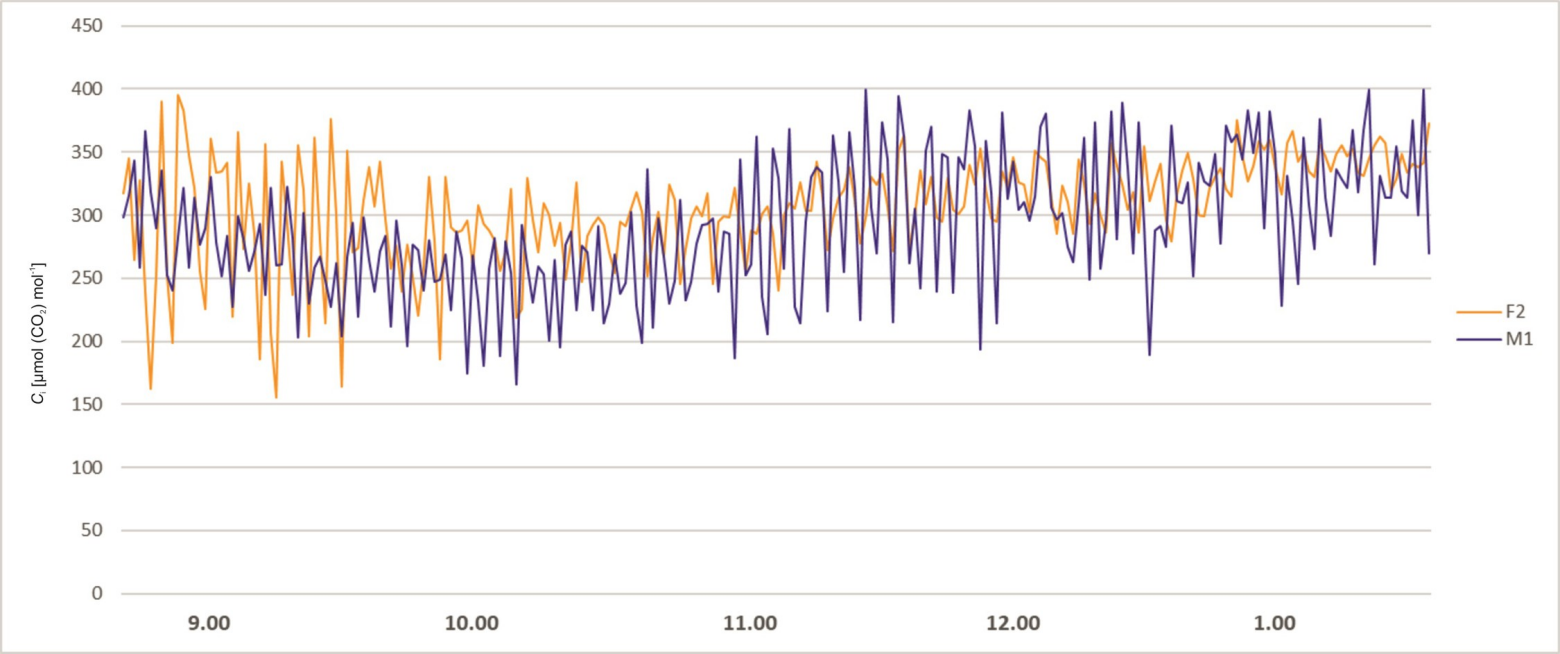
Changes of intercellular CO_2_ concentration *C*_i_ measured for female (F2) and male (M1) *Welwitschia mirabilis* specimens, from 9 p.m. to 1 a.m.

Plants examined during night hours (between 9 p.m. and 1 a.m.) showed a different pattern of stomatal conductance (*g*_s_) for female and male specimens, although the tendency was similar to *P*_N_. At 9 p.m. their activity was similar and comparably low, at a level of 10–20 mmol (H_2_O) m^-2^ s^-1^. Later the female plant F2 started to reveal successively higher values of *g*_s_ up to 60 mmol (H_2_O) m^-2^ s^-1^. The male specimen M1 exhibited a much lower level of *g*_s_ throughout the night hours (Fig 6). Intercellular CO_2_ concentration (*C*_i_) of a female and a male specimen (F2, M1) measured during the night hours revealed similar tendencies, with a slight decrease between 9 p.m. and 1 a.m., ranging from 150 μmol (CO_2_) mol^-1^ to 400 μmol (CO_2_) mol^-1^ (Fig 7).

**Fig 8:**
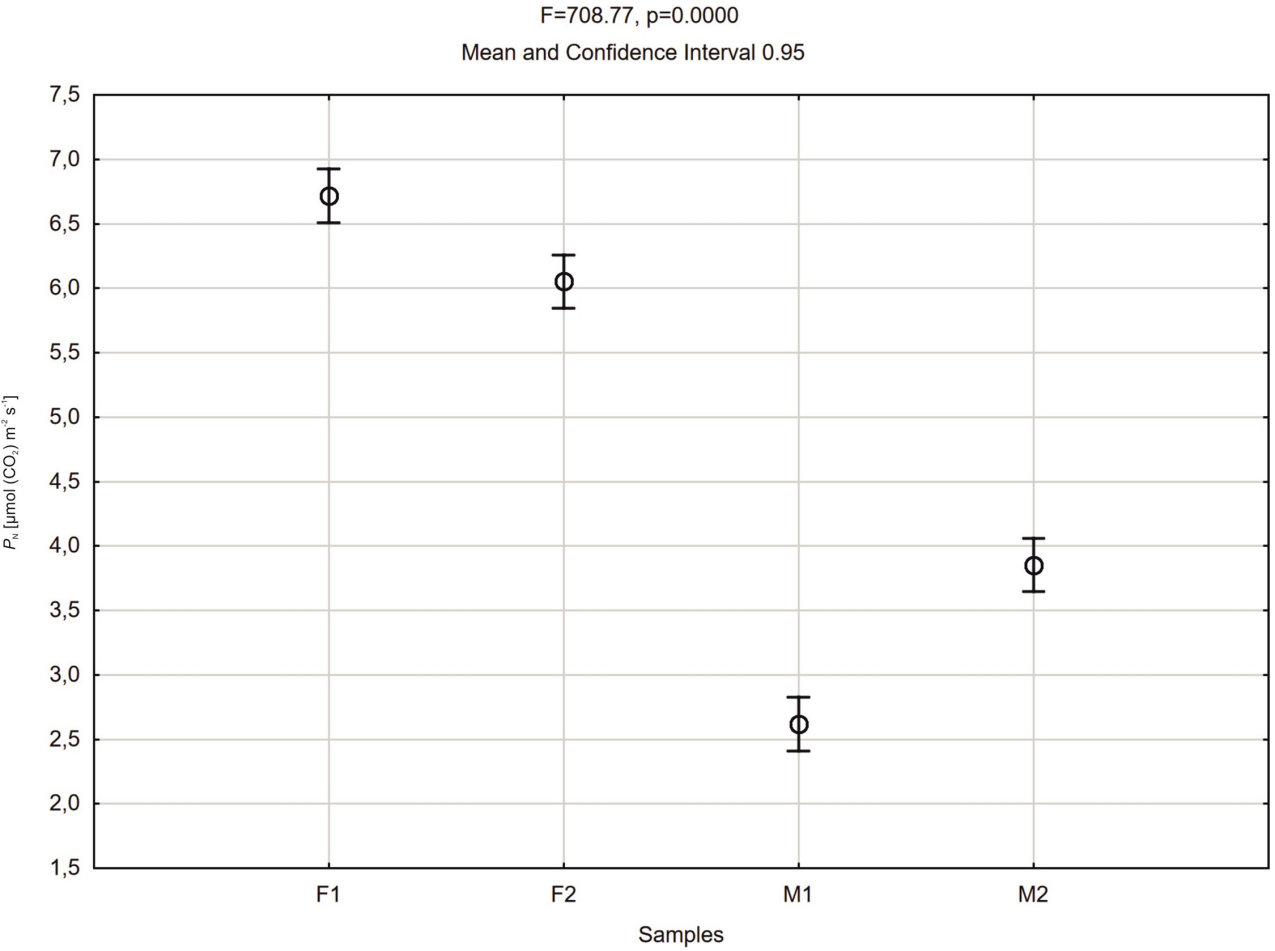
Mean values of net photosynthesis rate (*P*_N_) of *Welwitschia mirabilis* female (F1, F2) and male (M1, M2) plants from 8 a.m. to 4 p.m. Vertical bars present 0.95 confidence intervals.

**Fig 9:**
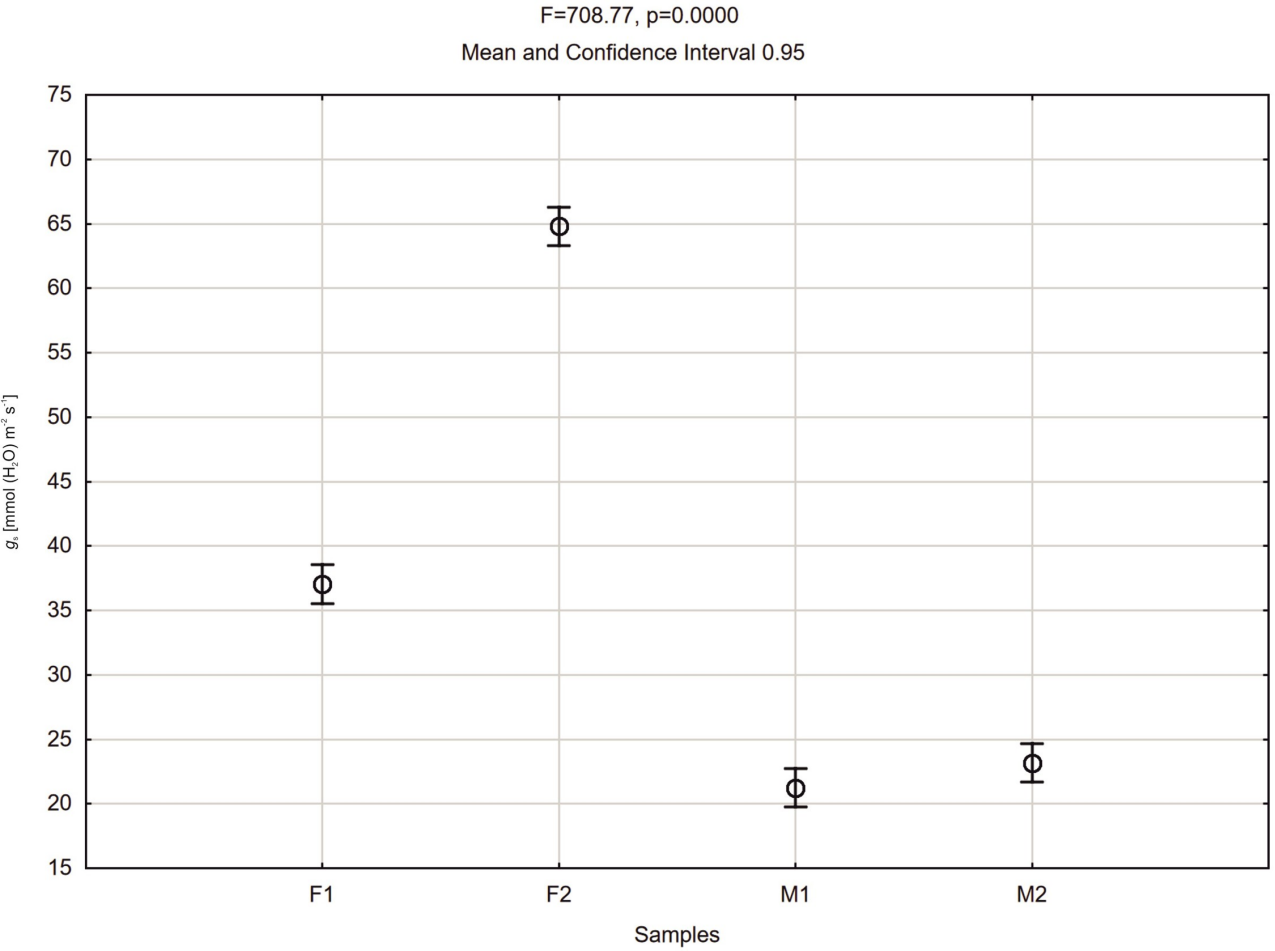
Mean values of stomatal conductance (*g*_*s*_) of *Welwitschia mirabilis* female (F1, F2) and male (M1, M2) plants from 8 a.m. to 4 p.m. Vertical bars present 0.95 confidence intervals.

Comparing average values of net photosynthesis rate (*P*_N_) of male and female plants of *Welwitschia mirabilis* for day hours, it can be noted that female plants revealed higher *P*_N_ values than the male ones (Fig 8). A similar tendency was recorded for *g*_s_ values (Fig 9).

**Fig 10:**
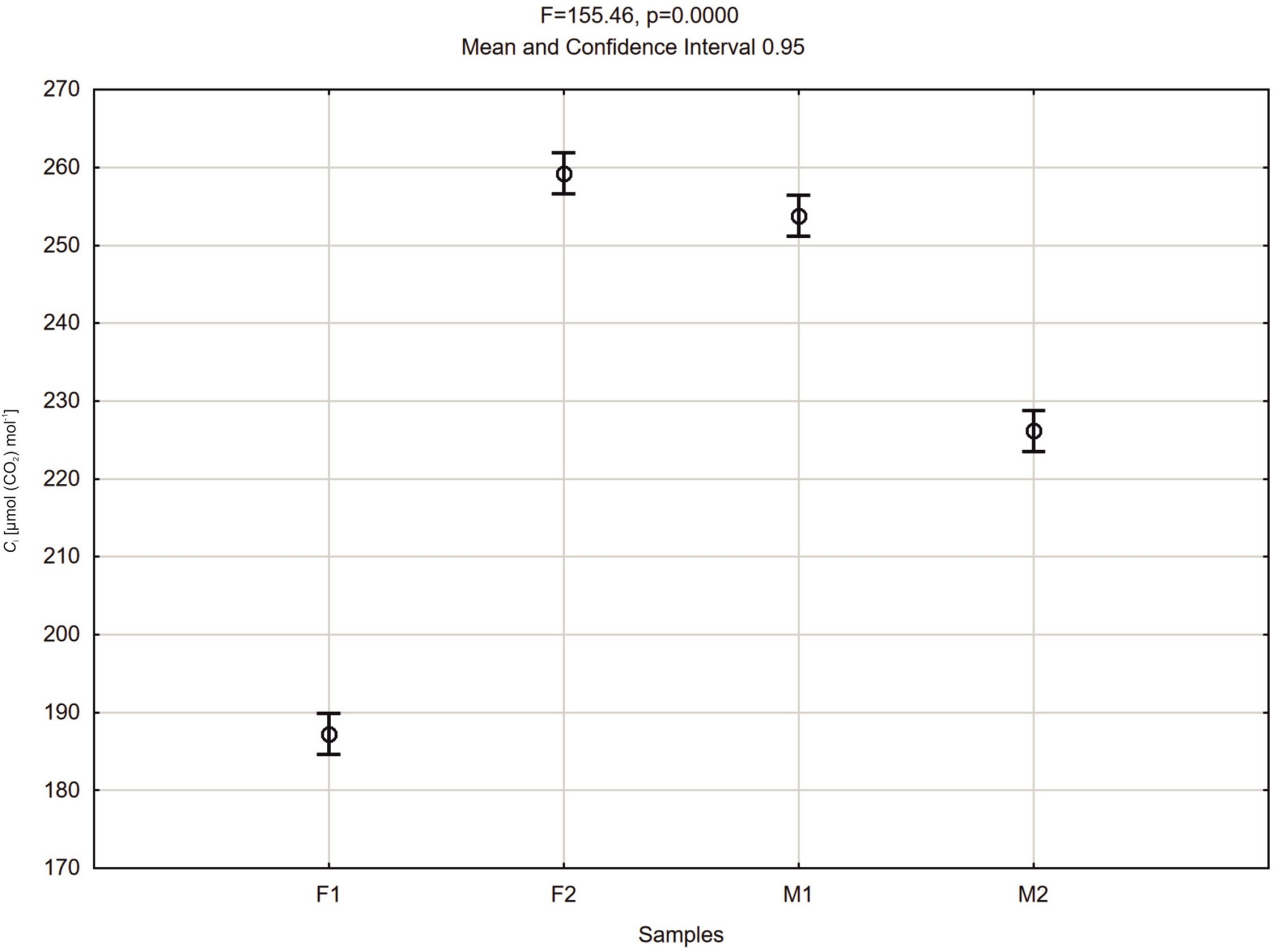
Mean values of intercellular CO_2_ concentration *C*_i_ of *Welwitschia mirabilis* female (F1, F2) and male (M1, M2) plants from 8 a.m. to 4 p.m. Vertical bars present 0.95 confidence intervals.

Mean values of *C*_i_ measured during day hours were higher for F2≈260 μmol (CO_2_) mol^-1^ and M1≈255 μmol (CO_2_) mol^-1^ specimens than *C*_i_ measured for M2≈228 μmol (CO_2_) mol^-1^) and F1≈190 μmol (CO_2_) mol^-1^ (Fig 10).

A very high correlation coefficient was found between P_N_ and *g*_s_ with a value 0,843 (p≤0.5) during the day, and lower during the night 0,647 (p≤0.5) for all specimens studied. Statistical analysis of plants over time has revealed that that there are significant differences between specimens tested (Friedman Chi-square ANOVA = 5387.653, p = 0.00000).

### Leaf micromorphology

**Fig 11:**
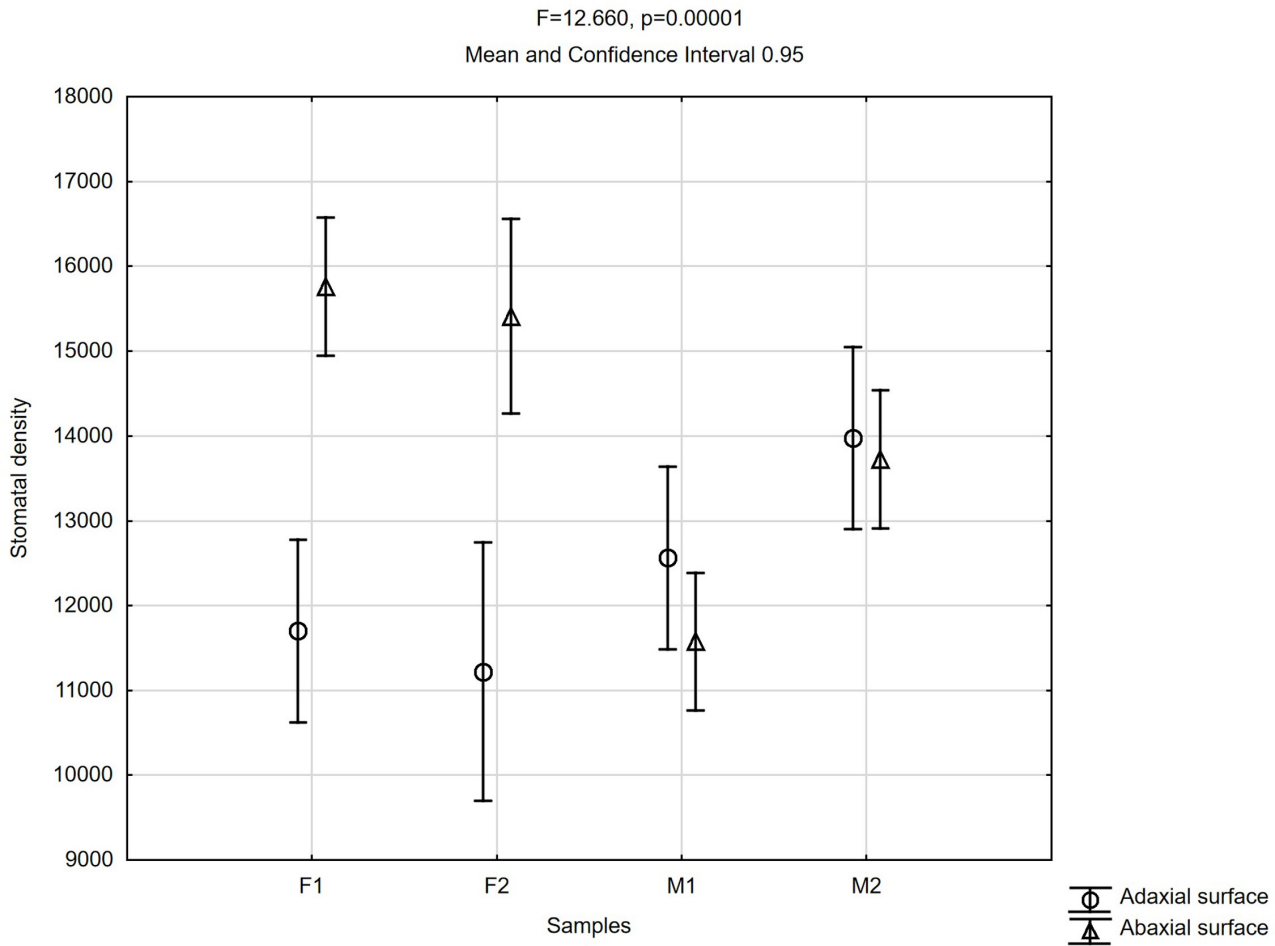
Mean values of stomatal density on 1 cm^2^ *Welwitschia mirabilis* on adaxial and abaxial leaf surface for female (F1, F2) and male (M1, M2) plants.

Stomatal density (number of stomata per cm^2^) was evaluated on both sides of the lamina. The density of stomata ranges from approximately 11 000 (F2) to 14 000 per 1 cm^2^ (M2) on the adaxial side and from 11 500 (M1) to ca. 16 000 per 1 cm^2^ (F1) on the abaxial surface of the lamina (Fig 11).

At the abaxial surface of the lamina the highest stomatal density was recorded in F1 and F2, the lowest in M1 and M2 male plants. The adaxial surface of the lamina exhibited generally lower stomatal density. Density of stomata, on both adaxial and abaxial surfaces of lamina (traits 1, 2, respectively), showed significant variability within examined species. Statistical values of *F* were statistically significant at the level p < 0.001 for these traits.

## Vegetation index

**Fig 12:**
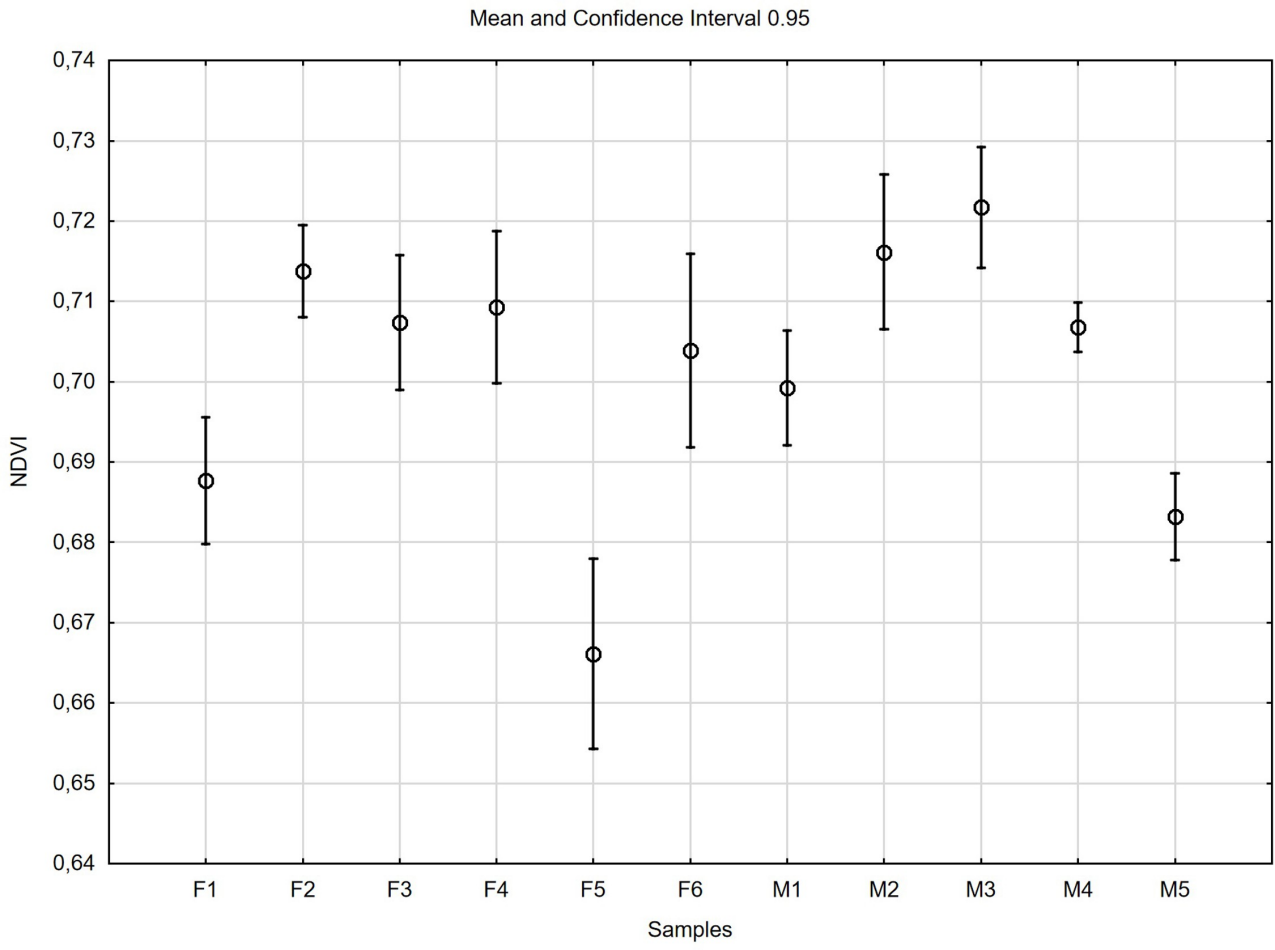
Normalized difference vegetation index (NDVI) in female (F) and male (M) *Welwitschia mirabilis* specimens. Midpoints represents mean values, vertical bars represent 0.95 confidence interval.

The study using the normalized difference vegetation index (NDVI), showed that the tested female and male specimens differed slightly in terms of the mesophyll condition. The index had values from 0.665 for 10F to 0.720 for M3 (Fig 12).

## Discussion

Our study focused on differences in anatomy of leaves and photosynthetic activity between male and female plants of *Welwitschia* in controlled conditions. Previous study of a CO_2_ intake of *Welwitschia* during the day or day and night [14] were not giving the number of individuals at all, or the number was restricted to two individuals [20]. Therefore our study is the first showing differences among more than two *Welwitschia* plants.

We detected significant differences in the mean values of stomatal density on abaxial and adaxial leaf surfaces, which are especially visible for female specimens. In female plants abaxial density is distinctly higher than adaxial density of stomata. This ratio is less clear but opposite in male specimens (Fig 11). Such a difference in the number of stomata on the two leaf surfaces is not uncommon in plants [e.g. 26, 27, 28]. In previous investigations, the number of stomata on the two surfaces of a *Welwitschia* leaf was reported as equal, with as many as 22 200 stoma/cm^2^ [15, 29]. As no sex of the plants studied was given, solely male specimens might have been studied.

Our studies have confirmed that *Welwitschia* exhibits both C3 and CAM photosynthetic pathways as described by other authors [19–21]. The stomata were open both during the day and during the night. Our observations of the diurnal photosynthetic activity *P*_N_ and *g*_*s*_ revealed two peaks in the morning and in the afternoon with a steep decrease in the middle of the day, as observed before by von Willert et al. [21]. However, in our observations there was a difference in the examined parameters (*P*_N_ and *g*_*s*_) between sexes. The two female specimens showed higher values during the day and night hours than the two male plants. The night CO_2_ intake was relatively low in both sexes in comparison to their day activity, what is confirmed by the *P*_N_/*g*_*s*_ correlation coefficients, but the female plant was more active than the male one.

Winter et al. [30] in a study of photosynthetic patterns in *Agave angustifolia* Haw. discovered that a day/night pattern of CO_2_ exchange was highly conserved under a range of environmental conditions and was insensitive to intensive watering. In potted *Welwitschia* intercellular CO_2_ concentration (*C*_i_) was also approximately this same during day and night hours (Figs 4 and 7).

Data presented by Winter and Schramm [20] suggested a very low potential for CAM in *Welwitschia*. Net dark CO_2_ fixation was barely observable, yet reduced rates of net CO_2_ loss for extended periods of the night, obtained under constant temperature conditions, clearly indicate a higher capacity for dark CO_2_ fixation in *Welwitschia* than in regular C3 leaves. In our study we demonstrated that this CO_2_ fixation is also sex dependent, because net photosynthetic rate (*P*_N_), stomatal conductance (*g*_s_) was higher in female plants (Figs 5 and 6).

This last observation suggests different physiological adaptations between two sexes of *Welwitschia*. The observed plants were after hormonal determination of the branches and in a final step of their life history according to Martens [31], when indefinite persistence of vegetative activity prevail, with continuous appearance of new buds. Therefore the plants were exhibiting physiological traits characteristic for their sex. In our collection, male specimens have developed flowers as early in their life time, as five years old, while female have to be at least 14 years old. Similar situation was observed in another botanic garden [32]. *Welwitschia* has a 9–10 month reproductive cycle, from bud initiation until seed dispersal [7]. Even though male plants form more branches and strobili, the female cones are longer and wider than the male ones [7]. The flowering period of *Welwitschia* lasts for about 8–9 weeks, and after that the male inflorescences are discarded. The female plants have to support developing seeds for another five to six month after the flowering period [7]. Therefore the allocation of resources to reproduction process is probably higher in female plants and in an attempt to balance this energetic demand, they exhibit a higher photosynthetic activity than male ones.

The obtain results of hyperspectral studies expressed by the normalized vegetation index (NDVI) confirmed the usefulness of this tool for assessing the condition of individual plants [25]. The studies of energy balance in *Welwitschia mirabilis* leaves were conducted by Schulze et al. [33] with spectral properties, reflectivity, transmissivity, and absorptivity in the wave length range from 400 to 1,350 nm on cut material in the laboratory. Our studies were conducted on living plants in full sun, in summer, mimicking natural environment. Schulze et al. [33] has established high reflectivity of the *Welwitschia* leaves in the near infrared, measuring spectral properties of leaves in the wavelength range of 400 to 1,350 nm. On the base of this results we calculated NDVI, which is approximately 0.5. In our studies of the leaves of *W*. *mirabilis* NDVI ranged from od 0.665 to 0.720 and showed slight differences among genders. Authors of another work [34] assessed that in *Welwitschia* there are no significant spectral difference between genders and along leaves.

## Conclusions

The photosynthetic flexibility in *Welwitschia mirabilis* in terms of the contributions to carbon gain of CO_2_ uptake in the dark and light conditions was detected. In the light, differences between male and female specimens were found in net photosynthesis rate (*P*_N_) *P*_N_ ≈ 2.5–3.5 and *P*_N_ ≈ 6.0–6.5 respectively. In the dark, female specimen showed higher activity due to net photosynthesis rate (*P*_N_), than male one. NDVI calculated for female and male specimens of *W*. *mirabilis* pointed to good conditions of mesophyll tissue in all plants. Moreover, significant differences of stomatal density on abaxial and adaxial leaf surface of female plants, (≈16000 on 1cm^2^ and ≈11000 on 1 cm^2^) respectively were observed. Further studies concerning photosynthetic differences among dioecious plants are need.

## Supporting information

S1 Table Net photosynthesis rate (*P*_N_) measurements for female (F1, F2) and male (M1, M2) *Welwitschia mirabilis* specimens, from 8 a.m. to 4 p.m. (PDF)

S2 Table Stomatal conductance (*g*_*s*_) measurements for female (F1, F2) and male (M1, M2) *Welwitschia mirabilis* specimens, from 8 a.m. to 4 p.m. (PDF)

S3 Table Measurements of intercellular CO_2_ concentration *C*_i_ measured for female (F1, F2) and male (M1, M2) *Welwitschia mirabilis* specimens, from 8 a.m. to 4 p.m. (PDF)

S4 Table Net photosynthesis rate (*P*_N_) measurements for female (F2) and male (M1) *Welwitschia mirabilis* specimens from 9 p.m. to 1 a.m. (PDF)

S5 Table Stomatal conductance (*g*_*s*_) measurements for female (F2) and male (M1) *Welwitschia mirabilis* specimens, from 9 p.m. to 1 a.m. (PDF)

S6 Table Intercellular CO_2_ concentration *C*_i_ measurements for female (F2) and male (M1) *Welwitschia mirabilis* specimens, from 9 p.m. to 1 a.m. (PDF)

S7 Table Values of stomatal density on 1 cm^2^ *Welwitschia mirabilis* on adaxial and abaxial leaf surface for female (F1, F2) and male (M1, M2) plants. (PDF)

S8 Table Measurements of vegetation index (NDVI) in female (F) and male (M) *Welwitschia mirabilis* specimens. (PDF)
