# Supplementary material for: Does a gender of Welwitschia mirabilis plants influence their photosynthetic activity?
Source: PLoS One. 2023 Sep 8;18(9):e0291122. doi: 10.1371/journal.pone.0291122 (PMC10490862; doi:10.1371/journal.pone.0291122)
Supplement: S1 Table — (PDF) [file pone.0291122.s001.pdf]

Measurement number	Specimen number	Parameter P_N
1	F1	4,25
2	F1	3,28
3	F1	3,55
4	F1	3,94
5	F1	3,9
6	F1	3,2
7	F1	3,46
8	F1	4,08
9	F1	3,67
10	F1	4,91
11	F1	4,32
12	F1	4,24
13	F1	5,1
14	F1	4,74
15	F1	4,34
16	F1	5,73
17	F1	6,03
18	F1	5,35
19	F1	4,73
20	F1	6,07
21	F1	5,42
22	F1	5,45
23	F1	6,41
24	F1	6,29
25	F1	5,53
26	F1	6,37
27	F1	6,27
28	F1	6,64
29	F1	7,17
30	F1	7,12
31	F1	6,83
32	F1	7,53
33	F1	7,82
34	F1	7,44
35	F1	7,3
36	F1	8,21
37	F1	7,45
38	F1	8,9
39	F1	8,16
40	F1	8,3
41	F1	8,83
42	F1	7,94
43	F1	8,18

44	F1	9,1
45	F1	9,14
46	F1	8,91
47	F1	9,58
48	F1	8,93
49	F1	9,62
50	F1	10,74
51	F1	8,59
52	F1	9,74
53	F1	10,96
54	F1	9,41
55	F1	10,65
56	F1	11,55
57	F1	10,3
58	F1	11,28
59	F1	9,72
60	F1	10,09
61	F1	9,96
62	F1	10,23
63	F1	10,63
64	F1	11
65	F1	10,62
66	F1	10,96
67	F1	11,95
68	F1	10,33
69	F1	10,27
70	F1	11,2
71	F1	10,61
72	F1	11,36
73	F1	10,32
74	F1	10,1
75	F1	11,49
76	F1	10,05
77	F1	10,13
78	F1	11,38
79	F1	11,29
80	F1	10,41
81	F1	11,01
82	F1	10,05
83	F1	11,57
84	F1	12,04
85	F1	9,88
86	F1	10,7
87	F1	9,43
88	F1	10,18

89	F1	12,01
90	F1	10,46
91	F1	9,82
92	F1	11,22
93	F1	9,94
94	F1	10,33
95	F1	11,52
96	F1	10,83
97	F1	11,81
98	F1	12,29
99	F1	11,75
100	F1	11,8
101	F1	10,01
102	F1	10,48
103	F1	12,04
104	F1	10,22
105	F1	11,58
106	F1	12,14
107	F1	10,52
108	F1	10,11
109	F1	12,01
110	F1	11,02
111	F1	11,62
112	F1	10,99
113	F1	11,11
114	F1	11,73
115	F1	11,15
116	F1	10,14
117	F1	12,3
118	F1	11,11
119	F1	10,96
120	F1	12,16
121	F1	11,62
122	F1	11,29
123	F1	11,96
124	F1	11,42
125	F1	11,86
126	F1	11,93
127	F1	11,14
128	F1	12,22
129	F1	13,04
130	F1	11,9
131	F1	11,41
132	F1	13,16
133	F1	12,55

134	F1	11,55
135	F1	12,47
136	F1	10,76
137	F1	12,3
138	F1	11,29
139	F1	11,4
140	F1	12,23
141	F1	11,13
142	F1	11,92
143	F1	8,87
144	F1	10,79
145	F1	10,65
146	F1	12,34
147	F1	10,38
148	F1	11,13
149	F1	10,48
150	F1	10,19
151	F1	10,82
152	F1	10,43
153	F1	11,49
154	F1	11,04
155	F1	10,26
156	F1	9,92
157	F1	9,96
158	F1	9,18
159	F1	9,82
160	F1	8,99
161	F1	9,13
162	F1	9,86
163	F1	8,94
164	F1	9,11
165	F1	8,46
166	F1	9,15
167	F1	8,59
168	F1	9,08
169	F1	8,65
170	F1	8,95
171	F1	8,02
172	F1	7,91
173	F1	7,84
174	F1	8,06
175	F1	7,88
176	F1	6,97
177	F1	7,7
178	F1	8,14

179	F1	6,61
180	F1	8,33
181	F1	6,8
182	F1	5,87
183	F1	7,54
184	F1	6,85
185	F1	6,33
186	F1	6,07
187	F1	5,53
188	F1	6,89
189	F1	5,66
190	F1	6,19
191	F1	5,33
192	F1	5,12
193	F1	5,3
194	F1	4,97
195	F1	4,5
196	F1	4,79
197	F1	5,3
198	F1	4,82
199	F1	2,71
200	F1	3,77
201	F1	4,11
202	F1	4,19
203	F1	4,15
204	F1	4,83
205	F1	3,97
206	F1	3,8
207	F1	3,87
208	F1	3,49
209	F1	5,51
210	F1	3,45
211	F1	3,95
212	F1	3,86
213	F1	5,17
214	F1	3,66
215	F1	3,71
216	F1	4,38
217	F1	3,83
218	F1	4,59
219	F1	3,35
220	F1	3,3
221	F1	4,83
222	F1	3,7
223	F1	4,9

224	F1	2,49
225	F1	4,44
226	F1	3,96
227	F1	3,48
228	F1	4,8
229	F1	4,04
230	F1	3,39
231	F1	4
232	F1	2,94
233	F1	4,07
234	F1	2,59
235	F1	4,09
236	F1	3,69
237	F1	3,73
238	F1	3,87
239	F1	3,03
240	F1	4,48
241	F1	2,38
242	F1	3,87
243	F1	3,96
244	F1	4,02
245	F1	3,45
246	F1	4,12
247	F1	3
248	F1	1,94
249	F1	3,81
250	F1	2,46
251	F1	3,21
252	F1	4,15
253	F1	2,45
254	F1	3,45
255	F1	4,01
256	F1	4,38
257	F1	4,04
258	F1	3,16
259	F1	3,84
260	F1	4,26
261	F1	2,78
262	F1	4,57
263	F1	4,36
264	F1	4,07
265	F1	1,39
266	F1	4,18
267	F1	4,27
268	F1	3,86

269	F1	3,78
270	F1	3,63
271	F1	3,2
272	F1	2,82
273	F1	5,8
274	F1	4,99
275	F1	2,84
276	F1	2,74
277	F1	3,55
278	F1	3,97
279	F1	2,97
280	F1	2,15
281	F1	4,34
282	F1	1,03
283	F1	3,56
284	F1	3,83
285	F1	4,04
286	F1	0,88
287	F1	3,66
288	F1	0,26
289	F1	3,11
290	F1	3,75
291	F1	2,6
292	F1	3,16
293	F1	3,13
294	F1	3,73
295	F1	1,58
296	F1	3,69
297	F1	3,82
298	F1	1,17
299	F1	3,64
300	F1	2,03
301	F1	3,7
302	F1	2,21
303	F1	5,15
304	F1	2,33
305	F1	1,73
306	F1	4,28
307	F1	1,99
308	F1	2,81
309	F1	0,68
310	F1	3,16
311	F1	2,08
312	F1	2,74
313	F1	2,83

314	F1	2,61
315	F1	3,51
316	F1	2,2
317	F1	0,36
318	F1	2,41
319	F1	3,71
320	F1	0,81
321	F1	2,89
322	F1	4,19
323	F1	2,55
324	F1	4,45
325	F1	1,07
326	F1	3,8
327	F1	3,19
328	F1	2,58
329	F1	2,96
330	F1	2,57
331	F1	4,02
332	F1	3,24
333	F1	4,71
334	F1	3,8
335	F1	2,53
336	F1	5,04
337	F1	2,76
338	F1	4,41
339	F1	2,74
340	F1	2,44
341	F1	3,65
342	F1	1,32
343	F1	3,26
344	F1	3,55
345	F1	2,07
346	F1	5,02
347	F1	3,32
348	F1	4,72
349	F1	5,22
350	F1	3,87
351	F1	5,88
352	F1	6,14
353	F1	4,93
354	F1	2,32
355	F1	5,42
356	F1	4,59
357	F1	4,58
358	F1	4,65

359	F1	5,96
360	F1	5,05
361	F1	5
362	F1	4,36
363	F1	4,32
364	F1	5,3
365	F1	4,27
366	F1	5,28
367	F1	4,32
368	F1	5,41
369	F1	5,25
370	F1	5,86
371	F1	5,96
372	F1	4,39
373	F1	5,56
374	F1	6,15
375	F1	5,13
376	F1	6,9
377	F1	6,04
378	F1	5,96
379	F1	7,46
380	F1	5,22
381	F1	5,68
382	F1	6,11
383	F1	4,68
384	F1	7,07
385	F1	7,05
386	F1	5,8
387	F1	7,03
388	F1	5,85
389	F1	6,15
390	F1	7,18
391	F1	5,95
392	F1	7,52
393	F1	6,57
394	F1	5,96
395	F1	7,55
396	F1	6,84
397	F1	7,44
398	F1	6,44
399	F1	6,62
400	F1	7,35
401	F1	6,61
402	F1	6,02
403	F1	6,93

404	F1	6,4
405	F1	7,22
406	F1	6,82
407	F1	7,76
408	F1	7,16
409	F1	6,26
410	F1	7,61
411	F1	7,95
412	F1	6,58
413	F1	8,25
414	F1	7,57
415	F1	7,38
416	F1	7,89
417	F1	8,01
418	F1	7,7
419	F1	7,48
420	F1	7,77
421	F1	7,07
422	F1	6,25
423	F1	7,47
424	F1	8,89
425	F1	6,84
426	F1	8,27
427	F1	9,08
428	F1	6,95
429	F1	8,44
430	F1	6,38
431	F1	7,1
432	F1	8,12
433	F1	7,21
434	F1	7,27
435	F1	8,03
436	F1	7,01
437	F1	8,27
438	F1	6,7
439	F1	7,37
440	F1	7,91
441	F1	7,02
442	F1	8,18
443	F1	8,7
444	F1	7,46
445	F1	8,51
446	F1	7,6
447	F1	9,88
448	F1	7,96

449	F1	8,12
450	F1	7,91
451	F1	8,35
452	F1	7,54
453	F1	7,42
454	F1	8,79
455	F1	8,19
456	F1	8,75
457	F1	8,22
458	F1	8,42
459	F1	9,39
460	F1	8,83
461	F1	8,38
462	F1	9,84
463	F1	8,46
464	F1	9,34
465	F1	9,6
466	F1	8,86
467	F1	9,11
468	F1	10,12
469	F1	8,02
470	F1	10,12
471	F1	8,52
472	F2	1,72
473	F2	1,34
474	F2	0,01
475	F2	0,68
476	F2	0,48
477	F2	1,26
478	F2	2,42
479	F2	2,88
480	F2	0,52
481	F2	0,47
482	F2	0,24
483	F2	0,4
484	F2	0,19
485	F2	0,16
486	F2	0,79
487	F2	0,94
488	F2	0,13
489	F2	0,78
490	F2	0,14
491	F2	0,59
492	F2	0,12
493	F2	1,1

494	F2	0,14
495	F2	0,2
496	F2	0,64
497	F2	0,23
498	F2	0,65
499	F2	1,25
500	F2	0,17
501	F2	0,53
502	F2	1,12
503	F2	1,09
504	F2	0,45
505	F2	1,01
506	F2	1,27
507	F2	0,44
508	F2	1,74
509	F2	1,63
510	F2	1,11
511	F2	1,82
512	F2	1,68
513	F2	1,17
514	F2	2,32
515	F2	2,08
516	F2	0,23
517	F2	1,7
518	F2	2,5
519	F2	2,02
520	F2	1,89
521	F2	4,87
522	F2	2,5
523	F2	2,15
524	F2	2,5
525	F2	3,78
526	F2	2,19
527	F2	3,75
528	F2	2,85
529	F2	3,53
530	F2	3,15
531	F2	3,81
532	F2	3,19
533	F2	3,54
534	F2	3,66
535	F2	3,46
536	F2	3,72
537	F2	3,11
538	F2	4,03

539	F2	4,8
540	F2	3,85
541	F2	4,58
542	F2	3,38
543	F2	4,03
544	F2	3,85
545	F2	4,87
546	F2	4,74
547	F2	5,74
548	F2	5,23
549	F2	3,99
550	F2	5
551	F2	4,69
552	F2	4,32
553	F2	4,16
554	F2	5,88
555	F2	4,48
556	F2	4,85
557	F2	4,4
558	F2	5,38
559	F2	5,58
560	F2	4,67
561	F2	5,15
562	F2	5,56
563	F2	4,86
564	F2	5,5
565	F2	5,71
566	F2	6,05
567	F2	5,65
568	F2	6,32
569	F2	5,66
570	F2	6,84
571	F2	5,84
572	F2	5,7
573	F2	5,9
574	F2	4,91
575	F2	5,85
576	F2	4,42
577	F2	6,84
578	F2	5,41
579	F2	6,11
580	F2	7,09
581	F2	5,98
582	F2	6,17
583	F2	5,7

584	F2	5,68
585	F2	6,38
586	F2	7,39
587	F2	6,05
588	F2	6,15
589	F2	6,42
590	F2	6,17
591	F2	7,17
592	F2	6,81
593	F2	6,02
594	F2	6,11
595	F2	6,87
596	F2	7,29
597	F2	5,75
598	F2	5,12
599	F2	6,93
600	F2	6,93
601	F2	5,57
602	F2	7,82
603	F2	7,43
604	F2	6,23
605	F2	6,72
606	F2	5,8
607	F2	6,19
608	F2	6,94
609	F2	6,73
610	F2	7,56
611	F2	5,68
612	F2	6,74
613	F2	4,75
614	F2	2,96
615	F2	7,61
616	F2	6,17
617	F2	6,75
618	F2	6,89
619	F2	6,6
620	F2	6,5
621	F2	5,49
622	F2	6,33
623	F2	5,8
624	F2	7,07
625	F2	6,51
626	F2	5,93
627	F2	8,25
628	F2	7,48

629	F2	6,01
630	F2	6,2
631	F2	5,36
632	F2	5,82
633	F2	7,44
634	F2	5,88
635	F2	6,74
636	F2	9,63
637	F2	5,96
638	F2	8,8
639	F2	8,4
640	F2	5,96
641	F2	5,98
642	F2	5,95
643	F2	11,83
644	F2	5,87
645	F2	5,52
646	F2	5,36
647	F2	2,48
648	F2	6,43
649	F2	5,38
650	F2	7,06
651	F2	5,63
652	F2	6,4
653	F2	6,72
654	F2	5,19
655	F2	5,81
656	F2	6,7
657	F2	4,61
658	F2	6,22
659	F2	6,85
660	F2	6,08
661	F2	7,88
662	F2	6,26
663	F2	6,25
664	F2	7,18
665	F2	6,67
666	F2	5,75
667	F2	7,25
668	F2	5,78
669	F2	3,53
670	F2	7,8
671	F2	10,95
672	F2	6,68
673	F2	6,51

674	F2	5,83
675	F2	5,21
676	F2	6,17
677	F2	9,08
678	F2	6,21
679	F2	8,31
680	F2	6,1
681	F2	6,74
682	F2	6,87
683	F2	6,09
684	F2	6,4
685	F2	5,63
686	F2	6,91
687	F2	7,01
688	F2	6,86
689	F2	6,39
690	F2	6,3
691	F2	5,75
692	F2	10,42
693	F2	6,8
694	F2	5,57
695	F2	2,15
696	F2	6,3
697	F2	5,92
698	F2	6,39
699	F2	6,71
700	F2	5,18
701	F2	5,26
702	F2	6,77
703	F2	5,22
704	F2	5,5
705	F2	8,11
706	F2	5,97
707	F2	2,38
708	F2	7,65
709	F2	6,22
710	F2	5,93
711	F2	6,92
712	F2	5,57
713	F2	5,82
714	F2	4,56
715	F2	6,22
716	F2	6,35
717	F2	6,56
718	F2	6,06

719	F2	5,47
720	F2	6,5
721	F2	6,24
722	F2	5,36
723	F2	8,21
724	F2	5,9
725	F2	5,37
726	F2	5,77
727	F2	5,82
728	F2	5,29
729	F2	5,91
730	F2	6,68
731	F2	6,37
732	F2	5,93
733	F2	6,8
734	F2	5,69
735	F2	5,93
736	F2	6,97
737	F2	7,34
738	F2	6,44
739	F2	6,29
740	F2	6,03
741	F2	6,64
742	F2	6,17
743	F2	5,34
744	F2	6,34
745	F2	4,12
746	F2	7,75
747	F2	6,67
748	F2	5,6
749	F2	5,7
750	F2	7,3
751	F2	5,86
752	F2	5,35
753	F2	6,81
754	F2	6,97
755	F2	6,09
756	F2	7,7
757	F2	2,78
758	F2	6,26
759	F2	9,89
760	F2	5,99
761	F2	5,54
762	F2	5,68
763	F2	6,51

764	F2	6,14
765	F2	4,29
766	F2	5,19
767	F2	5,18
768	F2	6,3
769	F2	6,19
770	F2	5,02
771	F2	5,37
772	F2	4,23
773	F2	5,98
774	F2	5,84
775	F2	6,42
776	F2	4,91
777	F2	5,92
778	F2	6,11
779	F2	5,39
780	F2	5,78
781	F2	4,65
782	F2	6,68
783	F2	4,5
784	F2	6,05
785	F2	6,26
786	F2	5,64
787	F2	5,87
788	F2	6,08
789	F2	5,78
790	F2	4,31
791	F2	6,49
792	F2	5,81
793	F2	4,79
794	F2	5,88
795	F2	0,97
796	F2	2,54
797	F2	3,36
798	F2	4,59
799	F2	3,83
800	F2	4,01
801	F2	4,06
802	F2	3,51
803	F2	3,51
804	F2	3,64
805	F2	4,32
806	F2	3,96
807	F2	3,64
808	F2	3,47

809	F2	4,66
810	F2	3,71
811	F2	3,66
812	F2	4,26
813	F2	3,77
814	F2	3,75
815	F2	3,92
816	F2	4,73
817	F2	4,07
818	F2	3,61
819	F2	4,38
820	F2	4,58
821	F2	4,07
822	F2	5,05
823	F2	5,33
824	F2	4,38
825	F2	5,05
826	F2	6,52
827	F2	5,06
828	F2	5,04
829	F2	4,9
830	F2	7,07
831	F2	4,78
832	F2	6,23
833	F2	7,09
834	F2	5,24
835	F2	6,19
836	F2	7,32
837	F2	6,46
838	F2	6,91
839	F2	6,78
840	F2	6,5
841	F2	6,39
842	F2	7,71
843	F2	6,77
844	F2	6,88
845	F2	6,9
846	F2	7,47
847	F2	6,63
848	F2	8,43
849	F2	7,28
850	F2	7,39
851	F2	8,15
852	F2	7,41
853	F2	7,13

854	F2	8,58
855	F2	8,26
856	F2	8,1
857	F2	8,4
858	F2	7,9
859	F2	7,8
860	F2	8,38
861	F2	8,84
862	F2	8,46
863	F2	9,87
864	F2	9,26
865	F2	9,01
866	F2	9,69
867	F2	9,51
868	F2	9,17
869	F2	9,74
870	F2	8,96
871	F2	8,23
872	F2	9,97
873	F2	9,76
874	F2	9,04
875	F2	10,72
876	F2	9,61
877	F2	8,79
878	F2	10,22
879	F2	9,92
880	F2	9,17
881	F2	10,59
882	F2	9,51
883	F2	8,93
884	F2	10,43
885	F2	9,47
886	F2	8,91
887	F2	9,48
888	F2	9,76
889	F2	9,31
890	F2	10,05
891	F2	9,23
892	F2	9,57
893	F2	10,34
894	F2	10,59
895	F2	10,41
896	F2	10,92
897	F2	10,12
898	F2	9,35

899	F2	10,81
900	F2	9,5
901	F2	10,81
902	F2	11,1
903	F2	10,62
904	F2	9,67
905	F2	10,89
906	F2	9,86
907	F2	10,07
908	F2	10,23
909	F2	10,01
910	F2	10,39
911	F2	10,99
912	F2	9,96
913	F2	10,2
914	F2	10,72
915	F2	10,13
916	F2	10,63
917	F2	10,45
918	F2	10,27
919	F2	9,41
920	F2	10,29
921	F2	10,16
922	F2	10,92
923	F2	10,61
924	F2	10,24
925	F2	10,69
926	F2	11,02
927	F2	9,91
928	F2	11,02
929	F2	10,77
930	F2	10,22
931	F2	10,58
932	F2	10,76
933	F2	10,6
934	F2	10,88
935	F2	9,96
936	F2	10,04
937	F2	10,93
938	F2	11,26
939	F2	10,01
940	F2	10,72
941	F2	11,56
942	F2	9,64
943	M1	1,23

944	M1	0,97
945	M1	0,41
946	M1	0,39
947	M1	1,12
948	M1	1
949	M1	0,24
950	M1	0,7
951	M1	1,54
952	M1	0,89
953	M1	0,01
954	M1	0,43
955	M1	0,51
956	M1	0,07
957	M1	0,28
958	M1	0,42
959	M1	0,49
960	M1	0,26
961	M1	0,64
962	M1	0,75
963	M1	0,32
964	M1	0,28
965	M1	1,79
966	M1	1,35
967	M1	0,02
968	M1	0,33
969	M1	0,71
970	M1	1,53
971	M1	0,11
972	M1	1,08
973	M1	1,59
974	M1	0,46
975	M1	0,29
976	M1	1,69
977	M1	0,53
978	M1	1,49
979	M1	1,32
980	M1	1,05
981	M1	0,63
982	M1	0,42
983	M1	1,75
984	M1	0,95
985	M1	1,4
986	M1	2,05
987	M1	2,17
988	M1	1,96

989	M1	1,76
990	M1	1,65
991	M1	1,45
992	M1	1,14
993	M1	3,12
994	M1	1,35
995	M1	1,93
996	M1	2,87
997	M1	2,48
998	M1	1,95
999	M1	3,23
1000	M1	1,8
1001	M1	1,92
1002	M1	3,02
1003	M1	2,6
1004	M1	2,34
1005	M1	3,61
1006	M1	3,88
1007	M1	3
1008	M1	2,74
1009	M1	3,11
1010	M1	3,35
1011	M1	2,44
1012	M1	3,24
1013	M1	3,62
1014	M1	2,87
1015	M1	3,6
1016	M1	3,15
1017	M1	2,42
1018	M1	3,22
1019	M1	4,09
1020	M1	3,15
1021	M1	2,56
1022	M1	4,1
1023	M1	3,61
1024	M1	3,27
1025	M1	4,1
1026	M1	3,42
1027	M1	2,65
1028	M1	3,67
1029	M1	4,6
1030	M1	2,65
1031	M1	2,09
1032	M1	4,26
1033	M1	3,13

1034	M1	2,47
1035	M1	3,39
1036	M1	3,15
1037	M1	2,37
1038	M1	3,9
1039	M1	3,52
1040	M1	3,57
1041	M1	4,72
1042	M1	2,83
1043	M1	3,2
1044	M1	4,08
1045	M1	3,74
1046	M1	3,42
1047	M1	3,38
1048	M1	3,41
1049	M1	3,51
1050	M1	3,94
1051	M1	3,71
1052	M1	3,64
1053	M1	3,46
1054	M1	3,46
1055	M1	2,55
1056	M1	3,75
1057	M1	3,2
1058	M1	3,29
1059	M1	3,67
1060	M1	3,37
1061	M1	2,92
1062	M1	2,73
1063	M1	2,55
1064	M1	3,07
1065	M1	3,51
1066	M1	2,88
1067	M1	3,66
1068	M1	3,33
1069	M1	2,91
1070	M1	2,93
1071	M1	3,49
1072	M1	2,69
1073	M1	2,73
1074	M1	3,83
1075	M1	3,42
1076	M1	3,33
1077	M1	3,56
1078	M1	3,48

1079	M1	2,92
1080	M1	3,58
1081	M1	3,67
1082	M1	2,98
1083	M1	3,64
1084	M1	3,09
1085	M1	2,9
1086	M1	3,89
1087	M1	3,82
1088	M1	3,29
1089	M1	3,58
1090	M1	3,39
1091	M1	3,25
1092	M1	4
1093	M1	2,75
1094	M1	4,1
1095	M1	3,39
1096	M1	3,65
1097	M1	2,72
1098	M1	3,96
1099	M1	3,46
1100	M1	2,98
1101	M1	3,85
1102	M1	3,25
1103	M1	3,71
1104	M1	3,88
1105	M1	2,84
1106	M1	3,58
1107	M1	3,41
1108	M1	3,8
1109	M1	2,95
1110	M1	4,31
1111	M1	3,97
1112	M1	2,6
1113	M1	3,78
1114	M1	3,43
1115	M1	3,3
1116	M1	3,84
1117	M1	4,12
1118	M1	2,63
1119	M1	3,54
1120	M1	3,69
1121	M1	3,34
1122	M1	3,05
1123	M1	3,34

1124	M1	3,07
1125	M1	2,74
1126	M1	3,15
1127	M1	2,29
1128	M1	3,78
1129	M1	3,89
1130	M1	3,05
1131	M1	2,37
1132	M1	3,63
1133	M1	3,2
1134	M1	2,56
1135	M1	4,05
1136	M1	3,36
1137	M1	2,42
1138	M1	2,6
1139	M1	1,9
1140	M1	2,71
1141	M1	2,67
1142	M1	3,17
1143	M1	2,22
1144	M1	3,24
1145	M1	2,7
1146	M1	2,11
1147	M1	2,88
1148	M1	2,56
1149	M1	1,46
1150	M1	3,07
1151	M1	2,59
1152	M1	2,88
1153	M1	2,31
1154	M1	3,52
1155	M1	2,44
1156	M1	2,08
1157	M1	3,11
1158	M1	2,24
1159	M1	1,76
1160	M1	2,77
1161	M1	2,34
1162	M1	1,92
1163	M1	2,5
1164	M1	2,86
1165	M1	1,98
1166	M1	2,67
1167	M1	2,26
1168	M1	2,35

1169	M1	2,47
1170	M1	2,47
1171	M1	1,78
1172	M1	2,23
1173	M1	3,16
1174	M1	2,23
1175	M1	3,2
1176	M1	2,71
1177	M1	2,16
1178	M1	2,16
1179	M1	3,6
1180	M1	2,32
1181	M1	2,46
1182	M1	2,67
1183	M1	2,66
1184	M1	1,87
1185	M1	2,96
1186	M1	2,22
1187	M1	2,22
1188	M1	2,45
1189	M1	2,48
1190	M1	2,04
1191	M1	3,11
1192	M1	2,38
1193	M1	2,38
1194	M1	3,28
1195	M1	2,75
1196	M1	1,3
1197	M1	3,01
1198	M1	2,49
1199	M1	2,64
1200	M1	2,79
1201	M1	1,99
1202	M1	2,1
1203	M1	2,7
1204	M1	2,95
1205	M1	0,86
1206	M1	3,18
1207	M1	2,39
1208	M1	1,84
1209	M1	3,02
1210	M1	2,72
1211	M1	2,08
1212	M1	1,94
1213	M1	2,26

1214	M1	1,39
1215	M1	1,72
1216	M1	2,22
1217	M1	2,64
1218	M1	1,76
1219	M1	2,08
1220	M1	2,37
1221	M1	2,09
1222	M1	2,81
1223	M1	1,9
1224	M1	1,56
1225	M1	2,82
1226	M1	2,69
1227	M1	1,81
1228	M1	1,82
1229	M1	2,64
1230	M1	2,28
1231	M1	2,42
1232	M1	2,63
1233	M1	1,12
1234	M1	3,08
1235	M1	2,09
1236	M1	1,9
1237	M1	2,17
1238	M1	2,95
1239	M1	1,68
1240	M1	3,28
1241	M1	2,55
1242	M1	1,61
1243	M1	2,34
1244	M1	2,04
1245	M1	2,2
1246	M1	1,99
1247	M1	2,91
1248	M1	2,49
1249	M1	2,06
1250	M1	3,13
1251	M1	2,54
1252	M1	2,58
1253	M1	2,2
1254	M1	1,32
1255	M1	1,51
1256	M1	0,75
1257	M1	1,17
1258	M1	1,17

1259	M1	1,56
1260	M1	2,19
1261	M1	1,1
1262	M1	2,59
1263	M1	2,31
1264	M1	1,66
1265	M1	2,7
1266	M1	2,09
1267	M1	3,56
1268	M1	1,8
1269	M1	2,9
1270	M1	1,63
1271	M1	2,38
1272	M1	2,41
1273	M1	2,4
1274	M1	2,45
1275	M1	2,12
1276	M1	2,8
1277	M1	2,23
1278	M1	3,98
1279	M1	3,24
1280	M1	2,67
1281	M1	2,99
1282	M1	3,65
1283	M1	3,12
1284	M1	2,93
1285	M1	3,29
1286	M1	2,77
1287	M1	2,55
1288	M1	4,53
1289	M1	3,44
1290	M1	3,51
1291	M1	3,95
1292	M1	2,99
1293	M1	2,79
1294	M1	2,81
1295	M1	3,89
1296	M1	2,9
1297	M1	3,82
1298	M1	3,7
1299	M1	3,85
1300	M1	3,11
1301	M1	3,94
1302	M1	4,2
1303	M1	2,84

1304	M1	4,51
1305	M1	4,67
1306	M1	2,91
1307	M1	4,29
1308	M1	3,48
1309	M1	3,82
1310	M1	2,75
1311	M1	3,26
1312	M1	3,87
1313	M1	2,9
1314	M1	3,71
1315	M1	3,84
1316	M1	3,11
1317	M1	3,22
1318	M1	2,9
1319	M1	4,27
1320	M1	2,99
1321	M1	3,88
1322	M1	2,99
1323	M1	2,54
1324	M1	4,31
1325	M1	3,28
1326	M1	2,58
1327	M1	2,93
1328	M1	2,85
1329	M1	3,85
1330	M1	2,85
1331	M1	2,24
1332	M1	2,71
1333	M1	2,03
1334	M1	2,11
1335	M1	2,08
1336	M1	2,47
1337	M1	2,46
1338	M1	1,97
1339	M1	2,79
1340	M1	2,16
1341	M1	2,14
1342	M1	3,21
1343	M1	1,8
1344	M1	2,73
1345	M1	1,44
1346	M1	1,48
1347	M1	3,13
1348	M1	1,66

1349	M1	2,16
1350	M1	3,34
1351	M1	0,91
1352	M1	3,29
1353	M1	2,4
1354	M1	2,13
1355	M1	2,23
1356	M1	2,07
1357	M1	2,12
1358	M1	1,79
1359	M1	2,11
1360	M1	3,66
1361	M1	1,4
1362	M1	3,04
1363	M1	2,37
1364	M1	1,33
1365	M1	2,84
1366	M1	1,08
1367	M1	1,95
1368	M1	1,16
1369	M1	2,11
1370	M1	1,03
1371	M1	0,97
1372	M1	2,62
1373	M1	0,88
1374	M1	1,31
1375	M1	1,98
1376	M1	1,8
1377	M1	1,7
1378	M1	1,82
1379	M1	2,05
1380	M1	1,92
1381	M1	2,23
1382	M1	3,04
1383	M1	2,02
1384	M1	2,17
1385	M1	2,48
1386	M1	2,46
1387	M1	2,35
1388	M1	2,65
1389	M1	2,4
1390	M1	2,16
1391	M1	3,15
1392	M1	3,23
1393	M1	2,93

1394	M1	3,33
1395	M1	2,9
1396	M1	3,08
1397	M1	3,68
1398	M1	3,57
1399	M1	3,02
1400	M1	3,61
1401	M1	3,66
1402	M1	2,32
1403	M1	3,96
1404	M1	4,45
1405	M1	4,36
1406	M1	3,24
1407	M1	4,72
1408	M1	3,67
1409	M1	3,39
1410	M1	3,48
1411	M1	2,78
1412	M1	4,05
1413	M2	0,15
1414	M2	0,12
1415	M2	0,55
1416	M2	0,77
1417	M2	0,89
1418	M2	1,08
1419	M2	0,02
1420	M2	0,44
1421	M2	1
1422	M2	0,16
1423	M2	0,05
1424	M2	0,67
1425	M2	1,3
1426	M2	0,59
1427	M2	0,47
1428	M2	1,28
1429	M2	0,24
1430	M2	0,01
1431	M2	0,46
1432	M2	0,05
1433	M2	0,35
1434	M2	0,66
1435	M2	0,63
1436	M2	0,2
1437	M2	0,75
1438	M2	0,18

1439	M2	0,4
1440	M2	0,11
1441	M2	0,09
1442	M2	0,84
1443	M2	0,01
1444	M2	0,71
1445	M2	0,5
1446	M2	1,37
1447	M2	1,09
1448	M2	0,67
1449	M2	0,51
1450	M2	0,17
1451	M2	0,44
1452	M2	0,89
1453	M2	1,53
1454	M2	1,87
1455	M2	1,11
1456	M2	1,05
1457	M2	1,27
1458	M2	0,13
1459	M2	1,54
1460	M2	1,59
1461	M2	1,39
1462	M2	1,53
1463	M2	2,16
1464	M2	1,32
1465	M2	1,98
1466	M2	2,77
1467	M2	1,39
1468	M2	3,22
1469	M2	2,75
1470	M2	1,16
1471	M2	3
1472	M2	1,88
1473	M2	2,37
1474	M2	3,71
1475	M2	3,51
1476	M2	2,78
1477	M2	4,28
1478	M2	3,34
1479	M2	3,74
1480	M2	4,1
1481	M2	3,19
1482	M2	4,47
1483	M2	4,56

1484	M2	4,11
1485	M2	4,84
1486	M2	4,07
1487	M2	4,3
1488	M2	4,44
1489	M2	5,23
1490	M2	3,6
1491	M2	6,43
1492	M2	4,43
1493	M2	4,74
1494	M2	6,33
1495	M2	4,95
1496	M2	5,71
1497	M2	6,17
1498	M2	5,96
1499	M2	6,31
1500	M2	5,46
1501	M2	5,5
1502	M2	6,84
1503	M2	6,31
1504	M2	5,73
1505	M2	7,09
1506	M2	6,39
1507	M2	6,83
1508	M2	5,63
1509	M2	5,45
1510	M2	7,37
1511	M2	6,05
1512	M2	6,17
1513	M2	6,74
1514	M2	5,33
1515	M2	7
1516	M2	6,04
1517	M2	5,87
1518	M2	6,27
1519	M2	5,49
1520	M2	6,05
1521	M2	6,53
1522	M2	4,86
1523	M2	6,58
1524	M2	6,23
1525	M2	5,62
1526	M2	6,01
1527	M2	7,72
1528	M2	7,36

1529	M2	6,33
1530	M2	7,19
1531	M2	6,46
1532	M2	5,47
1533	M2	5,98
1534	M2	6,74
1535	M2	5,37
1536	M2	7,02
1537	M2	7,1
1538	M2	6,72
1539	M2	6,07
1540	M2	5,99
1541	M2	6,82
1542	M2	5,58
1543	M2	5,4
1544	M2	6,86
1545	M2	5,25
1546	M2	6,92
1547	M2	6,5
1548	M2	7,11
1549	M2	7,15
1550	M2	6,42
1551	M2	7,06
1552	M2	6,78
1553	M2	5,71
1554	M2	6,57
1555	M2	6,05
1556	M2	6,66
1557	M2	6,07
1558	M2	5,6
1559	M2	6,27
1560	M2	5,53
1561	M2	5,49
1562	M2	7,27
1563	M2	5,71
1564	M2	6,31
1565	M2	5,12
1566	M2	7
1567	M2	6,67
1568	M2	4,99
1569	M2	5,84
1570	M2	5,03
1571	M2	4,87
1572	M2	5,08
1573	M2	4,83

1574	M2	5,9
1575	M2	5,52
1576	M2	5,93
1577	M2	5,4
1578	M2	5,04
1579	M2	5,78
1580	M2	4,22
1581	M2	4,81
1582	M2	5,14
1583	M2	3,96
1584	M2	5,43
1585	M2	5,13
1586	M2	6,07
1587	M2	4,9
1588	M2	4,6
1589	M2	4,43
1590	M2	4,41
1591	M2	5,17
1592	M2	4,64
1593	M2	5,2
1594	M2	5,67
1595	M2	3,86
1596	M2	4,51
1597	M2	4,36
1598	M2	4,7
1599	M2	5,78
1600	M2	3,75
1601	M2	4,26
1602	M2	3,51
1603	M2	4,27
1604	M2	5,15
1605	M2	6,48
1606	M2	5,83
1607	M2	3,57
1608	M2	4,63
1609	M2	5,83
1610	M2	4,63
1611	M2	5
1612	M2	4,51
1613	M2	5,24
1614	M2	3,75
1615	M2	4,08
1616	M2	4,95
1617	M2	3,7
1618	M2	4,49

1619	M2	5,75
1620	M2	4,58
1621	M2	5,71
1622	M2	4,55
1623	M2	4,92
1624	M2	5,2
1625	M2	3,71
1626	M2	4,36
1627	M2	3,67
1628	M2	4,83
1629	M2	5,04
1630	M2	4,04
1631	M2	5,16
1632	M2	3,75
1633	M2	4,08
1634	M2	4,42
1635	M2	4,64
1636	M2	4,66
1637	M2	4,81
1638	M2	4,55
1639	M2	3,93
1640	M2	3,39
1641	M2	4,69
1642	M2	4,67
1643	M2	4,78
1644	M2	4,12
1645	M2	4,01
1646	M2	4,76
1647	M2	3,68
1648	M2	4,84
1649	M2	3,76
1650	M2	3,41
1651	M2	5,41
1652	M2	2,96
1653	M2	3,71
1654	M2	4,6
1655	M2	3,17
1656	M2	3,92
1657	M2	3,43
1658	M2	4,62
1659	M2	3,62
1660	M2	3,72
1661	M2	4,09
1662	M2	4,58
1663	M2	4

1664	M2	3,68
1665	M2	4,07
1666	M2	3,69
1667	M2	3,29
1668	M2	4,24
1669	M2	2,52
1670	M2	3,87
1671	M2	4,72
1672	M2	3,14
1673	M2	4,38
1674	M2	3,58
1675	M2	3,33
1676	M2	4,91
1677	M2	3,52
1678	M2	3,66
1679	M2	3,96
1680	M2	2,67
1681	M2	4,41
1682	M2	4,2
1683	M2	3,59
1684	M2	3,58
1685	M2	3,91
1686	M2	2,9
1687	M2	2,83
1688	M2	4,07
1689	M2	3,12
1690	M2	3,17
1691	M2	3,96
1692	M2	4,63
1693	M2	4,81
1694	M2	3,56
1695	M2	4,76
1696	M2	4,31
1697	M2	3,49
1698	M2	4,56
1699	M2	3,45
1700	M2	3,26
1701	M2	4,64
1702	M2	2,41
1703	M2	5,08
1704	M2	2,35
1705	M2	3,05
1706	M2	3,46
1707	M2	3,2
1708	M2	5,17

1709	M2	4,3
1710	M2	4,59
1711	M2	3,72
1712	M2	3,24
1713	M2	5,51
1714	M2	2,47
1715	M2	3,56
1716	M2	5,02
1717	M2	4,13
1718	M2	4,41
1719	M2	3,19
1720	M2	4,23
1721	M2	5,35
1722	M2	3,35
1723	M2	4,45
1724	M2	3,3
1725	M2	4,41
1726	M2	5,01
1727	M2	2,97
1728	M2	1,55
1729	M2	4,84
1730	M2	3,8
1731	M2	3,96
1732	M2	3,33
1733	M2	4,6
1734	M2	3,89
1735	M2	3,73
1736	M2	4,77
1737	M2	3,71
1738	M2	4,55
1739	M2	3,59
1740	M2	3,59
1741	M2	5,06
1742	M2	3,05
1743	M2	4,22
1744	M2	3,82
1745	M2	4,42
1746	M2	5,1
1747	M2	3,42
1748	M2	3,71
1749	M2	4,83
1750	M2	3,84
1751	M2	4,17
1752	M2	3,7
1753	M2	4,75

1754	M2	3,03
1755	M2	2,95
1756	M2	2,96
1757	M2	3,36
1758	M2	2,51
1759	M2	3,23
1760	M2	2,94
1761	M2	2,6
1762	M2	2,81
1763	M2	3,38
1764	M2	2,24
1765	M2	2,62
1766	M2	3,73
1767	M2	3,66
1768	M2	2,56
1769	M2	2,57
1770	M2	2,98
1771	M2	3,25
1772	M2	3,45
1773	M2	3,3
1774	M2	4,2
1775	M2	3,54
1776	M2	3,82
1777	M2	4,66
1778	M2	3,47
1779	M2	2,54
1780	M2	3,82
1781	M2	3,65
1782	M2	4,64
1783	M2	3,6
1784	M2	4,2
1785	M2	4,33
1786	M2	4,08
1787	M2	4,13
1788	M2	4,14
1789	M2	3,84
1790	M2	4,77
1791	M2	4,23
1792	M2	3,94
1793	M2	4,4
1794	M2	5,26
1795	M2	4,39
1796	M2	4,36
1797	M2	3,71
1798	M2	5

1799	M2	3,68
1800	M2	5,03
1801	M2	4,12
1802	M2	3,78
1803	M2	4,28
1804	M2	4,43
1805	M2	4,92
1806	M2	3,99
1807	M2	3,27
1808	M2	4,13
1809	M2	2,51
1810	M2	0,58
1811	M2	0,06
1812	M2	3,55
1813	M2	3,99
1814	M2	4
1815	M2	4,23
1816	M2	4,06
1817	M2	5,2
1818	M2	5,38
1819	M2	3,89
1820	M2	1,94
1821	M2	1,46
1822	M2	0,08
1823	M2	0,23
1824	M2	0,59
1825	M2	1,01
1826	M2	0,3
1827	M2	0,49
1828	M2	0,33
1829	M2	0,33
1830	M2	0,28
1831	M2	0,25
1832	M2	0,1
1833	M2	0,46
1834	M2	0,11
1835	M2	1,13
1836	M2	0,53
1837	M2	1,51
1838	M2	1,1
1839	M2	0,5
1840	M2	1,3
1841	M2	0,07
1842	M2	0,75
1843	M2	1,47

1844	M2	1,05
1845	M2	2,72
1846	M2	1,72
1847	M2	1,53
1848	M2	2,53
1849	M2	2,13
1850	M2	2,36
1851	M2	3,13
1852	M2	2,7
1853	M2	3,84
1854	M2	3,04
1855	M2	3,59
1856	M2	3,42
1857	M2	3,63
1858	M2	5,3
1859	M2	2,61
1860	M2	5,43
1861	M2	4,96
1862	M2	3,89
1863	M2	3,53
1864	M2	6,28
1865	M2	3,58
1866	M2	4,87
1867	M2	3,84
1868	M2	4,44
1869	M2	5,51
1870	M2	3,73
1871	M2	4,44
1872	M2	5,19
1873	M2	6,27
1874	M2	3,4
1875	M2	3,2
1876	M2	6,06
1877	M2	5,63
1878	M2	3,92
1879	M2	4,36
1880	M2	5,68
1881	M2	6,1
1882	M2	5,06
