# Supplementary material for: Does a gender of Welwitschia mirabilis plants influence their photosynthetic activity?
Source: PLoS One. 2023 Sep 8;18(9):e0291122. doi: 10.1371/journal.pone.0291122 (PMC10490862; doi:10.1371/journal.pone.0291122)
Supplement: S2 Table — (PDF) [file pone.0291122.s002.pdf]

Measurement number	Specimen number	Parameter g_s
1	F1	27,59
2	F1	25,17
3	F1	25,33
4	F1	24,7
5	F1	24,29
6	F1	24,13
7	F1	25,17
8	F1	24,97
9	F1	24,98
10	F1	26,26
11	F1	25,65
12	F1	26,05
13	F1	26,48
14	F1	26,97
15	F1	27,64
16	F1	27,85
17	F1	28,77
18	F1	29,81
19	F1	30,15
20	F1	30,9
21	F1	31,3
22	F1	32,05
23	F1	33,45
24	F1	33,55
25	F1	34,09
26	F1	34,51
27	F1	35,14
28	F1	35,75
29	F1	37,74
30	F1	38,18
31	F1	38,92
32	F1	40,68
33	F1	41,6
34	F1	42,14
35	F1	42,32
36	F1	43,61
37	F1	43,49
38	F1	46,22
39	F1	45,13
40	F1	47,34
41	F1	48,09
42	F1	47,74
43	F1	50,12

44	F1	51,44
45	F1	52,25
46	F1	52,98
47	F1	53,85
48	F1	53,22
49	F1	55,44
50	F1	55,61
51	F1	53,99
52	F1	55,75
53	F1	57,83
54	F1	56,85
55	F1	59,1
56	F1	59,05
57	F1	58,99
58	F1	60,75
59	F1	58,69
60	F1	60,25
61	F1	61,36
62	F1	60,03
63	F1	63,51
64	F1	63,11
65	F1	62,2
66	F1	63,76
67	F1	64,84
68	F1	62,94
69	F1	64,06
70	F1	65,54
71	F1	66,17
72	F1	69,03
73	F1	69,76
74	F1	68,61
75	F1	71,7
76	F1	70,26
77	F1	70,83
78	F1	72,79
79	F1	73,4
80	F1	71,7
81	F1	72,73
82	F1	71,63
83	F1	73,61
84	F1	73,68
85	F1	71,49
86	F1	73,16
87	F1	72,28
88	F1	71,91

89	F1	71,65
90	F1	70,13
91	F1	68,47
92	F1	70,56
93	F1	67,55
94	F1	67,71
95	F1	68,58
96	F1	67,53
97	F1	68,99
98	F1	66,29
99	F1	67,65
100	F1	67,57
101	F1	65,65
102	F1	66,73
103	F1	66,36
104	F1	64,96
105	F1	65,44
106	F1	65,97
107	F1	64,29
108	F1	64,63
109	F1	65,58
110	F1	65,78
111	F1	67,5
112	F1	64,39
113	F1	66,38
114	F1	66,8
115	F1	67,84
116	F1	66,76
117	F1	67,64
118	F1	68,09
119	F1	67
120	F1	70,74
121	F1	68,9
122	F1	70,1
123	F1	71,1
124	F1	72,18
125	F1	72,92
126	F1	74,01
127	F1	72,27
128	F1	74,61
129	F1	76,23
130	F1	76,16
131	F1	76,87
132	F1	77,48
133	F1	76,35

134	F1	75,78
135	F1	77,32
136	F1	75,83
137	F1	77,63
138	F1	75,59
139	F1	75,95
140	F1	76,37
141	F1	73,33
142	F1	74,85
143	F1	73,19
144	F1	73,16
145	F1	70,64
146	F1	71,24
147	F1	69,22
148	F1	67,55
149	F1	64,43
150	F1	64,38
151	F1	64,25
152	F1	62,91
153	F1	63,2
154	F1	62,52
155	F1	61,44
156	F1	59,89
157	F1	59,5
158	F1	59,03
159	F1	57,42
160	F1	56,44
161	F1	55,65
162	F1	54,17
163	F1	53,61
164	F1	53,67
165	F1	51,62
166	F1	50,36
167	F1	50,79
168	F1	49,75
169	F1	48,8
170	F1	47,92
171	F1	45,96
172	F1	46,99
173	F1	44,95
174	F1	43,63
175	F1	43,39
176	F1	42,62
177	F1	41,84
178	F1	42,03

179	F1	40,41
180	F1	39,27
181	F1	37,32
182	F1	37,46
183	F1	36,1
184	F1	36,44
185	F1	34,72
186	F1	34,86
187	F1	32,47
188	F1	31,67
189	F1	31,26
190	F1	30,88
191	F1	30,51
192	F1	29,47
193	F1	28,2
194	F1	28,02
195	F1	27,08
196	F1	26,8
197	F1	26,72
198	F1	26,36
199	F1	25,26
200	F1	25,01
201	F1	22,49
202	F1	23,94
203	F1	23,22
204	F1	23,45
205	F1	22,28
206	F1	22,64
207	F1	22,03
208	F1	20,63
209	F1	21,79
210	F1	21,31
211	F1	20,89
212	F1	19,64
213	F1	19,64
214	F1	19,32
215	F1	18,94
216	F1	20,43
217	F1	18,46
218	F1	19,02
219	F1	20,17
220	F1	19,26
221	F1	20,24
222	F1	19,23
223	F1	19,78

224	F1	19
225	F1	19,3
226	F1	19,67
227	F1	18,62
228	F1	19,38
229	F1	19,3
230	F1	19,78
231	F1	19,66
232	F1	19,53
233	F1	19,38
234	F1	18,87
235	F1	19,11
236	F1	18,48
237	F1	18,34
238	F1	18,06
239	F1	17,53
240	F1	18,54
241	F1	17,93
242	F1	17,95
243	F1	17,87
244	F1	18,5
245	F1	17,86
246	F1	17,8
247	F1	18,1
248	F1	17,88
249	F1	18,29
250	F1	16,88
251	F1	17,12
252	F1	18,61
253	F1	17,09
254	F1	17,53
255	F1	17,09
256	F1	17,72
257	F1	16,99
258	F1	16,46
259	F1	17,99
260	F1	17,63
261	F1	17,07
262	F1	19,36
263	F1	17,71
264	F1	18,89
265	F1	17,23
266	F1	19,67
267	F1	18,71
268	F1	19,55

269	F1	18,22
270	F1	18,25
271	F1	18,61
272	F1	18,18
273	F1	18,92
274	F1	18,66
275	F1	18,25
276	F1	18,53
277	F1	19,07
278	F1	18,84
279	F1	17,7
280	F1	17,66
281	F1	18,37
282	F1	17,39
283	F1	18,89
284	F1	17,46
285	F1	17,89
286	F1	16,69
287	F1	17,27
288	F1	18,21
289	F1	16,67
290	F1	18,3
291	F1	16,53
292	F1	16,93
293	F1	15,95
294	F1	17,26
295	F1	16,19
296	F1	16,53
297	F1	15,69
298	F1	14,78
299	F1	16,85
300	F1	16,08
301	F1	15,82
302	F1	14,56
303	F1	14,87
304	F1	15,03
305	F1	15,43
306	F1	16,31
307	F1	15,31
308	F1	16,51
309	F1	14,93
310	F1	15,67
311	F1	15,08
312	F1	15,68
313	F1	16,24

314	F1	16,38
315	F1	16,46
316	F1	15,29
317	F1	16,62
318	F1	15,31
319	F1	15,46
320	F1	15,99
321	F1	16,25
322	F1	16,1
323	F1	15,14
324	F1	17,47
325	F1	16,09
326	F1	17,2
327	F1	16,08
328	F1	15,66
329	F1	16,86
330	F1	17,27
331	F1	17,85
332	F1	16,83
333	F1	17,06
334	F1	16,69
335	F1	16,13
336	F1	18,27
337	F1	16,14
338	F1	17,03
339	F1	11,99
340	F1	12,7
341	F1	15
342	F1	15,8
343	F1	16,79
344	F1	15,72
345	F1	17,34
346	F1	18,52
347	F1	18,42
348	F1	19,53
349	F1	20,89
350	F1	21,32
351	F1	22,61
352	F1	22,12
353	F1	23,59
354	F1	24,51
355	F1	23,87
356	F1	23,52
357	F1	24,55
358	F1	24,59

359	F1	24,98
360	F1	24,37
361	F1	26,01
362	F1	24,29
363	F1	24,99
364	F1	25,55
365	F1	24,31
366	F1	24,63
367	F1	24,24
368	F1	25,65
369	F1	25,41
370	F1	24,63
371	F1	25,16
372	F1	23,35
373	F1	24,32
374	F1	26,63
375	F1	25
376	F1	27,04
377	F1	27,79
378	F1	26,7
379	F1	27,82
380	F1	27,14
381	F1	27,46
382	F1	27,16
383	F1	27,44
384	F1	29,39
385	F1	28,99
386	F1	27,83
387	F1	31,1
388	F1	29,39
389	F1	30,36
390	F1	31,29
391	F1	29,19
392	F1	32,32
393	F1	31,09
394	F1	29,54
395	F1	31,36
396	F1	30,8
397	F1	32,8
398	F1	31,02
399	F1	32,23
400	F1	32,92
401	F1	32,6
402	F1	32,78
403	F1	32,68

404	F1	33,3
405	F1	33,81
406	F1	33,4
407	F1	34,06
408	F1	34,81
409	F1	33,09
410	F1	34,97
411	F1	33,33
412	F1	34,15
413	F1	35,46
414	F1	34,79
415	F1	34,56
416	F1	34,1
417	F1	35,21
418	F1	34,75
419	F1	35,49
420	F1	35,23
421	F1	35,66
422	F1	34,95
423	F1	34,5
424	F1	36,03
425	F1	34,86
426	F1	36,42
427	F1	35,69
428	F1	34,76
429	F1	35,62
430	F1	35,08
431	F1	35,18
432	F1	36,18
433	F1	34,45
434	F1	32,83
435	F1	34,77
436	F1	34,27
437	F1	35,96
438	F1	33,84
439	F1	34,56
440	F1	37,49
441	F1	33,7
442	F1	35,28
443	F1	34,34
444	F1	35,41
445	F1	35,49
446	F1	34,92
447	F1	35,38
448	F1	35,48

449	F1	34,74
450	F1	34,83
451	F1	35,67
452	F1	35,95
453	F1	35,77
454	F1	37,41
455	F1	36,91
456	F1	36,93
457	F1	37,17
458	F1	37,94
459	F1	38,52
460	F1	37,84
461	F1	39,54
462	F1	40,67
463	F1	39,51
464	F1	41,02
465	F1	42,67
466	F1	41,97
467	F1	42,55
468	F1	44,56
469	F1	41,12
470	F1	45,97
471	F1	43,16
472	F2	12,91
473	F2	12,39
474	F2	13,42
475	F2	13,69
476	F2	13,02
477	F2	13,87
478	F2	13,77
479	F2	14,85
480	F2	14,59
481	F2	15,88
482	F2	15,91
483	F2	16,18
484	F2	16,14
485	F2	15,61
486	F2	16,1
487	F2	15,55
488	F2	15,55
489	F2	16,42
490	F2	14,82
491	F2	15,74
492	F2	15,02
493	F2	15,92

494	F2	15,09
495	F2	15,32
496	F2	15,65
497	F2	15,6
498	F2	15,94
499	F2	15,46
500	F2	15,43
501	F2	15,24
502	F2	15,98
503	F2	16,25
504	F2	15,48
505	F2	17,12
506	F2	16,64
507	F2	16,59
508	F2	17,59
509	F2	17,41
510	F2	18,46
511	F2	18,97
512	F2	18,69
513	F2	19,54
514	F2	20,19
515	F2	20,3
516	F2	20,41
517	F2	20,4
518	F2	22,02
519	F2	21,91
520	F2	21,78
521	F2	23,13
522	F2	22,67
523	F2	24,77
524	F2	25,03
525	F2	25,25
526	F2	26,48
527	F2	27,74
528	F2	27,41
529	F2	28,61
530	F2	28,66
531	F2	29,02
532	F2	30,13
533	F2	31,34
534	F2	32,35
535	F2	32,79
536	F2	33,09
537	F2	33,42
538	F2	35,76

539	F2	36,63
540	F2	37,11
541	F2	37,81
542	F2	39,6
543	F2	37,81
544	F2	40,36
545	F2	41,39
546	F2	41,55
547	F2	42,76
548	F2	44,28
549	F2	44,68
550	F2	44,75
551	F2	45,84
552	F2	45,44
553	F2	47,57
554	F2	48,8
555	F2	48,52
556	F2	50,15
557	F2	51,41
558	F2	49,72
559	F2	52,14
560	F2	52,59
561	F2	52,29
562	F2	54,09
563	F2	53,87
564	F2	55,29
565	F2	56,28
566	F2	56,73
567	F2	58,07
568	F2	58,52
569	F2	59,51
570	F2	60,42
571	F2	60,82
572	F2	59,79
573	F2	62,54
574	F2	62,02
575	F2	61,65
576	F2	63,5
577	F2	65,22
578	F2	63,89
579	F2	64,87
580	F2	65,23
581	F2	64,65
582	F2	67,42
583	F2	68,51

584	F2	66,35
585	F2	68,63
586	F2	67,49
587	F2	68,76
588	F2	69,94
589	F2	70,12
590	F2	70,46
591	F2	70,66
592	F2	71,62
593	F2	72,31
594	F2	71,01
595	F2	72,91
596	F2	73,57
597	F2	73,93
598	F2	74,01
599	F2	73,84
600	F2	75,32
601	F2	75,16
602	F2	75,66
603	F2	76,63
604	F2	75,76
605	F2	75,76
606	F2	74,12
607	F2	75,37
608	F2	75,28
609	F2	76,5
610	F2	76,63
611	F2	77,16
612	F2	80,45
613	F2	79,19
614	F2	78,13
615	F2	80,29
616	F2	78,84
617	F2	79,03
618	F2	79,06
619	F2	80,22
620	F2	80,22
621	F2	80,93
622	F2	80,96
623	F2	80,96
624	F2	83,21
625	F2	80,68
626	F2	81,23
627	F2	83,7
628	F2	81,52

629	F2	82,83
630	F2	84,18
631	F2	80,86
632	F2	82,18
633	F2	84,17
634	F2	83,14
635	F2	81,99
636	F2	82
637	F2	82,32
638	F2	83,39
639	F2	83,68
640	F2	81,84
641	F2	83,21
642	F2	82,94
643	F2	83,24
644	F2	83,89
645	F2	82,4
646	F2	82,77
647	F2	83,39
648	F2	84,07
649	F2	81,92
650	F2	83,47
651	F2	80,53
652	F2	83,24
653	F2	81
654	F2	81,48
655	F2	81,72
656	F2	83,14
657	F2	81,56
658	F2	82,73
659	F2	82,22
660	F2	80,93
661	F2	82,81
662	F2	82,48
663	F2	84,09
664	F2	83,64
665	F2	82,36
666	F2	82,32
667	F2	82,29
668	F2	81,6
669	F2	82,11
670	F2	82,76
671	F2	82,27
672	F2	80,73
673	F2	82,16

674	F2	81,57
675	F2	81,91
676	F2	82,25
677	F2	81,79
678	F2	81,91
679	F2	81,68
680	F2	82,46
681	F2	83,68
682	F2	80,7
683	F2	82,1
684	F2	80,86
685	F2	81,91
686	F2	81,25
687	F2	82,41
688	F2	82,61
689	F2	81,5
690	F2	82,16
691	F2	80,66
692	F2	80,22
693	F2	81,61
694	F2	79,43
695	F2	79,88
696	F2	76,25
697	F2	76,16
698	F2	75,3
699	F2	74,79
700	F2	73,75
701	F2	75,81
702	F2	74,4
703	F2	73,52
704	F2	73,09
705	F2	75,02
706	F2	71,79
707	F2	74,56
708	F2	74,89
709	F2	73,37
710	F2	76,12
711	F2	76,04
712	F2	75,37
713	F2	75,94
714	F2	75,92
715	F2	77,14
716	F2	78,01
717	F2	78,22
718	F2	79,98

719	F2	78,2
720	F2	79,06
721	F2	78,53
722	F2	76,77
723	F2	78,81
724	F2	76,66
725	F2	77,26
726	F2	78,93
727	F2	79,33
728	F2	77,33
729	F2	77,35
730	F2	79,35
731	F2	77,35
732	F2	78,54
733	F2	78,24
734	F2	77,69
735	F2	77,8
736	F2	76,54
737	F2	78,01
738	F2	78,54
739	F2	78,13
740	F2	77,12
741	F2	78,11
742	F2	78,33
743	F2	77,44
744	F2	78,09
745	F2	76,83
746	F2	77,38
747	F2	77,12
748	F2	76,3
749	F2	75,38
750	F2	77,62
751	F2	77,15
752	F2	75,7
753	F2	76,76
754	F2	74,19
755	F2	74,51
756	F2	74,94
757	F2	71,88
758	F2	71,51
759	F2	73,07
760	F2	70,71
761	F2	70,49
762	F2	72,71
763	F2	70,47

764	F2	71,05
765	F2	72,29
766	F2	71,49
767	F2	71,58
768	F2	71,85
769	F2	71,96
770	F2	70,86
771	F2	71,64
772	F2	72,99
773	F2	70,97
774	F2	71,44
775	F2	73,37
776	F2	70,49
777	F2	70,79
778	F2	71,92
779	F2	72,57
780	F2	70
781	F2	73,05
782	F2	73,78
783	F2	71,2
784	F2	72,59
785	F2	74,49
786	F2	74,08
787	F2	75,03
788	F2	75,73
789	F2	75,71
790	F2	76,28
791	F2	76,96
792	F2	76,14
793	F2	76,22
794	F2	76,22
795	F2	55,47
796	F2	53,51
797	F2	50,24
798	F2	49,75
799	F2	45,45
800	F2	44,59
801	F2	43,04
802	F2	41,36
803	F2	40,39
804	F2	38,02
805	F2	38,37
806	F2	36,45
807	F2	34,33
808	F2	34,13

809	F2	34,27
810	F2	33,06
811	F2	32,79
812	F2	33,34
813	F2	32,31
814	F2	32,06
815	F2	32,56
816	F2	33,66
817	F2	32,62
818	F2	32,56
819	F2	33,77
820	F2	34,1
821	F2	34,11
822	F2	35,98
823	F2	36,17
824	F2	35,31
825	F2	36,24
826	F2	38,31
827	F2	37,81
828	F2	38,02
829	F2	40,25
830	F2	40,89
831	F2	40,23
832	F2	41,89
833	F2	43,65
834	F2	42,28
835	F2	43,75
836	F2	44,7
837	F2	45,17
838	F2	46,05
839	F2	46,94
840	F2	47,81
841	F2	48,59
842	F2	50,03
843	F2	49,34
844	F2	50,86
845	F2	51,4
846	F2	53,64
847	F2	53,4
848	F2	55,22
849	F2	55,24
850	F2	56,06
851	F2	59,28
852	F2	57,24
853	F2	58,54

854	F2	60,88
855	F2	61,28
856	F2	61,74
857	F2	62,51
858	F2	63,46
859	F2	63,56
860	F2	65,72
861	F2	66,59
862	F2	66,87
863	F2	68,96
864	F2	69,31
865	F2	69,92
866	F2	71,12
867	F2	71,07
868	F2	71,89
869	F2	73,34
870	F2	72,89
871	F2	74,08
872	F2	75,91
873	F2	76,99
874	F2	76,52
875	F2	77,39
876	F2	78,58
877	F2	77,99
878	F2	80,88
879	F2	80,29
880	F2	80,76
881	F2	83,77
882	F2	81,62
883	F2	82,95
884	F2	84,39
885	F2	80,91
886	F2	84,01
887	F2	84,85
888	F2	86,51
889	F2	84,99
890	F2	86,89
891	F2	87,11
892	F2	87,81
893	F2	90,1
894	F2	89,7
895	F2	90,53
896	F2	93,03
897	F2	93,13
898	F2	93,53

899	F2	94,78
900	F2	94,99
901	F2	95,23
902	F2	96,59
903	F2	95,22
904	F2	95,57
905	F2	98,52
906	F2	96,65
907	F2	96,77
908	F2	97,94
909	F2	98,06
910	F2	99,05
911	F2	99,55
912	F2	96,7
913	F2	98,97
914	F2	98,8
915	F2	99,16
916	F2	99,35
917	F2	99,7
918	F2	99,61
919	F2	99,94
920	F2	100,88
921	F2	101,25
922	F2	102,07
923	F2	101,12
924	F2	102,77
925	F2	104,15
926	F2	101,14
927	F2	101,37
928	F2	104,98
929	F2	103,53
930	F2	104,25
931	F2	103,48
932	F2	103,74
933	F2	106,39
934	F2	107,67
935	F2	107,39
936	F2	106,17
937	F2	108,01
938	F2	109,07
939	F2	107
940	F2	108,06
941	F2	108,09
942	F2	106,35
943	M1	17,66

944	M1	16,85
945	M1	15,79
946	M1	15,95
947	M1	14,34
948	M1	14,34
949	M1	12,88
950	M1	12,51
951	M1	11,54
952	M1	12,7
953	M1	3,57
954	M1	11,51
955	M1	11,17
956	M1	10,96
957	M1	10,63
958	M1	11,4
959	M1	11,15
960	M1	10,94
961	M1	11,26
962	M1	10,86
963	M1	11,58
964	M1	10,17
965	M1	10,89
966	M1	11,4
967	M1	11,79
968	M1	10,15
969	M1	11,6
970	M1	12,47
971	M1	12,65
972	M1	11,71
973	M1	12,3
974	M1	13,24
975	M1	12,8
976	M1	13,42
977	M1	14,15
978	M1	14,08
979	M1	15,34
980	M1	14,62
981	M1	13,58
982	M1	14,26
983	M1	14,76
984	M1	15,89
985	M1	16,02
986	M1	16,6
987	M1	16,81
988	M1	17,5

989	M1	17,94
990	M1	17,84
991	M1	18,2
992	M1	17,86
993	M1	17,61
994	M1	19,24
995	M1	18,89
996	M1	19,69
997	M1	20,97
998	M1	20,44
999	M1	21,29
1000	M1	21,3
1001	M1	21,21
1002	M1	22,09
1003	M1	22,32
1004	M1	22,3
1005	M1	22,95
1006	M1	23,95
1007	M1	23,82
1008	M1	23,07
1009	M1	23,73
1010	M1	23,58
1011	M1	23,39
1012	M1	22,89
1013	M1	24,49
1014	M1	24,88
1015	M1	24,62
1016	M1	25,23
1017	M1	25,18
1018	M1	24,71
1019	M1	25,97
1020	M1	26,32
1021	M1	25,49
1022	M1	25,91
1023	M1	26,23
1024	M1	26,3
1025	M1	25,73
1026	M1	27,62
1027	M1	27,07
1028	M1	25,79
1029	M1	27,28
1030	M1	27,32
1031	M1	26,64
1032	M1	25,36
1033	M1	26,32

1034	M1	25,84
1035	M1	25,1
1036	M1	26,9
1037	M1	25,88
1038	M1	25,19
1039	M1	26,69
1040	M1	24,8
1041	M1	25,27
1042	M1	26,21
1043	M1	25,55
1044	M1	25,1
1045	M1	25,89
1046	M1	25,87
1047	M1	25,39
1048	M1	25,75
1049	M1	24,29
1050	M1	25,13
1051	M1	24,78
1052	M1	26,24
1053	M1	25,23
1054	M1	26,58
1055	M1	25,63
1056	M1	24,38
1057	M1	25,51
1058	M1	24,87
1059	M1	23,8
1060	M1	24,45
1061	M1	24,5
1062	M1	23,77
1063	M1	23,71
1064	M1	22,75
1065	M1	22,52
1066	M1	21,85
1067	M1	22,42
1068	M1	22,87
1069	M1	22,26
1070	M1	22,56
1071	M1	21,92
1072	M1	22,16
1073	M1	21,42
1074	M1	21,69
1075	M1	22,57
1076	M1	21,56
1077	M1	21,57
1078	M1	21,72

1079	M1	21,2
1080	M1	20,24
1081	M1	22,15
1082	M1	21,12
1083	M1	20,96
1084	M1	21,2
1085	M1	20,99
1086	M1	21,66
1087	M1	21,89
1088	M1	21,88
1089	M1	20,76
1090	M1	21,68
1091	M1	21,83
1092	M1	21,39
1093	M1	22,18
1094	M1	20,65
1095	M1	22,55
1096	M1	21,43
1097	M1	22,69
1098	M1	21,33
1099	M1	22,59
1100	M1	23,1
1101	M1	22,34
1102	M1	22,95
1103	M1	22,13
1104	M1	22,99
1105	M1	21,41
1106	M1	22,36
1107	M1	22,48
1108	M1	22,59
1109	M1	23
1110	M1	22,61
1111	M1	23,84
1112	M1	23,54
1113	M1	21,7
1114	M1	23,06
1115	M1	23,01
1116	M1	22,93
1117	M1	23,09
1118	M1	23,49
1119	M1	22,46
1120	M1	23,05
1121	M1	22,39
1122	M1	22,08
1123	M1	22,1

1124	M1	22,13
1125	M1	22,45
1126	M1	21,62
1127	M1	22,35
1128	M1	20,68
1129	M1	22,05
1130	M1	22,43
1131	M1	20,93
1132	M1	20,35
1133	M1	21,21
1134	M1	22,14
1135	M1	20,61
1136	M1	21,25
1137	M1	20,54
1138	M1	20,77
1139	M1	20,59
1140	M1	20,44
1141	M1	19,83
1142	M1	19,93
1143	M1	19,88
1144	M1	18,82
1145	M1	20,22
1146	M1	20,04
1147	M1	19,09
1148	M1	19,49
1149	M1	19,46
1150	M1	18,47
1151	M1	19,81
1152	M1	19,18
1153	M1	19,37
1154	M1	19,18
1155	M1	19,1
1156	M1	18,69
1157	M1	18,19
1158	M1	19,9
1159	M1	18,76
1160	M1	18,21
1161	M1	19,13
1162	M1	17,56
1163	M1	18,29
1164	M1	18,19
1165	M1	18,62
1166	M1	17,11
1167	M1	18,25
1168	M1	18,4

1169	M1	17,88
1170	M1	18,18
1171	M1	18,15
1172	M1	17,46
1173	M1	18,64
1174	M1	17,72
1175	M1	18,52
1176	M1	18,84
1177	M1	18,64
1178	M1	19,24
1179	M1	18,32
1180	M1	18,91
1181	M1	18,23
1182	M1	18,54
1183	M1	19,06
1184	M1	18,75
1185	M1	19,02
1186	M1	19,69
1187	M1	18,5
1188	M1	18,4
1189	M1	19,82
1190	M1	19,36
1191	M1	19,13
1192	M1	19,71
1193	M1	19,3
1194	M1	18,37
1195	M1	19,8
1196	M1	19,7
1197	M1	17,9
1198	M1	19,41
1199	M1	19,3
1200	M1	19,19
1201	M1	18,65
1202	M1	18,99
1203	M1	18,16
1204	M1	18,66
1205	M1	19,42
1206	M1	17,71
1207	M1	18,82
1208	M1	18,22
1209	M1	18,07
1210	M1	17,76
1211	M1	19,73
1212	M1	18,54
1213	M1	18,83

1214	M1	18,73
1215	M1	18,3
1216	M1	18,3
1217	M1	18,73
1218	M1	18,33
1219	M1	18,84
1220	M1	18,75
1221	M1	18,77
1222	M1	17,46
1223	M1	18,78
1224	M1	18,55
1225	M1	18,99
1226	M1	18,65
1227	M1	17,92
1228	M1	17,85
1229	M1	18,04
1230	M1	17,83
1231	M1	18,19
1232	M1	18,24
1233	M1	18,75
1234	M1	17,74
1235	M1	18,6
1236	M1	18,53
1237	M1	17,84
1238	M1	18,28
1239	M1	18,81
1240	M1	18,61
1241	M1	18,48
1242	M1	19,03
1243	M1	17,33
1244	M1	18,28
1245	M1	18,04
1246	M1	18,08
1247	M1	18,07
1248	M1	18,73
1249	M1	18
1250	M1	18,25
1251	M1	19,27
1252	M1	17,8
1253	M1	17,84
1254	M1	16,67
1255	M1	16,46
1256	M1	17,03
1257	M1	17,42
1258	M1	17,29

1259	M1	17,49
1260	M1	17,62
1261	M1	18,22
1262	M1	18,55
1263	M1	18,8
1264	M1	18,63
1265	M1	19,56
1266	M1	18,64
1267	M1	19,92
1268	M1	20,09
1269	M1	20,49
1270	M1	21,58
1271	M1	20,63
1272	M1	22,66
1273	M1	23,25
1274	M1	24,12
1275	M1	23,14
1276	M1	22,59
1277	M1	23,4
1278	M1	22,95
1279	M1	24,13
1280	M1	23,65
1281	M1	23,05
1282	M1	24,16
1283	M1	24,42
1284	M1	23,92
1285	M1	24,41
1286	M1	25,56
1287	M1	24,96
1288	M1	26,07
1289	M1	27,03
1290	M1	27,53
1291	M1	27,42
1292	M1	28,09
1293	M1	28,71
1294	M1	26,65
1295	M1	28,48
1296	M1	28,13
1297	M1	27,77
1298	M1	28,27
1299	M1	28,15
1300	M1	28,1
1301	M1	29,13
1302	M1	28,34
1303	M1	28,82

1304	M1	27,89
1305	M1	29,53
1306	M1	28,92
1307	M1	30,21
1308	M1	29,46
1309	M1	29,58
1310	M1	29,54
1311	M1	30,24
1312	M1	29,65
1313	M1	28,55
1314	M1	29,73
1315	M1	29,75
1316	M1	28,68
1317	M1	28,78
1318	M1	29,13
1319	M1	29,1
1320	M1	28,01
1321	M1	28,62
1322	M1	28,75
1323	M1	27,94
1324	M1	28,2
1325	M1	28,27
1326	M1	27,87
1327	M1	27,88
1328	M1	26,87
1329	M1	28,38
1330	M1	25,91
1331	M1	26,87
1332	M1	25,97
1333	M1	24,5
1334	M1	25,97
1335	M1	26,31
1336	M1	24,62
1337	M1	25,27
1338	M1	24,22
1339	M1	24,77
1340	M1	24,11
1341	M1	23,6
1342	M1	23,68
1343	M1	23,69
1344	M1	22,96
1345	M1	22,95
1346	M1	22,55
1347	M1	22,38
1348	M1	22,82

1349	M1	22,56
1350	M1	21,72
1351	M1	22,85
1352	M1	23,12
1353	M1	22,27
1354	M1	23,06
1355	M1	21,39
1356	M1	21,59
1357	M1	22,36
1358	M1	21,72
1359	M1	22,11
1360	M1	22,02
1361	M1	21,15
1362	M1	21,84
1363	M1	21,36
1364	M1	21,22
1365	M1	21,26
1366	M1	20,6
1367	M1	20,66
1368	M1	19,81
1369	M1	20,28
1370	M1	21,25
1371	M1	19,3
1372	M1	20,21
1373	M1	20,69
1374	M1	19,34
1375	M1	20,26
1376	M1	19,36
1377	M1	19,17
1378	M1	48,46
1379	M1	41,03
1380	M1	37,06
1381	M1	34,38
1382	M1	30,14
1383	M1	28,01
1384	M1	25,15
1385	M1	24,83
1386	M1	23,17
1387	M1	21,85
1388	M1	19,85
1389	M1	20,84
1390	M1	18,99
1391	M1	18,28
1392	M1	18,58
1393	M1	18,2

1394	M1	16,85
1395	M1	18
1396	M1	17,22
1397	M1	17,6
1398	M1	18,24
1399	M1	17,68
1400	M1	17,22
1401	M1	18,18
1402	M1	18,25
1403	M1	18,05
1404	M1	18,47
1405	M1	19,52
1406	M1	18,82
1407	M1	20,01
1408	M1	20,6
1409	M1	20,53
1410	M1	21,05
1411	M1	21,88
1412	M1	22,12
1413	M2	8,55
1414	M2	8,02
1415	M2	7,14
1416	M2	6,85
1417	M2	7,48
1418	M2	6,42
1419	M2	6,43
1420	M2	6,25
1421	M2	6,22
1422	M2	7,18
1423	M2	6,19
1424	M2	6,45
1425	M2	6,45
1426	M2	6,98
1427	M2	6,35
1428	M2	5,62
1429	M2	6,02
1430	M2	6,7
1431	M2	6,4
1432	M2	6,41
1433	M2	6,24
1434	M2	6,5
1435	M2	7,14
1436	M2	7,03
1437	M2	5,89
1438	M2	6,47

1439	M2	7,2
1440	M2	6,51
1441	M2	7,02
1442	M2	7,9
1443	M2	7,07
1444	M2	8,42
1445	M2	8,19
1446	M2	7,63
1447	M2	8,37
1448	M2	8,66
1449	M2	7,39
1450	M2	8,1
1451	M2	9,47
1452	M2	8,91
1453	M2	9,64
1454	M2	9,95
1455	M2	10,08
1456	M2	10,46
1457	M2	10,27
1458	M2	10,87
1459	M2	11,47
1460	M2	12,24
1461	M2	12,16
1462	M2	12,55
1463	M2	13,86
1464	M2	12,84
1465	M2	14,5
1466	M2	15
1467	M2	15,39
1468	M2	16,38
1469	M2	16,53
1470	M2	16,05
1471	M2	17,04
1472	M2	17,24
1473	M2	17,21
1474	M2	19,82
1475	M2	19,87
1476	M2	20,43
1477	M2	21,93
1478	M2	21,58
1479	M2	22,56
1480	M2	23,39
1481	M2	23,01
1482	M2	24,99
1483	M2	25,19

1484	M2	25,43
1485	M2	26,11
1486	M2	26,43
1487	M2	27,04
1488	M2	27,84
1489	M2	28,33
1490	M2	28,85
1491	M2	30,35
1492	M2	29,96
1493	M2	30,04
1494	M2	31,75
1495	M2	31,77
1496	M2	32,42
1497	M2	33,11
1498	M2	32,48
1499	M2	33,52
1500	M2	32,54
1501	M2	33,43
1502	M2	34,84
1503	M2	34,04
1504	M2	33,88
1505	M2	35,45
1506	M2	34,83
1507	M2	36,1
1508	M2	35,58
1509	M2	35,51
1510	M2	36,29
1511	M2	35,11
1512	M2	35,37
1513	M2	36,67
1514	M2	35,21
1515	M2	36,98
1516	M2	35,82
1517	M2	35,75
1518	M2	36,63
1519	M2	35,87
1520	M2	36,26
1521	M2	36,71
1522	M2	37,61
1523	M2	37,87
1524	M2	36,01
1525	M2	35,85
1526	M2	36,65
1527	M2	36,43
1528	M2	37,89

1529	M2	36,97
1530	M2	37,44
1531	M2	37,05
1532	M2	36,2
1533	M2	36,73
1534	M2	36,94
1535	M2	36,48
1536	M2	37,99
1537	M2	37,31
1538	M2	37,15
1539	M2	37,29
1540	M2	36,16
1541	M2	37,19
1542	M2	35,9
1543	M2	36,51
1544	M2	37,28
1545	M2	37,05
1546	M2	37,26
1547	M2	36,1
1548	M2	37,73
1549	M2	37,56
1550	M2	36,94
1551	M2	37,73
1552	M2	37,14
1553	M2	36,33
1554	M2	36,96
1555	M2	36,85
1556	M2	37
1557	M2	35,97
1558	M2	37,21
1559	M2	36,71
1560	M2	36,09
1561	M2	36,45
1562	M2	36,96
1563	M2	37,06
1564	M2	35,73
1565	M2	36,35
1566	M2	35,65
1567	M2	36,5
1568	M2	35,66
1569	M2	35,61
1570	M2	35,1
1571	M2	34,32
1572	M2	34,31
1573	M2	33,91

1574	M2	34,43
1575	M2	33,38
1576	M2	34,05
1577	M2	32,47
1578	M2	33,11
1579	M2	32,21
1580	M2	30,42
1581	M2	31,84
1582	M2	30,78
1583	M2	29,83
1584	M2	29,41
1585	M2	30,45
1586	M2	29,43
1587	M2	29,24
1588	M2	29,4
1589	M2	28,52
1590	M2	28,57
1591	M2	28,14
1592	M2	28,17
1593	M2	28,28
1594	M2	27,87
1595	M2	26,65
1596	M2	26,6
1597	M2	27
1598	M2	27,26
1599	M2	28,18
1600	M2	26,5
1601	M2	26,8
1602	M2	26,51
1603	M2	25,83
1604	M2	26,67
1605	M2	26,69
1606	M2	26,91
1607	M2	25,9
1608	M2	25,87
1609	M2	26,99
1610	M2	25,86
1611	M2	27,02
1612	M2	27,03
1613	M2	27,18
1614	M2	25,53
1615	M2	25,89
1616	M2	25,75
1617	M2	25,33
1618	M2	26,79

1619	M2	26,77
1620	M2	26,48
1621	M2	27,82
1622	M2	26,23
1623	M2	26,52
1624	M2	26,89
1625	M2	25,68
1626	M2	25,4
1627	M2	25,14
1628	M2	26,1
1629	M2	26,24
1630	M2	25,78
1631	M2	25,97
1632	M2	25,12
1633	M2	24,8
1634	M2	25,5
1635	M2	25,73
1636	M2	26,47
1637	M2	25,3
1638	M2	25,61
1639	M2	24,07
1640	M2	24,66
1641	M2	24,49
1642	M2	24,5
1643	M2	25,14
1644	M2	24,54
1645	M2	24,49
1646	M2	25,06
1647	M2	23,95
1648	M2	24,47
1649	M2	22,79
1650	M2	24,07
1651	M2	24,21
1652	M2	23,21
1653	M2	23,74
1654	M2	23,67
1655	M2	22,15
1656	M2	23,33
1657	M2	21,96
1658	M2	22,42
1659	M2	22,27
1660	M2	21,69
1661	M2	22,86
1662	M2	22,67
1663	M2	22,33

1664	M2	21,64
1665	M2	21,56
1666	M2	21,84
1667	M2	21,52
1668	M2	21,4
1669	M2	21
1670	M2	21,71
1671	M2	22,53
1672	M2	21,41
1673	M2	22,41
1674	M2	21,04
1675	M2	20,88
1676	M2	21,99
1677	M2	21,35
1678	M2	21,85
1679	M2	21,54
1680	M2	20,79
1681	M2	22,14
1682	M2	21,86
1683	M2	21,82
1684	M2	21,01
1685	M2	22,21
1686	M2	21,6
1687	M2	21,51
1688	M2	21,38
1689	M2	22,08
1690	M2	20,61
1691	M2	21,61
1692	M2	22,52
1693	M2	20,82
1694	M2	21,19
1695	M2	20,57
1696	M2	21,04
1697	M2	21,59
1698	M2	21,2
1699	M2	20,02
1700	M2	20,8
1701	M2	21,5
1702	M2	20,58
1703	M2	20,92
1704	M2	20,27
1705	M2	20,46
1706	M2	22,55
1707	M2	21,04
1708	M2	22,64

1709	M2	21,69
1710	M2	21,95
1711	M2	22,29
1712	M2	22,18
1713	M2	21,98
1714	M2	21,51
1715	M2	21,64
1716	M2	22,89
1717	M2	22,55
1718	M2	22,57
1719	M2	21,42
1720	M2	22,25
1721	M2	23,02
1722	M2	21,93
1723	M2	22,25
1724	M2	22,12
1725	M2	21,95
1726	M2	23,08
1727	M2	22,39
1728	M2	21,95
1729	M2	22,56
1730	M2	21,73
1731	M2	21,96
1732	M2	21,61
1733	M2	22,92
1734	M2	21,94
1735	M2	22,83
1736	M2	23,4
1737	M2	22,15
1738	M2	22,81
1739	M2	21,88
1740	M2	23,34
1741	M2	24,23
1742	M2	21,8
1743	M2	22,93
1744	M2	22,15
1745	M2	22,6
1746	M2	23,42
1747	M2	22,85
1748	M2	23,07
1749	M2	23,13
1750	M2	22,64
1751	M2	23,2
1752	M2	22,27
1753	M2	31,6

1754	M2	29,3
1755	M2	26,65
1756	M2	24,59
1757	M2	24,08
1758	M2	23,05
1759	M2	21,95
1760	M2	21,94
1761	M2	21,03
1762	M2	20,56
1763	M2	20,73
1764	M2	20,09
1765	M2	19,62
1766	M2	20,8
1767	M2	20,42
1768	M2	19,55
1769	M2	20,8
1770	M2	20,3
1771	M2	20,47
1772	M2	20,43
1773	M2	20,58
1774	M2	21,65
1775	M2	20,77
1776	M2	21,17
1777	M2	21,51
1778	M2	21,89
1779	M2	22,09
1780	M2	22,94
1781	M2	22,55
1782	M2	23,1
1783	M2	23,18
1784	M2	23,83
1785	M2	24,34
1786	M2	24,38
1787	M2	24,12
1788	M2	24,55
1789	M2	24,06
1790	M2	25,35
1791	M2	25,11
1792	M2	24,89
1793	M2	25,43
1794	M2	25,82
1795	M2	25,28
1796	M2	25,67
1797	M2	25,31
1798	M2	25,98

1799	M2	25,23
1800	M2	25,69
1801	M2	25,11
1802	M2	25,54
1803	M2	26,59
1804	M2	25,61
1805	M2	26,62
1806	M2	25,39
1807	M2	25,78
1808	M2	25,76
1809	M2	24,88
1810	M2	25,22
1811	M2	24,78
1812	M2	22,6
1813	M2	21,22
1814	M2	21,3
1815	M2	19,1
1816	M2	18,87
1817	M2	15,56
1818	M2	14,6
1819	M2	14,3
1820	M2	11,85
1821	M2	11,94
1822	M2	12,01
1823	M2	10,11
1824	M2	10,35
1825	M2	9,53
1826	M2	10
1827	M2	9,45
1828	M2	8,91
1829	M2	9,65
1830	M2	8,36
1831	M2	9,04
1832	M2	9,32
1833	M2	9,23
1834	M2	9,49
1835	M2	9,63
1836	M2	9,54
1837	M2	10,3
1838	M2	10,62
1839	M2	9,95
1840	M2	11,32
1841	M2	10,67
1842	M2	10,83
1843	M2	12,14

1844	M2	11,57
1845	M2	13,21
1846	M2	12,38
1847	M2	13,48
1848	M2	14,15
1849	M2	14,7
1850	M2	15,05
1851	M2	16,73
1852	M2	16,58
1853	M2	18,02
1854	M2	17,22
1855	M2	18,47
1856	M2	18,99
1857	M2	18,61
1858	M2	20,95
1859	M2	19,27
1860	M2	20,73
1861	M2	21,76
1862	M2	20,69
1863	M2	22,73
1864	M2	22,79
1865	M2	22,3
1866	M2	23,81
1867	M2	22,81
1868	M2	24,3
1869	M2	24,87
1870	M2	23,94
1871	M2	24,01
1872	M2	24,89
1873	M2	24,93
1874	M2	25,8
1875	M2	25,39
1876	M2	24,3
1877	M2	26,8
1878	M2	25,44
1879	M2	25,74
1880	M2	26,72
1881	M2	26,92
1882	M2	27
