# Supplementary material for: Does a gender of Welwitschia mirabilis plants influence their photosynthetic activity?
Source: PLoS One. 2023 Sep 8;18(9):e0291122. doi: 10.1371/journal.pone.0291122 (PMC10490862; doi:10.1371/journal.pone.0291122)
Supplement: S3 Table — (PDF) [file pone.0291122.s003.pdf]

Measurement number	Specimen number	Parameter C_i
1	F1	227,9
2	F1	262,6
3	F1	248,3
4	F1	218,1
5	F1	218,2
6	F1	261,8
7	F1	252,8
8	F1	215
9	F1	239,4
10	F1	175,3
11	F1	207,7
12	F1	213,7
13	F1	168,1
14	F1	196
15	F1	223,2
16	F1	147,9
17	F1	143,3
18	F1	189,1
19	F1	223,7
20	F1	162,3
21	F1	199,4
22	F1	204,4
23	F1	170,5
24	F1	177,9
25	F1	215,7
26	F1	182,2
27	F1	194
28	F1	183,2
29	F1	175,5
30	F1	184
31	F1	198,9
32	F1	182,1
33	F1	180,3
34	F1	197,4
35	F1	204,9
36	F1	180,1
37	F1	205,3
38	F1	171,5
39	F1	187,4
40	F1	195,1
41	F1	184,5
42	F1	208,8
43	F1	215,5

44	F1	192,6
45	F1	197,4
46	F1	208,6
47	F1	192,8
48	F1	210,2
49	F1	202,2
50	F1	174
51	F1	222,4
52	F1	197
53	F1	175,9
54	F1	212,2
55	F1	190
56	F1	165,3
57	F1	196,4
58	F1	180,7
59	F1	211,5
60	F1	209,5
61	F1	217,1
62	F1	202,7
63	F1	207,6
64	F1	197
65	F1	203,3
66	F1	201,2
67	F1	183,5
68	F1	215,7
69	F1	219,9
70	F1	204,3
71	F1	219,9
72	F1	212,7
73	F1	238,8
74	F1	240,5
75	F1	219,5
76	F1	246,6
77	F1	247,3
78	F1	226,6
79	F1	231,4
80	F1	243,1
81	F1	234,7
82	F1	249,5
83	F1	224
84	F1	215,5
85	F1	255,2
86	F1	242,5
87	F1	268,5
88	F1	245,7

89	F1	209
90	F1	236,8
91	F1	246
92	F1	200,8
93	F1	239,1
94	F1	230,6
95	F1	217,1
96	F1	228,4
97	F1	211,8
98	F1	193,1
99	F1	208,7
100	F1	206,5
101	F1	239,2
102	F1	232,5
103	F1	195,8
104	F1	231,7
105	F1	200,8
106	F1	190,3
107	F1	220,1
108	F1	233,3
109	F1	191,2
110	F1	214,2
111	F1	206,8
112	F1	209,5
113	F1	213,4
114	F1	201,7
115	F1	218,3
116	F1	239,4
117	F1	192,8
118	F1	220,6
119	F1	220,2
120	F1	208,3
121	F1	212,5
122	F1	221,3
123	F1	210,2
124	F1	225
125	F1	217,9
126	F1	220
127	F1	230,5
128	F1	216,6
129	F1	206,1
130	F1	227,7
131	F1	238,8
132	F1	205,6
133	F1	213,3

134	F1	232,5
135	F1	220,3
136	F1	248,7
137	F1	225,4
138	F1	240,5
139	F1	240
140	F1	224,3
141	F1	235,7
142	F1	224,6
143	F1	286
144	F1	242,9
145	F1	235,7
146	F1	202,4
147	F1	237,3
148	F1	214,5
149	F1	218,8
150	F1	222,6
151	F1	208,3
152	F1	212,7
153	F1	187,2
154	F1	195,9
155	F1	209,5
156	F1	213,5
157	F1	211,4
158	F1	227,3
159	F1	205,6
160	F1	222,7
161	F1	216
162	F1	188,8
163	F1	212,9
164	F1	205,5
165	F1	216,1
166	F1	186,4
167	F1	207,6
168	F1	187,6
169	F1	193,1
170	F1	180,1
171	F1	197,1
172	F1	209,8
173	F1	200,6
174	F1	180,2
175	F1	188,3
176	F1	215,4
177	F1	184,6
178	F1	167,9

179	F1	214,8
180	F1	138,8
181	F1	184,6
182	F1	228,3
183	F1	146,9
184	F1	175,8
185	F1	186,2
186	F1	198,2
187	F1	205
188	F1	131,3
189	F1	186,5
190	F1	157,3
191	F1	196,4
192	F1	199,7
193	F1	177,1
194	F1	192,6
195	F1	212,5
196	F1	191,2
197	F1	162,3
198	F1	186,5
199	F1	305,5
200	F1	233,7
201	F1	184,5
202	F1	197,2
203	F1	190,8
204	F1	149,6
205	F1	192,4
206	F1	207,2
207	F1	197,2
208	F1	207,4
209	F1	208,4
210	F1	216,7
211	F1	175,1
212	F1	164,5
213	F1	159,2
214	F1	174,9
215	F1	166
216	F1	134,9
217	F1	147,6
218	F1	190,9
219	F1	211,3
220	F1	205,6
221	F1	196,9
222	F1	168,8
223	F1	182,4

224	F1	265,4
225	F1	159
226	F1	157,1
227	F1	178,8
228	F1	183,4
229	F1	145,4
230	F1	204,2
231	F1	155,3
232	F1	236,5
233	F1	143,2
234	F1	256,8
235	F1	136,9
236	F1	162,1
237	F1	153
238	F1	136,2
239	F1	204,3
240	F1	195,6
241	F1	265,4
242	F1	133,1
243	F1	125,9
244	F1	133
245	F1	170,1
246	F1	106,9
247	F1	212,2
248	F1	301,8
249	F1	142,6
250	F1	240,7
251	F1	174,7
252	F1	119,7
253	F1	246,3
254	F1	163,2
255	F1	161,8
256	F1	181,4
257	F1	195,6
258	F1	167,5
259	F1	133,9
260	F1	188,7
261	F1	214,5
262	F1	98,7
263	F1	83,3
264	F1	130,6
265	F1	344,7
266	F1	136,2
267	F1	110,9
268	F1	158

269	F1	188
270	F1	158,3
271	F1	200,3
272	F1	225,5
273	F1	188
274	F1	152,1
275	F1	223,5
276	F1	241,2
277	F1	178,4
278	F1	137,2
279	F1	205,1
280	F1	277,8
281	F1	188
282	F1	283,2
283	F1	174,5
284	F1	122,8
285	F1	116,7
286	F1	289,3
287	F1	137,2
288	F1	253,6
289	F1	175,9
290	F1	146,3
291	F1	223
292	F1	175
293	F1	159,1
294	F1	130,8
295	F1	216,8
296	F1	155,4
297	F1	184,7
298	F1	243,6
299	F1	127,9
300	F1	269,7
301	F1	199,1
302	F1	229,9
303	F1	180
304	F1	225,1
305	F1	291,9
306	F1	154,6
307	F1	262,9
308	F1	200,1
309	F1	298,6
310	F1	200
311	F1	250,4
312	F1	191,7
313	F1	193,7

314	F1	215,3
315	F1	131,5
316	F1	237,7
317	F1	237,3
318	F1	217,3
319	F1	187,6
320	F1	289,9
321	F1	188,8
322	F1	157,5
323	F1	203,2
324	F1	168,5
325	F1	262,1
326	F1	120,4
327	F1	153,7
328	F1	210,2
329	F1	193,8
330	F1	238,3
331	F1	193,1
332	F1	163,6
333	F1	131,9
334	F1	107,1
335	F1	218,9
336	F1	131,3
337	F1	197,3
338	F1	156,1
339	F1	119,8
340	F1	176,4
341	F1	196,6
342	F1	351
343	F1	175,3
344	F1	124,4
345	F1	291,9
346	F1	155,2
347	F1	195,1
348	F1	199,5
349	F1	185,8
350	F1	193,2
351	F1	170,4
352	F1	146,2
353	F1	148,9
354	F1	334,9
355	F1	189,2
356	F1	172
357	F1	184,1
358	F1	181,2

359	F1	104,3
360	F1	149,9
361	F1	178,5
362	F1	197,4
363	F1	207,2
364	F1	153,6
365	F1	203,5
366	F1	141,8
367	F1	199
368	F1	148,1
369	F1	154,5
370	F1	103,8
371	F1	106
372	F1	183,1
373	F1	116,9
374	F1	116,2
375	F1	155,1
376	F1	177,8
377	F1	137,3
378	F1	126
379	F1	158,2
380	F1	174,3
381	F1	153,8
382	F1	125,3
383	F1	209,1
384	F1	100,1
385	F1	194,7
386	F1	149,1
387	F1	121,1
388	F1	162,4
389	F1	157,3
390	F1	118,1
391	F1	158,8
392	F1	113,3
393	F1	147
394	F1	159,5
395	F1	100,4
396	F1	128,3
397	F1	121,7
398	F1	153
399	F1	156,1
400	F1	129,9
401	F1	160
402	F1	192,6
403	F1	146,5

404	F1	176,7
405	F1	144,2
406	F1	158,3
407	F1	120,1
408	F1	154,5
409	F1	180
410	F1	135,7
411	F1	174,6
412	F1	175,2
413	F1	153,4
414	F1	137,7
415	F1	144,6
416	F1	117,4
417	F1	124,7
418	F1	133,3
419	F1	147,9
420	F1	132
421	F1	168,5
422	F1	198,4
423	F1	136,4
424	F1	192,6
425	F1	171
426	F1	124,7
427	F1	182,1
428	F1	165,2
429	F1	109,3
430	F1	194,9
431	F1	163,7
432	F1	130,8
433	F1	152,7
434	F1	133,2
435	F1	121,8
436	F1	162
437	F1	121,3
438	F1	172,1
439	F1	147,7
440	F1	152,1
441	F1	155
442	F1	118,6
443	F1	86,3
444	F1	149,6
445	F1	107,2
446	F1	142
447	F1	144,9
448	F1	130,4

449	F1	113,5
450	F1	123,1
451	F1	111,4
452	F1	152,6
453	F1	157,2
454	F1	111,8
455	F1	132,5
456	F1	108,4
457	F1	133,6
458	F1	132,8
459	F1	197,7
460	F1	142,5
461	F1	144,7
462	F1	198,6
463	F1	141
464	F1	119,7
465	F1	124
466	F1	145,6
467	F1	139,9
468	F1	119,4
469	F1	169,8
470	F1	130,2
471	F1	166,3
472	F2	421,3
473	F2	319,2
474	F2	383,5
475	F2	476,7
476	F2	456,5
477	F2	399,7
478	F2	393,1
479	F2	422,1
480	F2	467,4
481	F2	458,2
482	F2	385,1
483	F2	371,1
484	F2	428,4
485	F2	425
486	F2	331,5
487	F2	316,2
488	F2	396,3
489	F2	334
490	F2	395,3
491	F2	350,9
492	F2	422,3
493	F2	300,1

494	F2	422,9
495	F2	386,8
496	F2	345,5
497	F2	434,3
498	F2	346,2
499	F2	280,6
500	F2	390,8
501	F2	354,3
502	F2	298,8
503	F2	302,3
504	F2	364,1
505	F2	315,7
506	F2	288,3
507	F2	367,4
508	F2	253
509	F2	262,1
510	F2	315,1
511	F2	257,5
512	F2	267,9
513	F2	314,6
514	F2	228,4
515	F2	248,2
516	F2	391,7
517	F2	277,5
518	F2	229,1
519	F2	263
520	F2	270,5
521	F2	180,5
522	F2	234,4
523	F2	269,4
524	F2	251
525	F2	175,4
526	F2	277,8
527	F2	195,3
528	F2	242,3
529	F2	212,1
530	F2	234,5
531	F2	202,3
532	F2	239,7
533	F2	229,3
534	F2	229,6
535	F2	239,1
536	F2	230,1
537	F2	260,3
538	F2	228,8

539	F2	201,3
540	F2	242,1
541	F2	215,1
542	F2	272,4
543	F2	237,2
544	F2	256,2
545	F2	220,4
546	F2	225,1
547	F2	197,1
548	F2	219,4
549	F2	266
550	F2	229,5
551	F2	244,4
552	F2	253,8
553	F2	269,4
554	F2	219,6
555	F2	259,1
556	F2	253,6
557	F2	273,8
558	F2	235,9
559	F2	235,7
560	F2	266
561	F2	249
562	F2	244,4
563	F2	263,4
564	F2	248,7
565	F2	245,8
566	F2	238,1
567	F2	250,2
568	F2	235,6
569	F2	254,7
570	F2	232,9
571	F2	255
572	F2	255
573	F2	256,3
574	F2	283,5
575	F2	256,7
576	F2	297
577	F2	240,8
578	F2	271,2
579	F2	256,3
580	F2	235,1
581	F2	258,9
582	F2	260,6
583	F2	275,5

584	F2	269,9
585	F2	258,3
586	F2	236,1
587	F2	264,6
588	F2	266,8
589	F2	261,7
590	F2	266,5
591	F2	246,7
592	F2	256,5
593	F2	273,1
594	F2	271
595	F2	259,4
596	F2	252
597	F2	283,2
598	F2	296,9
599	F2	262,1
600	F2	262,4
601	F2	288,7
602	F2	245,5
603	F2	254
604	F2	275,3
605	F2	266,1
606	F2	283,2
607	F2	277,3
608	F2	262,5
609	F2	268,4
610	F2	252,3
611	F2	288,8
612	F2	273,9
613	F2	313,1
614	F2	348,1
615	F2	257,9
616	F2	284,1
617	F2	205,4
618	F2	267,7
619	F2	276,5
620	F2	279,2
621	F2	299,5
622	F2	283,3
623	F2	291,9
624	F2	271,5
625	F2	279,1
626	F2	289,2
627	F2	254,2
628	F2	263,9

629	F2	289,3
630	F2	290,9
631	F2	301,5
632	F2	293,8
633	F2	267,1
634	F2	294,5
635	F2	276,6
636	F2	233,8
637	F2	292,3
638	F2	245
639	F2	257,2
640	F2	290,6
641	F2	296,1
642	F2	292,4
643	F2	196,4
644	F2	297,4
645	F2	302,1
646	F2	304,7
647	F2	364,9
648	F2	288,4
649	F2	301,6
650	F2	272
651	F2	294,5
652	F2	282,6
653	F2	274,8
654	F2	305,8
655	F2	297,8
656	F2	280,5
657	F2	317,8
658	F2	288,5
659	F2	277,7
660	F2	285,9
661	F2	260
662	F2	298,5
663	F2	287,6
664	F2	270,6
665	F2	279,3
666	F2	294,1
667	F2	279
668	F2	293,4
669	F2	338,9
670	F2	259,9
671	F2	207,6
672	F2	277
673	F2	281,7

674	F2	293,7
675	F2	305,2
676	F2	288,7
677	F2	241
678	F2	287,1
679	F2	252,5
680	F2	290,2
681	F2	280,7
682	F2	274,5
683	F2	288,1
684	F2	282,3
685	F2	297,1
686	F2	273
687	F2	273,4
688	F2	277,5
689	F2	283,7
690	F2	286,8
691	F2	293,5
692	F2	214,9
693	F2	277,1
694	F2	296,5
695	F2	368,5
696	F2	276,8
697	F2	284,6
698	F2	275,7
699	F2	268,8
700	F2	295
701	F2	299,7
702	F2	264,6
703	F2	294,2
704	F2	288,4
705	F2	241
706	F2	279,3
707	F2	361,2
708	F2	253
709	F2	274,9
710	F2	287,2
711	F2	265,4
712	F2	291,4
713	F2	286,7
714	F2	316,4
715	F2	279,9
716	F2	278,4
717	F2	275,3
718	F2	286,7

719	F2	295,6
720	F2	276,7
721	F2	284,4
722	F2	297,1
723	F2	246,3
724	F2	285,1
725	F2	294,5
726	F2	291,2
727	F2	291,2
728	F2	297,8
729	F2	286,4
730	F2	274,1
731	F2	278
732	F2	289,1
733	F2	271,1
734	F2	290,6
735	F2	287,7
736	F2	265,7
737	F2	261,2
738	F2	276,8
739	F2	279
740	F2	284,8
741	F2	273,1
742	F2	281,7
743	F2	296,9
744	F2	278,7
745	F2	322,5
746	F2	253,7
747	F2	270,7
748	F2	289,4
749	F2	286,4
750	F2	260,5
751	F2	286,4
752	F2	297,8
753	F2	267,3
754	F2	260,9
755	F2	277,8
756	F2	248
757	F2	347,4
758	F2	269,1
759	F2	201,7
760	F2	274,5
761	F2	283,2
762	F2	285,6
763	F2	263,3

764	F2	269,8
765	F2	315,6
766	F2	295
767	F2	291,9
768	F2	269,7
769	F2	272,1
770	F2	294,2
771	F2	290,5
772	F2	318,8
773	F2	273,7
774	F2	278,2
775	F2	268,9
776	F2	295,7
777	F2	277
778	F2	272,6
779	F2	289,2
780	F2	276,7
781	F2	309,6
782	F2	264,1
783	F2	306,8
784	F2	276,2
785	F2	274,1
786	F2	286,1
787	F2	282,2
788	F2	279,6
789	F2	285,2
790	F2	315,4
791	F2	272,8
792	F2	283,8
793	F2	304,5
794	F2	284,9
795	F2	359,5
796	F2	309,2
797	F2	277,9
798	F2	238
799	F2	251,8
800	F2	240,5
801	F2	233,5
802	F2	249,3
803	F2	245,6
804	F2	230,7
805	F2	205,6
806	F2	212,3
807	F2	215
808	F2	220,5

809	F2	168,4
810	F2	206,9
811	F2	206,2
812	F2	181
813	F2	200,1
814	F2	199,5
815	F2	192,2
816	F2	162,5
817	F2	187,5
818	F2	208,6
819	F2	179,4
820	F2	173,5
821	F2	196,4
822	F2	163
823	F2	153,2
824	F2	190
825	F2	168,4
826	F2	120,8
827	F2	177,5
828	F2	179,1
829	F2	197,4
830	F2	117,4
831	F2	202,2
832	F2	154,1
833	F2	132,7
834	F2	191,1
835	F2	165,7
836	F2	131,6
837	F2	162,9
838	F2	151,8
839	F2	161
840	F2	175
841	F2	182,3
842	F2	147,1
843	F2	173,5
844	F2	176,8
845	F2	178,9
846	F2	170,7
847	F2	195,3
848	F2	150,1
849	F2	183,6
850	F2	182,2
851	F2	174,1
852	F2	185,9
853	F2	197,4

854	F2	168,9
855	F2	180,8
856	F2	185,2
857	F2	179,5
858	F2	195,8
859	F2	197,4
860	F2	191,5
861	F2	184,1
862	F2	194,3
863	F2	171,5
864	F2	184,9
865	F2	192,9
866	F2	181
867	F2	184,8
868	F2	194,4
869	F2	185,4
870	F2	200,1
871	F2	219
872	F2	187,5
873	F2	194,1
874	F2	208
875	F2	176,4
876	F2	202,1
877	F2	214,9
878	F2	194,8
879	F2	200
880	F2	214
881	F2	195
882	F2	211,1
883	F2	223,4
884	F2	199,5
885	F2	209,1
886	F2	225,5
887	F2	218,2
888	F2	215,6
889	F2	221,1
890	F2	212,9
891	F2	226,7
892	F2	222
893	F2	212,9
894	F2	208,2
895	F2	213
896	F2	209,4
897	F2	221,1
898	F2	234,9

899	F2	213,1
900	F2	234,9
901	F2	215,6
902	F2	212,9
903	F2	218,8
904	F2	232,1
905	F2	220
906	F2	232
907	F2	228,3
908	F2	227,6
909	F2	231,5
910	F2	226,5
911	F2	219
912	F2	229,7
913	F2	229,6
914	F2	222,5
915	F2	230,9
916	F2	223,6
917	F2	227,6
918	F2	229,8
919	F2	242
920	F2	231,9
921	F2	233,4
922	F2	222,9
923	F2	227,2
924	F2	233,7
925	F2	229,2
926	F2	220,4
927	F2	235,9
928	F2	225,6
929	F2	228,2
930	F2	235,2
931	F2	230,7
932	F2	228,3
933	F2	234
934	F2	233
935	F2	245,6
936	F2	242,5
937	F2	233,8
938	F2	230,5
939	F2	244,6
940	F2	235,9
941	F2	224,4
942	F2	248,8
943	M1	410,2

944	M1	326,8
945	M1	344
946	M1	401,7
947	M1	397,5
948	M1	318,3
949	M1	318,6
950	M1	411,6
951	M1	347,4
952	M1	251,5
953	M1	443,5
954	M1	447,4
955	M1	382,5
956	M1	370,1
957	M1	435,9
958	M1	405,8
959	M1	385,3
960	M1	374,8
961	M1	408,8
962	M1	352,6
963	M1	343
964	M1	395,1
965	M1	406,8
966	M1	198
967	M1	265,4
968	M1	450,7
969	M1	402,2
970	M1	359
971	M1	255,9
972	M1	432
973	M1	309,3
974	M1	257,7
975	M1	389,5
976	M1	414,8
977	M1	259,5
978	M1	389,4
979	M1	294,6
980	M1	305,3
981	M1	326,8
982	M1	377,5
983	M1	404,9
984	M1	276
985	M1	355,6
986	M1	316,2
987	M1	255,7
988	M1	253,9

989	M1	275,8
990	M1	292
991	M1	306,7
992	M1	319,5
993	M1	345,8
994	M1	193,8
995	M1	338,2
996	M1	296,1
997	M1	236,6
998	M1	259,2
999	M1	306,2
1000	M1	210
1001	M1	316,9
1002	M1	312,7
1003	M1	238,6
1004	M1	267
1005	M1	289,2
1006	M1	211,1
1007	M1	192,9
1008	M1	245,1
1009	M1	267,9
1010	M1	241,4
1011	M1	223
1012	M1	279,4
1013	M1	239,4
1014	M1	218,1
1015	M1	265,3
1016	M1	224
1017	M1	250,7
1018	M1	292
1019	M1	251,1
1020	M1	201,7
1021	M1	252,5
1022	M1	290,6
1023	M1	201,9
1024	M1	232,6
1025	M1	249,3
1026	M1	213,9
1027	M1	248,7
1028	M1	286,8
1029	M1	238,2
1030	M1	184,2
1031	M1	292,3
1032	M1	318
1033	M1	195

1034	M1	257,1
1035	M1	292
1036	M1	248,7
1037	M1	255,1
1038	M1	299,6
1039	M1	219,3
1040	M1	227,2
1041	M1	225,7
1042	M1	164,7
1043	M1	273,4
1044	M1	247,3
1045	M1	199,7
1046	M1	222,1
1047	M1	236
1048	M1	242,2
1049	M1	227,9
1050	M1	226,8
1051	M1	197,6
1052	M1	224
1053	M1	218,6
1054	M1	243,2
1055	M1	237,1
1056	M1	283,9
1057	M1	216,4
1058	M1	247,4
1059	M1	229,4
1060	M1	211,6
1061	M1	233,5
1062	M1	254
1063	M1	268,3
1064	M1	272,4
1065	M1	232,9
1066	M1	197
1067	M1	246,2
1068	M1	195,6
1069	M1	213,5
1070	M1	245,8
1071	M1	238
1072	M1	202,4
1073	M1	252,3
1074	M1	249,5
1075	M1	181,6
1076	M1	200,4
1077	M1	203,2
1078	M1	189

1079	M1	189,6
1080	M1	218,2
1081	M1	192,9
1082	M1	174,7
1083	M1	221,5
1084	M1	175,9
1085	M1	216
1086	M1	235,2
1087	M1	167,5
1088	M1	174,7
1089	M1	195,9
1090	M1	185,8
1091	M1	203,7
1092	M1	207
1093	M1	162,6
1094	M1	237,2
1095	M1	161
1096	M1	200,2
1097	M1	195
1098	M1	245,5
1099	M1	171
1100	M1	212,7
1101	M1	236
1102	M1	182
1103	M1	214
1104	M1	191,8
1105	M1	161,7
1106	M1	247,2
1107	M1	194,4
1108	M1	208
1109	M1	186,7
1110	M1	240,8
1111	M1	161,3
1112	M1	180
1113	M1	257,1
1114	M1	187,2
1115	M1	212,8
1116	M1	219,8
1117	M1	185
1118	M1	170,8
1119	M1	262,7
1120	M1	204,2
1121	M1	186,9
1122	M1	209,7
1123	M1	230,3

1124	M1	209,4
1125	M1	230,3
1126	M1	245,1
1127	M1	224,7
1128	M1	272,5
1129	M1	176
1130	M1	175,4
1131	M1	219,3
1132	M1	264,6
1133	M1	177,9
1134	M1	218
1135	M1	251,2
1136	M1	146,8
1137	M1	190,8
1138	M1	264,1
1139	M1	248,5
1140	M1	302,1
1141	M1	231,7
1142	M1	237,2
1143	M1	198,3
1144	M1	264,6
1145	M1	197,5
1146	M1	239,9
1147	M1	277,6
1148	M1	218,4
1149	M1	244,5
1150	M1	327,3
1151	M1	207
1152	M1	240
1153	M1	216,5
1154	M1	259,2
1155	M1	159,5
1156	M1	245,2
1157	M1	269
1158	M1	202,9
1159	M1	259,3
1160	M1	296
1161	M1	220,9
1162	M1	239,7
1163	M1	282,5
1164	M1	231,7
1165	M1	206,7
1166	M1	264,5
1167	M1	215,9
1168	M1	254,5

1169	M1	240,9
1170	M1	235
1171	M1	235,5
1172	M1	289,6
1173	M1	261
1174	M1	166,9
1175	M1	258,6
1176	M1	181
1177	M1	219,8
1178	M1	273,3
1179	M1	262,1
1180	M1	148,9
1181	M1	249,7
1182	M1	240,2
1183	M1	229,8
1184	M1	227,3
1185	M1	295,2
1186	M1	213,7
1187	M1	261,9
1188	M1	259
1189	M1	254,7
1190	M1	249,2
1191	M1	281,4
1192	M1	199,1
1193	M1	256,4
1194	M1	246
1195	M1	188,7
1196	M1	231
1197	M1	335,6
1198	M1	206
1199	M1	249
1200	M1	234,7
1201	M1	216
1202	M1	288,5
1203	M1	270
1204	M1	222,3
1205	M1	212,3
1206	M1	375,4
1207	M1	184,2
1208	M1	246,3
1209	M1	291,4
1210	M1	181,9
1211	M1	233,2
1212	M1	275,6
1213	M1	286,4

1214	M1	260,7
1215	M1	330,6
1216	M1	303,1
1217	M1	263,9
1218	M1	224,4
1219	M1	302,4
1220	M1	275,6
1221	M1	252,8
1222	M1	262,4
1223	M1	214,9
1224	M1	289,9
1225	M1	319,7
1226	M1	212,8
1227	M1	218
1228	M1	293,3
1229	M1	294,5
1230	M1	217,3
1231	M1	252,5
1232	M1	243,2
1233	M1	231,9
1234	M1	354,2
1235	M1	191,9
1236	M1	277
1237	M1	286,6
1238	M1	262,7
1239	M1	203,2
1240	M1	308,7
1241	M1	171
1242	M1	241,4
1243	M1	303,8
1244	M1	250,6
1245	M1	273,9
1246	M1	258,4
1247	M1	275,7
1248	M1	205,2
1249	M1	233,8
1250	M1	271,8
1251	M1	194,5
1252	M1	223,9
1253	M1	217,6
1254	M1	360,6
1255	M1	341,3
1256	M1	326,4
1257	M1	391,8
1258	M1	240,8

1259	M1	344,9
1260	M1	339,3
1261	M1	323,6
1262	M1	305,5
1263	M1	244,6
1264	M1	318,2
1265	M1	308,2
1266	M1	367
1267	M1	339
1268	M1	339,5
1269	M1	308,3
1270	M1	270,8
1271	M1	345,9
1272	M1	251,1
1273	M1	272,9
1274	M1	323,6
1275	M1	246
1276	M1	283
1277	M1	191,6
1278	M1	307,8
1279	M1	240,6
1280	M1	320,5
1281	M1	231
1282	M1	335,6
1283	M1	206
1284	M1	249
1285	M1	234,7
1286	M1	216
1287	M1	288,5
1288	M1	270
1289	M1	222,3
1290	M1	212,3
1291	M1	375,4
1292	M1	184,2
1293	M1	246,3
1294	M1	291,4
1295	M1	181,9
1296	M1	233,2
1297	M1	275,6
1298	M1	231
1299	M1	335,6
1300	M1	206
1301	M1	249
1302	M1	234,7
1303	M1	216

1304	M1	288,5
1305	M1	270
1306	M1	222,3
1307	M1	212,3
1308	M1	375,4
1309	M1	184,2
1310	M1	246,3
1311	M1	291,4
1312	M1	181,9
1313	M1	233,2
1314	M1	275,6
1315	M1	231
1316	M1	335,6
1317	M1	206
1318	M1	249
1319	M1	234,7
1320	M1	216
1321	M1	288,5
1322	M1	270
1323	M1	222,3
1324	M1	212,3
1325	M1	375,4
1326	M1	184,2
1327	M1	246,3
1328	M1	291,4
1329	M1	181,9
1330	M1	233,2
1331	M1	275,6
1332	M1	231
1333	M1	335,6
1334	M1	206
1335	M1	249
1336	M1	234,7
1337	M1	216
1338	M1	288,5
1339	M1	270
1340	M1	222,3
1341	M1	212,3
1342	M1	375,4
1343	M1	184,2
1344	M1	246,3
1345	M1	291,4
1346	M1	181,9
1347	M1	233,2
1348	M1	275,6

1349	M1	251,1
1350	M1	201,7
1351	M1	252,5
1352	M1	290,6
1353	M1	201,9
1354	M1	232,6
1355	M1	249,3
1356	M1	213,9
1357	M1	248,7
1358	M1	286,8
1359	M1	238,2
1360	M1	184,2
1361	M1	292,3
1362	M1	318
1363	M1	195
1364	M1	257,1
1365	M1	292
1366	M1	248,7
1367	M1	255,1
1368	M1	299,6
1369	M1	219,3
1370	M1	227,2
1371	M1	225,7
1372	M1	164,7
1373	M1	273,4
1374	M1	247,3
1375	M1	199,7
1376	M1	222,1
1377	M1	236
1378	M1	242,2
1379	M1	227,9
1380	M1	226,8
1381	M1	197,6
1382	M1	224
1383	M1	218,6
1384	M1	243,2
1385	M1	237,1
1386	M1	283,9
1387	M1	216,4
1388	M1	247,4
1389	M1	229,4
1390	M1	211,6
1391	M1	233,5
1392	M1	254
1393	M1	268,3

1394	M1	272,4
1395	M1	232,9
1396	M1	197
1397	M1	246,2
1398	M1	195,6
1399	M1	213,5
1400	M1	245,8
1401	M1	238
1402	M1	202,4
1403	M1	252,3
1404	M1	249,5
1405	M1	181,6
1406	M1	200,4
1407	M1	203,2
1408	M1	189
1409	M1	189,6
1410	M1	218,2
1411	M1	192,9
1412	M1	174,7
1413	M2	353,5
1414	M2	358,9
1415	M2	305,8
1416	M2	362,8
1417	M2	294,4
1418	M2	351,3
1419	M2	378,1
1420	M2	394,6
1421	M2	337,4
1422	M2	317,8
1423	M2	370,3
1424	M2	347,3
1425	M2	158,7
1426	M2	341,3
1427	M2	361,5
1428	M2	440,7
1429	M2	415,2
1430	M2	382,4
1431	M2	363,3
1432	M2	389,5
1433	M2	367,6
1434	M2	438,5
1435	M2	334,6
1436	M2	433,1
1437	M2	380,6
1438	M2	420,7

1439	M2	367,2
1440	M2	407,3
1441	M2	400,4
1442	M2	310,1
1443	M2	379
1444	M2	345,5
1445	M2	383,7
1446	M2	364,1
1447	M2	273,5
1448	M2	356,4
1449	M2	389,7
1450	M2	312,4
1451	M2	307,3
1452	M2	321,1
1453	M2	227,4
1454	M2	182
1455	M2	305,4
1456	M2	321
1457	M2	284,9
1458	M2	400
1459	M2	266,3
1460	M2	272
1461	M2	297,1
1462	M2	283,7
1463	M2	230,2
1464	M2	316,1
1465	M2	263,3
1466	M2	188,2
1467	M2	333,7
1468	M2	166
1469	M2	214,9
1470	M2	361,8
1471	M2	198,8
1472	M2	306,5
1473	M2	258,4
1474	M2	177,8
1475	M2	194,9
1476	M2	258,6
1477	M2	167,2
1478	M2	229,1
1479	M2	211,8
1480	M2	198,6
1481	M2	256,2
1482	M2	192,3
1483	M2	190,3

1484	M2	218,9
1485	M2	179,8
1486	M2	232
1487	M2	221,4
1488	M2	222,8
1489	M2	184,4
1490	M2	277,9
1491	M2	142
1492	M2	242,4
1493	M2	227,9
1494	M2	163,7
1495	M2	230
1496	M2	200,3
1497	M2	185,6
1498	M2	186,7
1499	M2	180,4
1500	M2	213,3
1501	M2	218,6
1502	M2	169,6
1503	M2	184,9
1504	M2	211
1505	M2	164
1506	M2	187,2
1507	M2	180,7
1508	M2	225,9
1509	M2	233,7
1510	M2	157,4
1511	M2	202,6
1512	M2	202,4
1513	M2	188,9
1514	M2	237,4
1515	M2	178,4
1516	M2	210,9
1517	M2	217,8
1518	M2	206,6
1519	M2	234
1520	M2	215,2
1521	M2	196
1522	M2	272,8
1523	M2	205,5
1524	M2	205,1
1525	M2	231,9
1526	M2	219,5
1527	M2	140,2
1528	M2	172,2

1529	M2	203,2
1530	M2	169,7
1531	M2	199,3
1532	M2	234,9
1533	M2	217,1
1534	M2	186,9
1535	M2	241,1
1536	M2	182,9
1537	M2	174
1538	M2	186,9
1539	M2	217,4
1540	M2	213,2
1541	M2	183,6
1542	M2	226,8
1543	M2	238,8
1544	M2	183,7
1545	M2	248,1
1546	M2	180,2
1547	M2	188,7
1548	M2	174,6
1549	M2	172,9
1550	M2	197,8
1551	M2	176,8
1552	M2	184,3
1553	M2	221,2
1554	M2	191,2
1555	M2	212,4
1556	M2	186,6
1557	M2	205,7
1558	M2	230,1
1559	M2	200,6
1560	M2	225,2
1561	M2	230,3
1562	M2	160,7
1563	M2	222,6
1564	M2	189,7
1565	M2	245,2
1566	M2	159,1
1567	M2	180,3
1568	M2	246,1
1569	M2	209,9
1570	M2	240,2
1571	M2	242,3
1572	M2	231,2
1573	M2	239,6

1574	M2	196,2
1575	M2	204,4
1576	M2	191
1577	M2	201,5
1578	M2	223,9
1579	M2	180,1
1580	M2	245,6
1581	M2	227,4
1582	M2	204
1583	M2	259,5
1584	M2	179,1
1585	M2	204,6
1586	M2	143,8
1587	M2	204,9
1588	M2	223,9
1589	M2	225,7
1590	M2	228,9
1591	M2	180,6
1592	M2	212,5
1593	M2	181,1
1594	M2	153,6
1595	M2	245,7
1596	M2	207,7
1597	M2	218,1
1598	M2	202,6
1599	M2	151,6
1600	M2	250,4
1601	M2	225
1602	M2	265,4
1603	M2	214,2
1604	M2	171,5
1605	M2	90,9
1606	M2	131,1
1607	M2	252,2
1608	M2	188,3
1609	M2	129,3
1610	M2	187,1
1611	M2	175,7
1612	M2	204,8
1613	M2	161,3
1614	M2	235,5
1615	M2	217,8
1616	M2	164,4
1617	M2	235,4
1618	M2	201,1

1619	M2	128,9
1620	M2	192,3
1621	M2	142,1
1622	M2	194,2
1623	M2	171,7
1624	M2	160,5
1625	M2	236,7
1626	M2	194,4
1627	M2	232,7
1628	M2	173,5
1629	M2	162,6
1630	M2	217,6
1631	M2	150,6
1632	M2	226,7
1633	M2	201,5
1634	M2	189,3
1635	M2	177,2
1636	M2	185,2
1637	M2	163,9
1638	M2	181,9
1639	M2	205,7
1640	M2	245,1
1641	M2	161,4
1642	M2	160,9
1643	M2	160,6
1644	M2	195,9
1645	M2	200,9
1646	M2	160,2
1647	M2	219,2
1648	M2	147,6
1649	M2	198,3
1650	M2	233,3
1651	M2	106,4
1652	M2	258
1653	M2	211,2
1654	M2	153,2
1655	M2	233,8
1656	M2	193,9
1657	M2	211,4
1658	M2	133,2
1659	M2	203,5
1660	M2	189,7
1661	M2	178,6
1662	M2	141,7
1663	M2	175,8

1664	M2	192,5
1665	M2	163,6
1666	M2	193,7
1667	M2	218,7
1668	M2	148
1669	M2	268,2
1670	M2	176,3
1671	M2	128,4
1672	M2	226,3
1673	M2	151,4
1674	M2	191,8
1675	M2	207,1
1676	M2	108,3
1677	M2	199,9
1678	M2	194,6
1679	M2	169,2
1680	M2	258,3
1681	M2	145,3
1682	M2	156,1
1683	M2	200,5
1684	M2	191,1
1685	M2	183,3
1686	M2	249,3
1687	M2	253,8
1688	M2	160,1
1689	M2	238,2
1690	M2	220,7
1691	M2	172,6
1692	M2	137
1693	M2	97,8
1694	M2	194,6
1695	M2	95,6
1696	M2	137,4
1697	M2	203,2
1698	M2	120,5
1699	M2	187,7
1700	M2	211,6
1701	M2	120,4
1702	M2	281,4
1703	M2	81,8
1704	M2	283,8
1705	M2	229,9
1706	M2	223,8
1707	M2	222,5
1708	M2	102,4

1709	M2	150,2
1710	M2	136,4
1711	M2	203,7
1712	M2	237,4
1713	M2	68,6
1714	M2	283,5
1715	M2	206,2
1716	M2	122,3
1717	M2	174,3
1718	M2	155,9
1719	M2	227,7
1720	M2	162,5
1721	M2	96,7
1722	M2	223,3
1723	M2	147,4
1724	M2	229,1
1725	M2	145,7
1726	M2	121,1
1727	M2	254,6
1728	M2	356,2
1729	M2	128,8
1730	M2	189,1
1731	M2	180,4
1732	M2	219,7
1733	M2	145,3
1734	M2	184,1
1735	M2	205,3
1736	M2	139,6
1737	M2	196,1
1738	M2	145,2
1739	M2	200
1740	M2	215,6
1741	M2	131
1742	M2	239,4
1743	M2	169,4
1744	M2	188,6
1745	M2	150,4
1746	M2	115,6
1747	M2	222,6
1748	M2	206,7
1749	M2	131,3
1750	M2	194
1751	M2	175,8
1752	M2	197,9
1753	M2	312

1754	M2	218,4
1755	M2	299,3
1756	M2	285,2
1757	M2	256,6
1758	M2	302,6
1759	M2	244,6
1760	M2	264,4
1761	M2	281,5
1762	M2	261,9
1763	M2	220,5
1764	M2	300,9
1765	M2	268,7
1766	M2	197,1
1767	M2	196,8
1768	M2	273,2
1769	M2	273,3
1770	M2	246,6
1771	M2	226,4
1772	M2	211,6
1773	M2	224,8
1774	M2	172,5
1775	M2	211,8
1776	M2	194,4
1777	M2	137,7
1778	M2	230,1
1779	M2	200,5
1780	M2	217,5
1781	M2	224,5
1782	M2	163,7
1783	M2	234,5
1784	M2	202,4
1785	M2	199,5
1786	M2	215,5
1787	M2	209,9
1788	M2	215,6
1789	M2	229,4
1790	M2	184,4
1791	M2	217,4
1792	M2	232,6
1793	M2	209,9
1794	M2	160,5
1795	M2	208,8
1796	M2	214,8
1797	M2	250,8
1798	M2	180,1

1799	M2	253,4
1800	M2	174,1
1801	M2	219,9
1802	M2	243,1
1803	M2	223
1804	M2	205,6
1805	M2	184,1
1806	M2	227,9
1807	M2	276,5
1808	M2	224,2
1809	M2	321,2
1810	M2	343,1
1811	M2	349,2
1812	M2	329,4
1813	M2	382
1814	M2	382,9
1815	M2	333,7
1816	M2	325,9
1817	M2	314,6
1818	M2	369,1
1819	M2	316,3
1820	M2	342
1821	M2	286,9
1822	M2	370,8
1823	M2	318,4
1824	M2	292
1825	M2	314,5
1826	M2	232,8
1827	M2	302,1
1828	M2	342,3
1829	M2	328,5
1830	M2	338,3
1831	M2	325,9
1832	M2	301,5
1833	M2	304,1
1834	M2	365,2
1835	M2	299,3
1836	M2	394,3
1837	M2	252,5
1838	M2	321,5
1839	M2	304,4
1840	M2	301,9
1841	M2	373,2
1842	M2	375,5
1843	M2	295,4

1844	M2	342,7
1845	M2	159,1
1846	M2	263,7
1847	M2	308,8
1848	M2	204,5
1849	M2	256,5
1850	M2	240
1851	M2	193,1
1852	M2	233,3
1853	M2	153,9
1854	M2	211,9
1855	M2	186,9
1856	M2	212,2
1857	M2	190,1
1858	M2	93,7
1859	M2	274,3
1860	M2	76,5
1861	M2	131,1
1862	M2	193,3
1863	M2	245,3
1864	M2	157,8
1865	M2	235,9
1866	M2	170
1867	M2	223,7
1868	M2	202,7
1869	M2	144,6
1870	M2	247,2
1871	M2	199,3
1872	M2	163,1
1873	M2	189,3
1874	M2	277,6
1875	M2	285,5
1876	M2	194,9
1877	M2	158,2
1878	M2	246,9
1879	M2	227,1
1880	M2	159,1
1881	M2	133,7
1882	M2	195,1
