# Supplementary material for: Does a gender of Welwitschia mirabilis plants influence their photosynthetic activity?
Source: PLoS One. 2023 Sep 8;18(9):e0291122. doi: 10.1371/journal.pone.0291122 (PMC10490862; doi:10.1371/journal.pone.0291122)
Supplement: S4 Table — (PDF) [file pone.0291122.s004.pdf]

| Measurement number | Specimen number | Parameter $P_N$ |
|---|---|---|
| 1 | M1 | 2,78 |
| 2 | M1 | 2,94 |
| 3 | M1 | 2,04 |
| 4 | M1 | 1,73 |
| 5 | M1 | 1,82 |
| 6 | M1 | 1,65 |
| 7 | M1 | 2,84 |
| 8 | M1 | 1,34 |
| 9 | M1 | 1,75 |
| 10 | M1 | 2,44 |
| 11 | M1 | 1,23 |
| 12 | M1 | 1,73 |
| 13 | M1 | 2,62 |
| 14 | M1 | 1 |
| 15 | M1 | 1,79 |
| 16 | M1 | 2,23 |
| 17 | M1 | 1,19 |
| 18 | M1 | 1,45 |
| 19 | M1 | 2,57 |
| 20 | M1 | 0,98 |
| 21 | M1 | 1,26 |
| 22 | M1 | 2,14 |
| 23 | M1 | 1,75 |
| 24 | M1 | 2,47 |
| 25 | M1 | 1,33 |
| 26 | M1 | 1,15 |
| 27 | M1 | 2,24 |
| 28 | M1 | 0,49 |
| 29 | M1 | 1,28 |
| 30 | M1 | 2,3 |
| 31 | M1 | 1,33 |
| 32 | M1 | 1,48 |
| 33 | M1 | 1,03 |
| 34 | M1 | 1,24 |
| 35 | M1 | 2,49 |
| 36 | M1 | 1,17 |
| 37 | M1 | 0,75 |
| 38 | M1 | 2,33 |
| 39 | M1 | 1,01 |
| 40 | M1 | 1,54 |
| 41 | M1 | 1,37 |
| 42 | M1 | 0,78 |
| 43 | M1 | 0,8 |
| 44 | M1 | 1,65 |

| | | |
|---|---|---|
| 45 | M1 | 0,68 |
| 46 | M1 | 1,06 |
| 47 | M1 | 0,94 |
| 48 | M1 | 0,73 |
| 49 | M1 | 1,68 |
| 50 | M1 | 0,84 |
| 51 | M1 | 1,79 |
| 52 | M1 | 1,88 |
| 53 | M1 | 0,94 |
| 54 | M1 | 1,1 |
| 55 | M1 | 1,99 |
| 56 | M1 | 0,29 |
| 57 | M1 | 1,87 |
| 58 | M1 | 1,62 |
| 59 | M1 | 0,93 |
| 60 | M1 | 1,63 |
| 61 | M1 | 0,84 |
| 62 | M1 | 0,74 |
| 63 | M1 | 1,76 |
| 64 | M1 | 0,98 |
| 65 | M1 | 1,63 |
| 66 | M1 | 2,26 |
| 67 | M1 | 0,89 |
| 68 | M1 | 1,56 |
| 69 | M1 | 1,71 |
| 70 | M1 | 0,98 |
| 71 | M1 | 0,72 |
| 72 | M1 | 0,74 |
| 73 | M1 | 1,38 |
| 74 | M1 | 1,92 |
| 75 | M1 | 1,17 |
| 76 | M1 | 0,6 |
| 77 | M1 | 1,43 |
| 78 | M1 | 0,43 |
| 79 | M1 | 1,69 |
| 80 | M1 | 0,99 |
| 81 | M1 | 1,04 |
| 82 | M1 | 1,12 |
| 83 | M1 | 1,2 |
| 84 | M1 | 0,89 |
| 85 | M1 | 1,23 |
| 86 | M1 | 0,91 |
| 87 | M1 | 1,59 |
| 88 | M1 | 0,96 |
| 89 | M1 | 1,22 |

| | | |
|---|---|---|
| 90 | M1 | 1,97 |
| 91 | M1 | 0,58 |
| 92 | M1 | 1,6 |
| 93 | M1 | 1,92 |
| 94 | M1 | 0,35 |
| 95 | M1 | 1,47 |
| 96 | M1 | 1,72 |
| 97 | M1 | 0,95 |
| 98 | M1 | 0,69 |
| 99 | M1 | 1,6 |
| 100 | M1 | 0,56 |
| 101 | M1 | 1,33 |
| 102 | M1 | 0,66 |
| 103 | M1 | 0,99 |
| 104 | M1 | 1,36 |
| 105 | M1 | 0,99 |
| 106 | M1 | 0,84 |
| 107 | M1 | 1,69 |
| 108 | M1 | 0,56 |
| 109 | M1 | 1,38 |
| 110 | M1 | 1,42 |
| 111 | M1 | 1,13 |
| 112 | M1 | 1,36 |
| 113 | M1 | 1,39 |
| 114 | M1 | 0,96 |
| 115 | M1 | 1,46 |
| 116 | M1 | 0,8 |
| 117 | M1 | 1,73 |
| 118 | M1 | 1,97 |
| 119 | M1 | 0,39 |
| 120 | M1 | 1,96 |
| 121 | M1 | 2,2 |
| 122 | M1 | 0,21 |
| 123 | M1 | 1,21 |
| 124 | M1 | 2,2 |
| 125 | M1 | 0,57 |
| 126 | M1 | 1,12 |
| 127 | M1 | 1,85 |
| 128 | M1 | 1,07 |
| 129 | M1 | 1,77 |
| 130 | M1 | 1,12 |
| 131 | M1 | 0,94 |
| 132 | M1 | 2,38 |
| 133 | M1 | 0,57 |
| 134 | M1 | 0,97 |

| | | |
|---|---|---|
| 135 | M1 | 1,33 |
| 136 | M1 | 0,46 |
| 137 | M1 | 1,56 |
| 138 | M1 | 2,27 |
| 139 | M1 | 0,69 |
| 140 | M1 | 1,74 |
| 141 | M1 | 0,82 |
| 142 | M1 | 1,55 |
| 143 | M1 | 1,41 |
| 144 | M1 | 1 |
| 145 | M1 | 2,12 |
| 146 | M1 | 1,95 |
| 147 | M1 | 1,24 |
| 148 | M1 | 1,02 |
| 149 | M1 | 1,4 |
| 150 | M1 | 0,63 |
| 151 | M1 | 1,19 |
| 152 | M1 | 2,08 |
| 153 | M1 | 0,87 |
| 154 | M1 | 1,21 |
| 155 | M1 | 2,03 |
| 156 | M1 | 1,73 |
| 157 | M1 | 2,39 |
| 158 | M1 | 1,6 |
| 159 | M1 | 1,09 |
| 160 | M1 | 2,7 |
| 161 | M1 | 1,53 |
| 162 | M1 | 1,56 |
| 163 | M1 | 2,9 |
| 164 | M1 | 0,64 |
| 165 | M1 | 1,57 |
| 166 | M1 | 2,55 |
| 167 | M1 | 1,09 |
| 168 | M1 | 2,28 |
| 169 | M1 | 0,94 |
| 170 | M1 | 1,45 |
| 171 | M1 | 2,25 |
| 172 | M1 | 1,09 |
| 173 | M1 | 2,41 |
| 174 | M1 | 2,43 |
| 175 | M1 | 1,11 |
| 176 | M1 | 1,24 |
| 177 | M1 | 2,2 |
| 178 | M1 | 1,79 |
| 179 | M1 | 1,05 |

| | | |
|---|---|---|
| 180 | M1 | 2,38 |
| 181 | M1 | 0,55 |
| 182 | M1 | 2,07 |
| 183 | M1 | 1,98 |
| 184 | M1 | 1,47 |
| 185 | M1 | 2 |
| 186 | M1 | 1,06 |
| 187 | M1 | 1,26 |
| 188 | M1 | 2,26 |
| 189 | M1 | 1,49 |
| 190 | M1 | 2,27 |
| 191 | M1 | 2,59 |
| 192 | M1 | 1,48 |
| 193 | M1 | 1,29 |
| 194 | M1 | 2,92 |
| 195 | M1 | 1,46 |
| 196 | M1 | 1,69 |
| 197 | M1 | 2,13 |
| 198 | M1 | 1,55 |
| 199 | M1 | 1,97 |
| 200 | M1 | 1,7 |
| 201 | M1 | 1,57 |
| 202 | M1 | 2,22 |
| 203 | M1 | 1,55 |
| 204 | M1 | 2,46 |
| 205 | M1 | 2,55 |
| 206 | M1 | 1,19 |
| 207 | M1 | 1,59 |
| 208 | M1 | 1,82 |
| 209 | M1 | 0,97 |
| 210 | M1 | 2,27 |
| 211 | M1 | 2,45 |
| 212 | M1 | 1,28 |
| 213 | M1 | 1,95 |
| 214 | M1 | 1,78 |
| 215 | M1 | 1,21 |
| 216 | M1 | 2,18 |
| 217 | M1 | 1,22 |
| 218 | M1 | 2,23 |
| 219 | M1 | 2,42 |
| 220 | M1 | 1,07 |
| 221 | M1 | 1,51 |
| 222 | M1 | 2,92 |
| 223 | M1 | 1,74 |
| 224 | M1 | 1,49 |

| | | |
|---|---|---|
| 225 | M1 | 1,72 |
| 226 | M1 | 1,33 |
| 227 | M1 | 1,88 |
| 228 | M1 | 1,86 |
| 229 | M1 | 2,35 |
| 230 | M1 | 2,53 |
| 231 | M1 | 1,42 |
| 232 | M1 | 2,47 |
| 233 | M1 | 2,96 |
| 234 | M1 | 0,71 |
| 235 | M1 | 2,51 |
| 236 | M1 | 2,23 |
| 237 | M1 | 1,53 |
| 238 | M1 | 2,14 |
| 239 | M1 | 2,2 |
| 240 | F2 | 1,34 |
| 241 | F2 | 1 |
| 242 | F2 | 0,8 |
| 243 | F2 | 0,72 |
| 244 | F2 | 0,96 |
| 245 | F2 | 2,34 |
| 246 | F2 | 1,35 |
| 247 | F2 | 1,62 |
| 248 | F2 | 0,56 |
| 249 | F2 | 0,65 |
| 250 | F2 | 2,16 |
| 251 | F2 | 0,55 |
| 252 | F2 | 0,63 |
| 253 | F2 | 0,92 |
| 254 | F2 | 1,14 |
| 255 | F2 | 0,79 |
| 256 | F2 | 1,96 |
| 257 | F2 | 0,9 |
| 258 | F2 | 0,28 |
| 259 | F2 | 1,16 |
| 260 | F2 | 1,19 |
| 261 | F2 | 0,41 |
| 262 | F2 | 0,92 |
| 263 | F2 | 1,84 |
| 264 | F2 | 1,29 |
| 265 | F2 | 1,77 |
| 266 | F2 | 2,89 |
| 267 | F2 | 1,09 |
| 268 | F2 | 2,65 |
| 269 | F2 | 3,39 |

| | | |
|---|---|---|
| 270 | F2 | 1,33 |
| 271 | F2 | 1,93 |
| 272 | F2 | 2,67 |
| 273 | F2 | 1,21 |
| 274 | F2 | 1,7 |
| 275 | F2 | 3,24 |
| 276 | F2 | 1,19 |
| 277 | F2 | 2,25 |
| 278 | F2 | 3,26 |
| 279 | F2 | 1,09 |
| 280 | F2 | 2,12 |
| 281 | F2 | 4,16 |
| 282 | F2 | 1,52 |
| 283 | F2 | 2,7 |
| 284 | F2 | 2,69 |
| 285 | F2 | 2,16 |
| 286 | F2 | 1,83 |
| 287 | F2 | 2,35 |
| 288 | F2 | 1,85 |
| 289 | F2 | 2,59 |
| 290 | F2 | 3,29 |
| 291 | F2 | 3,04 |
| 292 | F2 | 3,6 |
| 293 | F2 | 3,06 |
| 294 | F2 | 3,52 |
| 295 | F2 | 4,35 |
| 296 | F2 | 3,55 |
| 297 | F2 | 2,25 |
| 298 | F2 | 3,19 |
| 299 | F2 | 5,02 |
| 300 | F2 | 2,37 |
| 301 | F2 | 3,2 |
| 302 | F2 | 3,37 |
| 303 | F2 | 3,25 |
| 304 | F2 | 3,26 |
| 305 | F2 | 3,95 |
| 306 | F2 | 3,08 |
| 307 | F2 | 3,46 |
| 308 | F2 | 3,54 |
| 309 | F2 | 3,68 |
| 310 | F2 | 4,36 |
| 311 | F2 | 4,14 |
| 312 | F2 | 3,01 |
| 313 | F2 | 5,34 |
| 314 | F2 | 5,21 |

| | | |
|---|---|---|
| 315 | F2 | 2,8 |
| 316 | F2 | 3,59 |
| 317 | F2 | 4,39 |
| 318 | F2 | 3,42 |
| 319 | F2 | 3,68 |
| 320 | F2 | 4,26 |
| 321 | F2 | 4 |
| 322 | F2 | 5,08 |
| 323 | F2 | 4,4 |
| 324 | F2 | 3,19 |
| 325 | F2 | 5,19 |
| 326 | F2 | 4,47 |
| 327 | F2 | 4,22 |
| 328 | F2 | 3,99 |
| 329 | F2 | 4,26 |
| 330 | F2 | 4,89 |
| 331 | F2 | 5,27 |
| 332 | F2 | 4,25 |
| 333 | F2 | 4,39 |
| 334 | F2 | 4,08 |
| 335 | F2 | 3,63 |
| 336 | F2 | 4,07 |
| 337 | F2 | 5,59 |
| 338 | F2 | 4,82 |
| 339 | F2 | 4,39 |
| 340 | F2 | 5,19 |
| 341 | F2 | 3,62 |
| 342 | F2 | 4,02 |
| 343 | F2 | 6,04 |
| 344 | F2 | 5,23 |
| 345 | F2 | 4,45 |
| 346 | F2 | 4,3 |
| 347 | F2 | 4,45 |
| 348 | F2 | 4,05 |
| 349 | F2 | 6,26 |
| 350 | F2 | 4,8 |
| 351 | F2 | 4,7 |
| 352 | F2 | 4,64 |
| 353 | F2 | 4,01 |
| 354 | F2 | 5,12 |
| 355 | F2 | 6,05 |
| 356 | F2 | 5,1 |
| 357 | F2 | 5,23 |
| 358 | F2 | 4,83 |
| 359 | F2 | 4,56 |

| | | |
|---|---|---|
| 360 | F2 | 5,22 |
| 361 | F2 | 6,74 |
| 362 | F2 | 4,85 |
| 363 | F2 | 4,6 |
| 364 | F2 | 4,73 |
| 365 | F2 | 4,08 |
| 366 | F2 | 4,71 |
| 367 | F2 | 4,97 |
| 368 | F2 | 3,6 |
| 369 | F2 | 4,5 |
| 370 | F2 | 6,01 |
| 371 | F2 | 5,14 |
| 372 | F2 | 4,54 |
| 373 | F2 | 4,31 |
| 374 | F2 | 3,83 |
| 375 | F2 | 4,74 |
| 376 | F2 | 5,93 |
| 377 | F2 | 5,21 |
| 378 | F2 | 4,1 |
| 379 | F2 | 4,36 |
| 380 | F2 | 4 |
| 381 | F2 | 4,88 |
| 382 | F2 | 6,29 |
| 383 | F2 | 3,43 |
| 384 | F2 | 3,11 |
| 385 | F2 | 6,35 |
| 386 | F2 | 5,4 |
| 387 | F2 | 4,04 |
| 388 | F2 | 5,11 |
| 389 | F2 | 4,26 |
| 390 | F2 | 5,49 |
| 391 | F2 | 5,63 |
| 392 | F2 | 4,36 |
| 393 | F2 | 5,1 |
| 394 | F2 | 5,43 |
| 395 | F2 | 5,25 |
| 396 | F2 | 4,08 |
| 397 | F2 | 4,52 |
| 398 | F2 | 3,55 |
| 399 | F2 | 4,79 |
| 400 | F2 | 5,72 |
| 401 | F2 | 5,89 |
| 402 | F2 | 4,41 |
| 403 | F2 | 4,83 |
| 404 | F2 | 3,88 |

| | | |
|---|---|---|
| 405 | F2 | 4,62 |
| 406 | F2 | 4,89 |
| 407 | F2 | 5,55 |
| 408 | F2 | 3,89 |
| 409 | F2 | 3,97 |
| 410 | F2 | 4,05 |
| 411 | F2 | 5,22 |
| 412 | F2 | 6,23 |
| 413 | F2 | 4,95 |
| 414 | F2 | 5,39 |
| 415 | F2 | 6,26 |
| 416 | F2 | 4,1 |
| 417 | F2 | 4,8 |
| 418 | F2 | 6,07 |
| 419 | F2 | 5,27 |
| 420 | F2 | 5,76 |
| 421 | F2 | 6,29 |
| 422 | F2 | 3,57 |
| 423 | F2 | 4,27 |
| 424 | F2 | 4,99 |
| 425 | F2 | 5,61 |
| 426 | F2 | 5,22 |
| 427 | F2 | 6,36 |
| 428 | F2 | 3,66 |
| 429 | F2 | 5,38 |
| 430 | F2 | 4,74 |
| 431 | F2 | 4,31 |
| 432 | F2 | 5,99 |
| 433 | F2 | 6,61 |
| 434 | F2 | 5,17 |
| 435 | F2 | 4,59 |
| 436 | F2 | 3,98 |
| 437 | F2 | 4,76 |
| 438 | F2 | 5,82 |
| 439 | F2 | 5,87 |
| 440 | F2 | 5,14 |
| 441 | F2 | 4,74 |
| 442 | F2 | 4,42 |
| 443 | F2 | 5,83 |
| 444 | F2 | 6,09 |
| 445 | F2 | 3,6 |
| 446 | F2 | 4,84 |
| 447 | F2 | 5,61 |
| 448 | F2 | 4,97 |
| 449 | F2 | 4,27 |

| | | |
|---|---|---|
| 450 | F2 | 4,56 |
| 451 | F2 | 4,2 |
| 452 | F2 | 5,14 |
| 453 | F2 | 6,02 |
| 454 | F2 | 4,25 |
| 455 | F2 | 4,07 |
| 456 | F2 | 4,99 |
| 457 | F2 | 4,62 |
| 458 | F2 | 5,36 |
| 459 | F2 | 5,54 |
| 460 | F2 | 4,41 |
| 461 | F2 | 4,82 |
| 462 | F2 | 5,25 |
| 463 | F2 | 4,7 |
| 464 | F2 | 4,42 |
| 465 | F2 | 4,74 |
| 466 | F2 | 4,5 |
| 467 | F2 | 5,35 |
| 468 | F2 | 5,55 |
| 469 | F2 | 4,89 |
| 470 | F2 | 4,41 |
| 471 | F2 | 4,06 |
| 472 | F2 | 4,32 |
| 473 | F2 | 5,89 |
| 474 | F2 | 5,5 |
| 475 | F2 | 4,72 |
| 476 | F2 | 5,26 |
| 477 | F2 | 4,93 |
| 478 | F2 | 5,01 |
| 479 | F2 | 4,93 |
| 480 | F2 | 3,71 |
