# Supplementary material for: Does a gender of Welwitschia mirabilis plants influence their photosynthetic activity?
Source: PLoS One. 2023 Sep 8;18(9):e0291122. doi: 10.1371/journal.pone.0291122 (PMC10490862; doi:10.1371/journal.pone.0291122)
Supplement: S5 Table — (PDF) [file pone.0291122.s005.pdf]

Measurement number	Specimen number	Parameter g_s
1	M1	15,35
2	M1	15,64
3	M1	14,89
4	M1	14,95
5	M1	16,45
6	M1	14,62
7	M1	15,56
8	M1	14,19
9	M1	14,86
10	M1	14,96
11	M1	13,33
12	M1	14,26
13	M1	13,98
14	M1	13,94
15	M1	13,72
16	M1	14,6
17	M1	13,61
18	M1	13,54
19	M1	13,73
20	M1	13,19
21	M1	13,15
22	M1	13,61
23	M1	13,39
24	M1	14,39
25	M1	13
26	M1	12,7
27	M1	12,91
28	M1	12,16
29	M1	12,05
30	M1	13,25
31	M1	12,62
32	M1	13,08
33	M1	12,46
34	M1	12,23
35	M1	12,33
36	M1	11,97
37	M1	12,11
38	M1	12,31
39	M1	12,01
40	M1	12,13
41	M1	12,54
42	M1	11,29
43	M1	12,04
44	M1	11,87

45	M1	10,88
46	M1	11,63
47	M1	11,25
48	M1	10,74
49	M1	12,2
50	M1	11,8
51	M1	11,87
52	M1	11,83
53	M1	11,66
54	M1	11,37
55	M1	11,91
56	M1	11,06
57	M1	11,58
58	M1	11,74
59	M1	11,11
60	M1	11
61	M1	10,56
62	M1	10,58
63	M1	11,33
64	M1	10,95
65	M1	11,58
66	M1	10,99
67	M1	10,8
68	M1	11
69	M1	11,24
70	M1	10,63
71	M1	10,72
72	M1	10,79
73	M1	11,53
74	M1	11,43
75	M1	10,65
76	M1	10,43
77	M1	11,64
78	M1	9,84
79	M1	11,21
80	M1	10,61
81	M1	10,44
82	M1	11,51
83	M1	11,02
84	M1	10,5
85	M1	11,44
86	M1	10,53
87	M1	10,97
88	M1	11,11
89	M1	11,29

90	M1	10,68
91	M1	10,49
92	M1	11,36
93	M1	11,05
94	M1	9,5
95	M1	10,99
96	M1	11,04
97	M1	10,5
98	M1	10,16
99	M1	10,76
100	M1	10,73
101	M1	10,94
102	M1	10,42
103	M1	10,24
104	M1	10,6
105	M1	10,39
106	M1	10,93
107	M1	10,34
108	M1	9,97
109	M1	10,63
110	M1	10,86
111	M1	10,91
112	M1	10,67
113	M1	10,73
114	M1	10,49
115	M1	10,45
116	M1	10,55
117	M1	11,01
118	M1	11,22
119	M1	9,88
120	M1	11,25
121	M1	11,37
122	M1	10,33
123	M1	10,73
124	M1	11,06
125	M1	10,78
126	M1	10,16
127	M1	10,99
128	M1	10,65
129	M1	10,88
130	M1	10,4
131	M1	10,37
132	M1	10,93
133	M1	10,31
134	M1	10,63

135	M1	10,52
136	M1	9,79
137	M1	10,45
138	M1	11,44
139	M1	10,21
140	M1	11,16
141	M1	10,6
142	M1	10,51
143	M1	10,78
144	M1	10,57
145	M1	10,91
146	M1	11,48
147	M1	10,67
148	M1	10,06
149	M1	11,37
150	M1	10,32
151	M1	11,38
152	M1	11,71
153	M1	11,55
154	M1	10,57
155	M1	11,23
156	M1	11,78
157	M1	11,42
158	M1	11,52
159	M1	10,87
160	M1	12,01
161	M1	11,07
162	M1	11,43
163	M1	11,84
164	M1	10,55
165	M1	12,32
166	M1	12,4
167	M1	11,13
168	M1	11,8
169	M1	11,41
170	M1	11,65
171	M1	11,45
172	M1	11,03
173	M1	12,56
174	M1	12,1
175	M1	11,2
176	M1	11,56
177	M1	12,12
178	M1	11,64
179	M1	11,63

180	M1	12,17
181	M1	11,84
182	M1	12,27
183	M1	11,6
184	M1	11,01
185	M1	11,01
186	M1	11,36
187	M1	11,56
188	M1	11,49
189	M1	12,02
190	M1	11,9
191	M1	12,84
192	M1	11,38
193	M1	11,52
194	M1	12,36
195	M1	11,98
196	M1	12,06
197	M1	11,88
198	M1	11,8
199	M1	12,28
200	M1	12,22
201	M1	11,87
202	M1	11,94
203	M1	11,31
204	M1	12,39
205	M1	12,67
206	M1	12,35
207	M1	12,13
208	M1	11,81
209	M1	11,21
210	M1	12,86
211	M1	12,24
212	M1	12,28
213	M1	12,21
214	M1	11,95
215	M1	11,36
216	M1	12,48
217	M1	11,71
218	M1	11,83
219	M1	12,24
220	M1	11,79
221	M1	11,52
222	M1	12,17
223	M1	12,3
224	M1	11,34

225	M1	11,78
226	M1	11,56
227	M1	12,09
228	M1	11,93
229	M1	12,45
230	M1	12,36
231	M1	11,82
232	M1	12,29
233	M1	12,55
234	M1	11,37
235	M1	11,99
236	M1	12,26
237	M1	11,6
238	M1	12,77
239	M1	12,36
240	F2	11,64
241	F2	10,66
242	F2	10,61
243	F2	10,48
244	F2	11,11
245	F2	11,4
246	F2	10,91
247	F2	12,02
248	F2	11,69
249	F2	11,63
250	F2	12,83
251	F2	12,44
252	F2	12,24
253	F2	12,24
254	F2	12,62
255	F2	12,69
256	F2	13,06
257	F2	13,72
258	F2	13,61
259	F2	13,87
260	F2	15,09
261	F2	13,77
262	F2	14,26
263	F2	15,26
264	F2	14,58
265	F2	15,52
266	F2	16,38
267	F2	15,73
268	F2	16,34
269	F2	17,35

270	F2	16,99
271	F2	17,77
272	F2	18,46
273	F2	17,46
274	F2	18,68
275	F2	19,71
276	F2	18,38
277	F2	19,69
278	F2	20,68
279	F2	19,68
280	F2	20,87
281	F2	21,94
282	F2	21,38
283	F2	22,34
284	F2	22,47
285	F2	22,43
286	F2	22,71
287	F2	23,68
288	F2	23,91
289	F2	24,2
290	F2	25,17
291	F2	25,49
292	F2	25,25
293	F2	25,85
294	F2	26,39
295	F2	28,55
296	F2	27,57
297	F2	26,94
298	F2	28,12
299	F2	28,72
300	F2	28,62
301	F2	29,57
302	F2	30,18
303	F2	29,57
304	F2	30,71
305	F2	30,92
306	F2	31,66
307	F2	32,73
308	F2	32,44
309	F2	32,33
310	F2	33,5
311	F2	34,02
312	F2	33,82
313	F2	34,77
314	F2	34,94

315	F2	33,99
316	F2	34,7
317	F2	36,19
318	F2	35,42
319	F2	35,9
320	F2	36,4
321	F2	37,54
322	F2	37,58
323	F2	37,41
324	F2	37,06
325	F2	38,19
326	F2	39,28
327	F2	39,11
328	F2	37,89
329	F2	39,77
330	F2	40,48
331	F2	39,98
332	F2	39,77
333	F2	40,48
334	F2	40,97
335	F2	40,05
336	F2	41,21
337	F2	42,03
338	F2	41,62
339	F2	42,99
340	F2	42,04
341	F2	42
342	F2	42,79
343	F2	44,16
344	F2	43,63
345	F2	42,82
346	F2	43,68
347	F2	42,74
348	F2	44,7
349	F2	45,37
350	F2	45,06
351	F2	45,46
352	F2	44,92
353	F2	45,41
354	F2	46,15
355	F2	46,05
356	F2	45,79
357	F2	46,37
358	F2	47,17
359	F2	46,64

360	F2	46,9
361	F2	47,95
362	F2	46,98
363	F2	47,3
364	F2	47,58
365	F2	47,74
366	F2	47,39
367	F2	49,37
368	F2	47,77
369	F2	49,73
370	F2	49,97
371	F2	49,3
372	F2	48,41
373	F2	48,3
374	F2	49,49
375	F2	50,67
376	F2	50,96
377	F2	50,19
378	F2	49,93
379	F2	50,68
380	F2	49,93
381	F2	50,72
382	F2	52,32
383	F2	50,44
384	F2	50,86
385	F2	53,04
386	F2	52,29
387	F2	51,29
388	F2	52,62
389	F2	52,01
390	F2	52,91
391	F2	53,53
392	F2	52,6
393	F2	53,05
394	F2	53,87
395	F2	53,75
396	F2	53,24
397	F2	52,56
398	F2	52,67
399	F2	54,39
400	F2	54,5
401	F2	55,35
402	F2	54,71
403	F2	53,51
404	F2	53,44

405	F2	54,48
406	F2	55,38
407	F2	55,16
408	F2	54,4
409	F2	53,78
410	F2	54,4
411	F2	55,7
412	F2	55,29
413	F2	55,87
414	F2	56,02
415	F2	55,64
416	F2	54,98
417	F2	55,27
418	F2	55,5
419	F2	55,9
420	F2	55,96
421	F2	55,85
422	F2	54,69
423	F2	55,12
424	F2	56,57
425	F2	55,92
426	F2	57,22
427	F2	56,92
428	F2	54,32
429	F2	56,3
430	F2	55,1
431	F2	56,12
432	F2	56,43
433	F2	56,84
434	F2	55,97
435	F2	56,45
436	F2	55,91
437	F2	56,5
438	F2	56,86
439	F2	56,26
440	F2	57,26
441	F2	56,08
442	F2	55,56
443	F2	61,75
444	F2	61,49
445	F2	59,72
446	F2	61,3
447	F2	61,71
448	F2	60,25
449	F2	60,73

450	F2	60,94
451	F2	60,19
452	F2	61,8
453	F2	61,82
454	F2	59,93
455	F2	60,93
456	F2	60,23
457	F2	60,18
458	F2	61,84
459	F2	61,6
460	F2	60,56
461	F2	60,41
462	F2	60,72
463	F2	60,44
464	F2	59,87
465	F2	60,05
466	F2	60,3
467	F2	61,23
468	F2	62,03
469	F2	60,73
470	F2	59,2
471	F2	59,06
472	F2	59,56
473	F2	61,36
474	F2	60,05
475	F2	60,01
476	F2	59,97
477	F2	59,69
478	F2	59,72
479	F2	60,95
480	F2	60,42
