# Supplementary material for: Does a gender of Welwitschia mirabilis plants influence their photosynthetic activity?
Source: PLoS One. 2023 Sep 8;18(9):e0291122. doi: 10.1371/journal.pone.0291122 (PMC10490862; doi:10.1371/journal.pone.0291122)
Supplement: S6 Table — (PDF) [file pone.0291122.s006.pdf]

Measurement number	Specimen number	Parameter C_i
1	M1	298,3
2	M1	316
3	M1	343,3
4	M1	258,4
5	M1	366,5
6	M1	318,6
7	M1	289,9
8	M1	335,8
9	M1	252,8
10	M1	240,7
11	M1	282,7
12	M1	321,3
13	M1	258,3
14	M1	313,7
15	M1	277
16	M1	289,3
17	M1	329,9
18	M1	278,7
19	M1	251,7
20	M1	283,6
21	M1	227,1
22	M1	299,5
23	M1	280,8
24	M1	256
25	M1	271,7
26	M1	293,5
27	M1	236,5
28	M1	321,2
29	M1	259,8
30	M1	261
31	M1	322,6
32	M1	281,7
33	M1	202,7
34	M1	301,9
35	M1	229,6
36	M1	258,4
37	M1	267,4
38	M1	248,5
39	M1	227,1
40	M1	261,7
41	M1	204
42	M1	267,4
43	M1	294

44	M1	219,6
45	M1	297,9
46	M1	264,1
47	M1	239,3
48	M1	271,6
49	M1	283,7
50	M1	211,4
51	M1	296,1
52	M1	260,5
53	M1	195,9
54	M1	276,2
55	M1	272,6
56	M1	240,2
57	M1	279,7
58	M1	247,4
59	M1	249,3
60	M1	269
61	M1	224,3
62	M1	287,4
63	M1	265,1
64	M1	174,5
65	M1	267,9
66	M1	232
67	M1	180,2
68	M1	257,4
69	M1	281,7
70	M1	188,6
71	M1	279,3
72	M1	253,9
73	M1	166
74	M1	292,2
75	M1	259,3
76	M1	231
77	M1	259,1
78	M1	253,5
79	M1	200,2
80	M1	264,6
81	M1	194,9
82	M1	276,8
83	M1	287,1
84	M1	224,4
85	M1	276
86	M1	270,7
87	M1	224,6
88	M1	291,4

89	M1	214,1
90	M1	230,1
91	M1	268,5
92	M1	237,6
93	M1	246,3
94	M1	302,5
95	M1	228,4
96	M1	198,9
97	M1	336,2
98	M1	210,7
99	M1	297,6
100	M1	267
101	M1	229,5
102	M1	248,5
103	M1	312
104	M1	232,7
105	M1	247,2
106	M1	277,4
107	M1	292,2
108	M1	292,8
109	M1	297,8
110	M1	239,5
111	M1	286,8
112	M1	284,9
113	M1	186,9
114	M1	343,9
115	M1	252,2
116	M1	261,1
117	M1	362
118	M1	235,3
119	M1	205,7
120	M1	352,6
121	M1	329,8
122	M1	257,5
123	M1	368,3
124	M1	226,9
125	M1	214,2
126	M1	294,5
127	M1	330,6
128	M1	338,3
129	M1	333,4
130	M1	223,5
131	M1	362,8
132	M1	326,7
133	M1	254,7

134	M1	365,8
135	M1	321,1
136	M1	217
137	M1	399,3
138	M1	306
139	M1	269,4
140	M1	373,7
141	M1	343,9
142	M1	214,9
143	M1	394,5
144	M1	361,9
145	M1	261,9
146	M1	304,9
147	M1	242,1
148	M1	351
149	M1	370,5
150	M1	239,3
151	M1	348,8
152	M1	345,7
153	M1	238,4
154	M1	345,7
155	M1	336,4
156	M1	383,5
157	M1	354,5
158	M1	193,2
159	M1	358,7
160	M1	317,3
161	M1	214,3
162	M1	381,2
163	M1	313,4
164	M1	342,5
165	M1	304,4
166	M1	310,4
167	M1	295,7
168	M1	314,6
169	M1	369,8
170	M1	380,7
171	M1	306,3
172	M1	296,3
173	M1	301,6
174	M1	275
175	M1	262,8
176	M1	308,7
177	M1	361,3
178	M1	249,2

179	M1	373,2
180	M1	257,6
181	M1	295,1
182	M1	382,1
183	M1	281
184	M1	388,9
185	M1	337,4
186	M1	269,6
187	M1	373,4
188	M1	292,3
189	M1	189,3
190	M1	287,7
191	M1	291,7
192	M1	274,6
193	M1	370,6
194	M1	311,5
195	M1	309,1
196	M1	326,3
197	M1	251,6
198	M1	341,5
199	M1	326,6
200	M1	323,7
201	M1	348,3
202	M1	277,8
203	M1	371,3
204	M1	357,9
205	M1	363,9
206	M1	344,4
207	M1	383
208	M1	349,6
209	M1	381,4
210	M1	290
211	M1	382,1
212	M1	348,3
213	M1	228,3
214	M1	331,4
215	M1	296
216	M1	245,2
217	M1	361,7
218	M1	308,2
219	M1	273,5
220	M1	376,3
221	M1	313,9
222	M1	283,8
223	M1	336,5

224	M1	328,9
225	M1	321,3
226	M1	367,6
227	M1	318,5
228	M1	367,9
229	M1	399,1
230	M1	261,1
231	M1	331,2
232	M1	313,5
233	M1	313,9
234	M1	354,4
235	M1	319,1
236	M1	313,7
237	M1	375
238	M1	299,6
239	M1	399,9
240	F2	317
241	F2	344,8
242	F2	264,2
243	F2	328
244	F2	239,8
245	F2	162,2
246	F2	251,8
247	F2	389,9
248	F2	252,6
249	F2	198,6
250	F2	395,1
251	F2	382,9
252	F2	348
253	F2	321,8
254	F2	255,5
255	F2	225,2
256	F2	360,2
257	F2	334
258	F2	335
259	F2	341,6
260	F2	219,8
261	F2	366,1
262	F2	273,2
263	F2	325,4
264	F2	284,5
265	F2	185,6
266	F2	356,5
267	F2	206,9
268	F2	155,3

269	F2	342
270	F2	293,1
271	F2	236,6
272	F2	355
273	F2	320,2
274	F2	204,2
275	F2	361,5
276	F2	281,1
277	F2	214,6
278	F2	375,9
279	F2	300,1
280	F2	163,7
281	F2	351
282	F2	270,7
283	F2	273,7
284	F2	311,7
285	F2	338,5
286	F2	307,1
287	F2	342,2
288	F2	296,6
289	F2	257,2
290	F2	275,5
291	F2	239,2
292	F2	276,3
293	F2	251,1
294	F2	220
295	F2	258,7
296	F2	330
297	F2	279,4
298	F2	185,9
299	F2	330,5
300	F2	290,5
301	F2	286,5
302	F2	288,2
303	F2	295,7
304	F2	259,8
305	F2	308,1
306	F2	293,3
307	F2	287,5
308	F2	280,4
309	F2	255,7
310	F2	270,8
311	F2	320,4
312	F2	218,4
313	F2	225,4

314	F2	329,4
315	F2	296,8
316	F2	270,3
317	F2	309,3
318	F2	300,3
319	F2	275,7
320	F2	293,8
321	F2	248,8
322	F2	278,4
323	F2	325,7
324	F2	247,6
325	F2	283,3
326	F2	292,2
327	F2	298,4
328	F2	292
329	F2	270,8
330	F2	254,5
331	F2	295,1
332	F2	291,2
333	F2	304,9
334	F2	318,6
335	F2	302,5
336	F2	251,6
337	F2	281,7
338	F2	302,3
339	F2	267,1
340	F2	324,6
341	F2	312,2
342	F2	245,7
343	F2	273,5
344	F2	297,8
345	F2	307,1
346	F2	298,8
347	F2	317,3
348	F2	245,4
349	F2	295,1
350	F2	299,3
351	F2	298,2
352	F2	321,8
353	F2	286,3
354	F2	255,1
355	F2	288
356	F2	284,9
357	F2	300,6
358	F2	306,7

359	F2	285,5
360	F2	240
361	F2	299,6
362	F2	309,1
363	F2	304,9
364	F2	325,8
365	F2	303,2
366	F2	303,8
367	F2	342,2
368	F2	317,6
369	F2	271,9
370	F2	297,6
371	F2	313,9
372	F2	320
373	F2	338,3
374	F2	311,1
375	F2	277,8
376	F2	298
377	F2	330,5
378	F2	323,9
379	F2	332,5
380	F2	306,7
381	F2	271,7
382	F2	351,3
383	F2	362,9
384	F2	272
385	F2	299,7
386	F2	335,2
387	F2	308,8
388	F2	330,4
389	F2	298,2
390	F2	294,8
391	F2	329,1
392	F2	304,6
393	F2	300,7
394	F2	306,9
395	F2	340,1
396	F2	324,1
397	F2	352,7
398	F2	320
399	F2	297
400	F2	295,1
401	F2	334,4
402	F2	321,6
403	F2	346

404	F2	325,6
405	F2	324,5
406	F2	303
407	F2	350,9
408	F2	346,1
409	F2	342,3
410	F2	312,6
411	F2	285,7
412	F2	323,2
413	F2	310,7
414	F2	285,6
415	F2	344,1
416	F2	325,2
417	F2	292,9
418	F2	316,9
419	F2	300,2
420	F2	286,4
421	F2	357,1
422	F2	339,1
423	F2	323,8
424	F2	304,6
425	F2	318
426	F2	286,3
427	F2	354,3
428	F2	310,9
429	F2	326,9
430	F2	340,7
431	F2	296,4
432	F2	279,3
433	F2	316,7
434	F2	335,5
435	F2	349,5
436	F2	328,2
437	F2	299,8
438	F2	298,7
439	F2	321,5
440	F2	330,1
441	F2	337,2
442	F2	320,9
443	F2	314,4
444	F2	375,4
445	F2	346,9
446	F2	327
447	F2	338,9
448	F2	359,1

449	F2	352,1
450	F2	360,1
451	F2	338,2
452	F2	316,2
453	F2	357,1
454	F2	366,4
455	F2	342
456	F2	349,8
457	F2	334,5
458	F2	329,9
459	F2	356,3
460	F2	347,1
461	F2	335
462	F2	348,3
463	F2	355,3
464	F2	346,7
465	F2	353,1
466	F2	334,1
467	F2	331,3
468	F2	344,9
469	F2	355,4
470	F2	362,6
471	F2	356,7
472	F2	319,2
473	F2	328,6
474	F2	348,1
475	F2	334,1
476	F2	341
477	F2	337,9
478	F2	341,9
479	F2	372,9
