# Supplementary material for: Does a gender of Welwitschia mirabilis plants influence their photosynthetic activity?
Source: PLoS One. 2023 Sep 8;18(9):e0291122. doi: 10.1371/journal.pone.0291122 (PMC10490862; doi:10.1371/journal.pone.0291122)
Supplement: S7 Table — (PDF) [file pone.0291122.s007.pdf]

Measurement number	Specimen number	Number of stomata on abaxial side of a leaf	Number of stomata on adaxial side of a leaf
1	F1	12250	16200
2	F1	13500	17000
3	F1	10900	14900
4	F1	10150	14950
5	F2	11250	15900
6	F2	11190	14920
7	M1	13000	10950
8	M1	12250	12300
9	M1	11900	12100
10	M1	13100	10950
11	M2	14800	13700
12	M2	14400	14000
13	M2	13150	13700
14	M2	13550	13500
