# Supplementary material for: Does a gender of Welwitschia mirabilis plants influence their photosynthetic activity?
Source: PLoS One. 2023 Sep 8;18(9):e0291122. doi: 10.1371/journal.pone.0291122 (PMC10490862; doi:10.1371/journal.pone.0291122)
Supplement: S8 Table — (PDF) [file pone.0291122.s008.pdf]

Measurement number	Specimen number	NDVI coefficient
1	F1	0,67738
2	F1	0,673107
3	F1	0,673937
4	F1	0,696168
5	F1	0,683273
6	F1	0,695336
7	F1	0,699691
8	F1	0,687051
9	F1	0,704558
10	F1	0,686286
11	F2	0,724719
12	F2	0,719798
13	F2	0,71661
14	F2	0,706205
15	F2	0,702568
16	F2	0,717816
17	F2	0,722147
18	F2	0,70613
19	F2	0,716418
20	F2	0,705283
21	M1	0,699948
22	M1	0,700105
23	M1	0,70162
24	M1	0,700315
25	M1	0,697385
26	M1	0,713731
27	M1	0,691059
28	M1	0,681845
29	M1	0,714842
30	M1	0,691583
31	M2	0,716554
32	M2	0,73124
33	M2	0,730255
34	M2	0,729273
35	M2	0,701249
36	M2	0,701175
37	M2	0,721828
38	M2	0,726471
39	M2	0,705378
40	M2	0,697916
41	F3	0,717992
42	F3	0,732112
43	F3	0,703569
44	F3	0,718871

45	F3	0,695544
46	F3	0,699904
47	F3	0,698784
48	F3	0,700111
49	F3	0,700583
50	F3	0,706154
51	M3	0,721438
52	M3	0,728375
53	M3	0,739598
54	M3	0,72107
55	M3	0,712719
56	M3	0,728219
57	M3	0,711802
58	M3	0,70233
59	M3	0,724386
60	M3	0,727177
61	F4	0,702957
62	F4	0,709735
63	F4	0,689045
64	F4	0,696097
65	F4	0,694739
66	F4	0,714403
67	F4	0,719173
68	F4	0,714211
69	F4	0,728438
70	F4	0,724072
71	M4	0,70472
72	M4	0,704203
73	M4	0,699648
74	M4	0,7041
75	M4	0,704524
76	M4	0,70915
77	M4	0,708367
78	M4	0,708643
79	M4	0,715496
80	M4	0,708866
81	M5	0,686102
82	M5	0,674292
83	M5	0,683023
84	M5	0,687669
85	M5	0,694877
86	M5	0,673781
87	M5	0,685799
88	M5	0,672244
89	M5	0,690696

90	M5	0,683665
91	F5	0,650137
92	F5	0,652069
93	F5	0,653096
94	F5	0,651601
95	F5	0,648901
96	F5	0,683134
97	F5	0,672228
98	F5	0,688649
99	F5	0,687008
100	F5	0,674101
101	F6	0,694813
102	F6	0,690479
103	F6	0,674585
104	F6	0,699224
105	F6	0,691017
106	F6	0,711019
107	F6	0,728258
108	F6	0,711882
109	F6	0,725422
110	F6	0,71234
